# Stereoselective chemoenzymatic phytate transformations provide access to diverse inositol phosphate derivatives†

**DOI:** 10.1039/d5sc02844b

**Published:** 2025-06-23

**Authors:** Georg Markus Häner, Guizhen Liu, Esther Lange, Nikolaus Jork, Klaus Ditrich, Ralf Greiner, Gabriel Schaaf, Henning J. Jessen

**Affiliations:** a Institute of Organic Chemistry, University of Freiburg Germany henning.jessen@oc.uni-freiburg.de; b Institute of Crop Science and Resource Conversation, Department of Plant Nutrition, University of Bonn Germany; c White Biotechnology Research – Biocatalysis BASF SE, A030 Carl-Bosch-Strasse 38 67056 Ludwigshafen am Rhein Germany; d Department of Food Technology and Bioprocess Engineering, Max Rubner-Institut, Federal Research Institute of Nutrition and Food Haid-und-Neu-Straße 9 Karlsruhe 76131 Germany; e CIBSS – Centre for Integrative Biological Signaling Studies Freiburg Germany

## Abstract

Phosphorylated *myo*-inositols (InsPs) are essential cytoplasmic signaling molecules, while their lipidated analogs (PtdInsPs) play a crucial role in membrane signaling. Stereoselective synthesis of these compounds has been achieved through various methods, predominantly using the *meso* compound *myo*-inositol as a starting material. However, phytate (InsP_6_), also a *meso* compound, is the most abundant inositol derivative in plants – far more prevalent than *myo*-inositol itself. Despite its abundance, phytate has been rarely used in synthetic strategies for accessing a variety of chiral inositol phosphates and their derivatives through selective dephosphorylations on a preparative scale. Here, we report gram-scale (stereo)selective dephosphorylations of phytate using phytases and demonstrate the application of these products in generating modified InsPs through a transient phosphitylation approach. Notably, the bacterial effector XopH efficiently desymmetrizes *meso*-phytate to yield enantiomerically pure 1-OH-InsP_5_. This transformation renders the 1-position accessible for further modifications, which, in biological systems, is where glycerolphosphate diesters are attached. By using selective dephosphorylations with phytases in concert with chemoselective telescoping reaction sequences, this approach greatly advances the stereoselective synthesis of inositol phosphates and their derivatives, such as glycerophosphoinositols, from abundant InsP_6_.

## Abstract 2

Phosphorylated *myo*-inositols (InsPs) and their lipidated analogs (PtdInsPs) are crucial signaling molecules. Here, a chemoenzymatic approach towards these molecules using enantioselective dephosphorylations and phosphorylations is established.

## Graphical abstract

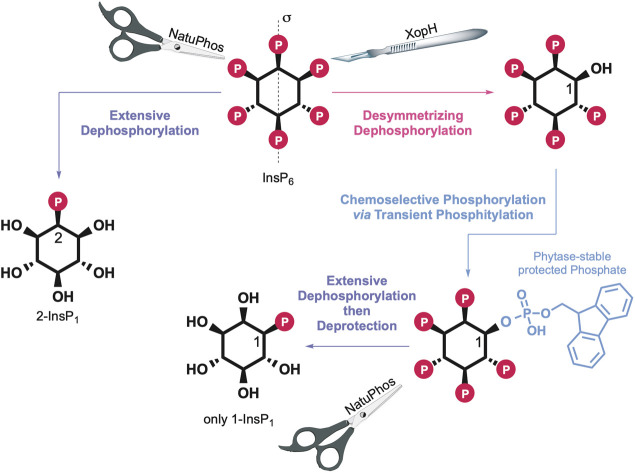

## Introduction

Westheimer famously stated “phosphate esters and anhydrides dominate the living world”, but the less-cited second half of his statement adds that they “are seldom used as intermediates by organic chemists”.^1^ This is still true to date. Here, we address this disparity in the context of *myo*-inositol phosphate chemistry. *Myo*-inositol (hereafter inositol) phosphates (InsP_*x*_), inositol-pyrophosphates (PP-InsP_*x*_) and their membrane bound brethren (phosphatidyl inositol phosphates, PtdInsP_*x*_) are a family of highly phosphorylated second messenger molecules, influencing an enormous variety of biological processes (*e.g.* phosphate homeostasis or membrane trafficking events).^2–6^ Recent analytical advances propelled the field forward, (re)discovering previously understudied isomers (*e.g.* different PP-InsP's)^7^, and metabolites (*e.g.* 2-InsP_1_ or 1,2,3-InsP_3_)^8,9^ in biological samples *via* MS^10–12^- and NMR^13^ based methods.

To elucidate the identity of the signaling molecules and understand their precise biological functions, the synthesis of defined isomers and analogs is crucial.^2,3,6,14^ Typically, desymmetrization of the parent *meso* compound *myo*-inositol is considered as a reliable entry point.^15,16^ This *meso* trick is often performed with the help of chiral auxiliaries, allowing the synthesis of *e.g.* InsP_7_ (ref. 17–20) and PtdInsP^21–24^ isomers relying on asymmetric phosphorylations.^20,23,25^ Desymmetrizations with peptide catalysts,^26–31^ for selective phosphorylations, or the use of lipases,^21,32,33^ for selective esterifications, were established as alternatives to chiral auxiliaries. The use of chemoenzymatic approaches, including dioxygenases, for the stereoselective synthesis of inositol derivatives from distant precursors like benzene, has been comprehensively reviewed.^34^ In summary, the desymmetrization of *myo*-inositol has been established as a corner stone of diverse synthetic approaches towards InsP and PtdInsP derivatives.

In contrast, the complementary approach – desymmetrization of the fully phosphorylated *meso* compound InsP_6_*via* selective dephosphorylations as a starting point for synthesis – is almost completely absent from literature. This is not understandable from a supply perspective: InsP_6_ is the major phosphate storage molecule in plants and is available on a large scale. In fact, it is a far more abundant starting material compared to inositol itself. However, chemistry needs to be developed further to transform the highly charged polyphosphorylated intermediates that would arise from selective phytate dephosphorylations, which we identify as one major obstacle in this approach, in line with the initial statement by Westheimer.

**Scheme 1:**
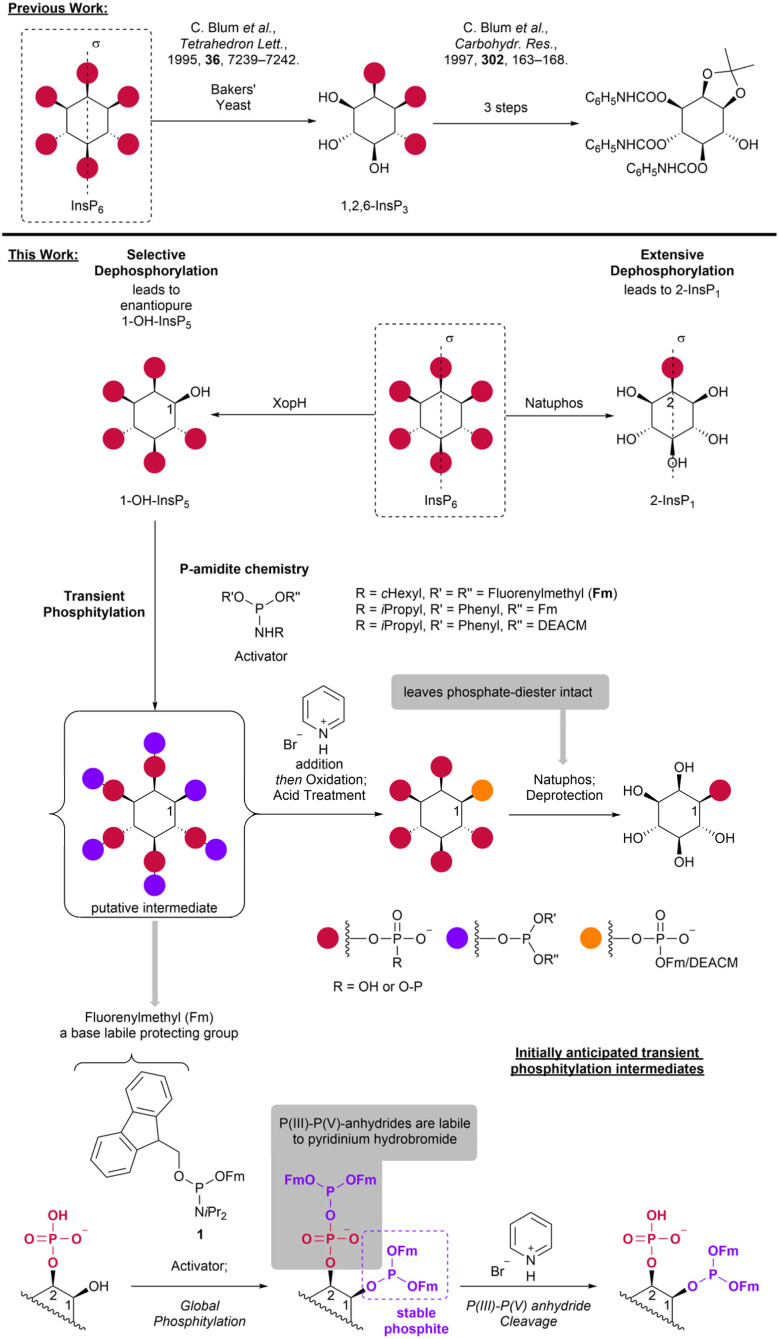
Desymmetrization of phytate *via* selective dephosphorylation using phytases. Previously, 1,2,6-InsP_3_ was obtained by baker's yeast digest.^35,36^ Here, selective dephosphorylation with expressed XopH on a preparative scale was established. The resulting chiral 1-OH-InsP_5_ was transformed into the corresponding chiral 1-InsP_1_ chemoenzymatically with the phytase Natuphos, thus inverting the phosphorylation pattern.

In the 90's, selective phytate dephosphorylation with baker's yeast leading mainly to 1,2,6-InsP_3_ ^35,36^ was reported (Scheme 1). The synthetic utility of the resulting chiral trisphosphate was proven in the generation of diverse therapeutically and synthetically relevant derivatives *via* oxidation,^37^*O*-acylation,^38^ carbamoylation,^35^*P*-alkylation^39^ and ultimately acidic phosphate cleavage.^35,36^ In 1999 selective dephosphorylation of 1,4,5,6-InsP_4_ (or enantiomeric 3,4,5,6-InsP_4_) with InsP_5_/InsP_4_-phosphohydrolase to 1,5,6-InsP_3_ (or 3,4,5-InsP_3_ respectively) was shown, highlighting other synthetic applications of selective enzymatic dephosphorylations. However, the required starting materials had to be synthesized in enantiomerically pure form starting from hydroquinone.^40^

Here, we present a dephosphorylation approach to enzymatically desymmetrize abundant *meso*-phytate and use the single product as an entry point to access phosphorylated inositol derivatives (see Scheme 1 for an overview). We rely on the recently discovered unique phytase activity of XopH, a bacterial effector protein found in *Xanthomonas euvesicatoria*. XopH dephosphorylates InsP_6_ exclusively at the 1-position, resulting in enantiopure chiral 1-hydroxy-inositol-pentakisphosphate (1-OH-InsP_5_).^41^ Notably, the 1-position holds particular significance in InsP chemistry, serving as the attachment point for the glycerolphosphate diester in PtdInsPs.

Additionally, we demonstrate that commercial Natuphos, originating from *Aspergillus ficuum*, has potential in InsP chemistry, although no desymmetrization is observed in this case, as the sole product is the *meso* compound 2-InsP_1_, in accordance with observations in naturally occurring phytases of varying provenance.^42–48^ This commercial phytase was previously compared to the follow-up product Natuphos E for the synthesis of InsP_3–5_*via* selective dephosphorylations.^49^

The products of the enzymatic dephosphorylations are then transformed into other phosphorylated inositols using a transient phosphitylation approach, that relies on the lability of mixed P(iii)–P(v) anhydrides in contrast to the higher stability of P(iii) triesters. Natuphos can then be used to remove unwanted phosphates and thus invert the phosphorylating pattern of the starting material (Scheme 1).

## Results and discussion

**Scheme 2:**
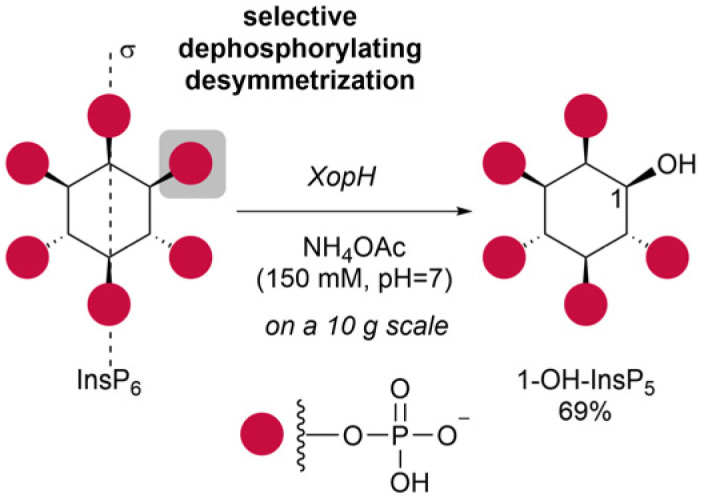
Synthesis of 1-OH-InsP_5_2*via* selective dephosphorylation of InsP_6_ with XopH.

1-OH-InsP_5_ has previously been obtained by selective dephosphorylation using the bacterial effector XopH on a small scale *in vitro* to assign enzyme specificity.^41^ We have now scaled this reaction to access 1-OH-InsP_5_ as starting material for further chemical transformations (Scheme 2).

During scale-up it was observed that larger amounts of phytate in the reaction led to some precipitation. To account for possible XopH precipitation, the reaction time was extended accordingly and complete consumption of InsP_6_ was observed after 8 h.

The crude enzymatic digest of InsP_6_ was purified by strong anion exchange (SAX) chromatography on a Q-sepharose column using an NH_4_HCO_3_ (1 M, pH = 8.0) gradient as eluent. Next, to ensure solubility in organic solvents for further transformations, 1-OH-InsP_5_ was converted to its tetrabutylammonium (TBA) salt.

One of our goals was to install a phosphate diester in the 1-position of 1-OH InsP_5_ as this would enable direct access to PtdInsPs and analogues, which was previously difficult to achieve. It is known that phosphates react rapidly with other P-derived electrophiles. For example, unprotected nucleoside phosphates (with free OH groups) can be phosphorylated with P-amidites.^50^ Also P-imidazolides^51–57^ and diamidophosphates^58^ can be used to construct P-anhydrides even in water as solvent with high chemoselectivity. This suggests that a chemical modification of phosphorylated inositols, such as 1-OH-InsP_5_, will first take place at the phosphate esters, before the alcohol will react.

This has significant implications regarding reaction design. Here, we focused on the use of P-amidites, with the expectation that initially P(iii)–P(v) anhydrides would form, followed by a reaction of the OH group forming a P(iii) triester. A strategy was therefore required to selectively cleave the mixed anhydrides again after transiently blocking the P(v) esters, while preserving the P(iii) triester – an approach that would have to be based on transient phosphitylation. To identify mild conditions that enable the cleavage of a P(iii)–P(v) anhydride, adenosine diphosphate (ADP) was used as a model compound (see ESI Fig. 1†). Screening revealed that pyridinium hydrobromide (in the following Pyr × HBr) rapidly cleaves the mixed anhydride. This approach was then applied to 1-OH-InsP_5_, enabling selective modification at the 1-position while ensuring transient P(iii)–P(v) anhydrides were cleaved in the presence of Pyr × HBr.

**Scheme 3:**
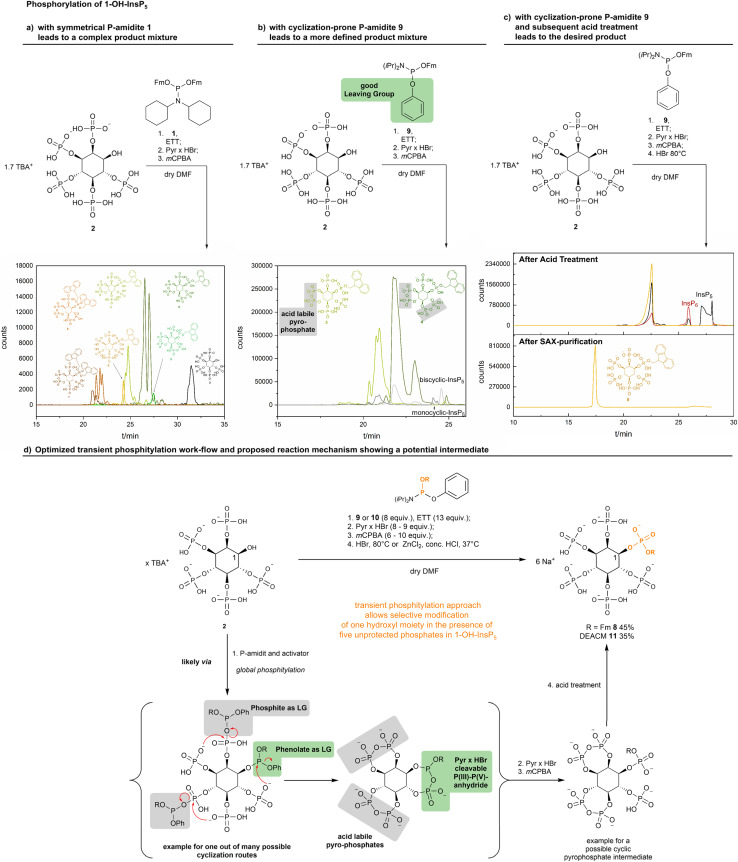
Synthesis of InsP_6_ derivatives modified solely at the 1-position. CE-qTOF-MS (background electrolyte (in the following BGE): NH_4_OAc 35 mM pH = 9.7, CE voltage: 30 kV, CE current: 23 μA, injection: 100 mbar, 15 s (30 nL)) analysis of the transient phosphitylation of 1-OH-InsP_5_ using different P-amidites. The depicted structures are color coded by identical mass. (a) Using P-amidite 1 a complex mixture was obtained. (b) Reduction of components was achieved *via* controlled cyclization using cyclization prone P-amidite 9. The depicted cyclic pyrophosphates are just examples of possible structures. (c) The obtained cyclic intermediates were transformed into a single product *in situ via* subsequent acid treatment. (d) Using an optimized transient phosphitylation work-flow 1-Fm-InsP_6_8 and 1-DEACM-InsP_6_11 were synthesized in one telescoping reaction sequence starting from 1-OH-InsP_5_.

1-OH-InsP_5_ TBA salt 2 was reacted with an excess of bis-fluorenylmethyl-P-amidite 1 (see ESI Fig. 2,† fluorenylmethyl = Fm) and was then analyzed by ^31^P-NMR spectroscopy. Due to the number of phosphates in the starting material 2, several P(iii)–P(v)-anhydride signals were observed, as indicated by the characteristic chemical shifts in ^31^P-NMR (125 to 130 ppm).^59^ Addition of pyridinium hydrobromide led to complete disappearance of the P(iii)–P(v)-anhydride signals, indicating cleavage. However, subsequent oxidation using *m*CPBA led to a complex mixture. To understand the failure of the desired synthesis, the crude material was analyzed by capillary electrophoresis mass spectrometry (CE-MS).^7^ We found a variety of InsP_6_ derivatives where adjacent phosphate groups had undergone condensation reactions to cyclic pyrophosphate derivatives. Moreover, the products were bearing either one or two Fm groups (Scheme 3a). Optimization of the reaction conditions did neither lead to reduced cyclizations nor reduced Fm cleavage. As an alternative, we reasoned that a controlled cyclization should furnish a more defined cyclic pyrophosphate mixture, and a controlled hydrolysis of those intermediates could then result in a defined product.

P-amidite 9, containing a phenyl moiety as a good leaving group instead of Fm, was designed and synthesized to promote the cyclization reactions (Scheme 3b). Interestingly, no P(iii)–P(v) anhydrides were detectable by ^31^P-NMR after global phosphitylation (see ESI Fig. 3†). However, addition of Pyr × HBr was necessary to assure quantitative decomposition of putatively *in situ* formed P(iii)–P(v) anhydrides. The reaction outcome was analyzed by CE-MS. Under these new conditions a much more defined mixture of only mono- and bis-cyclic InsP_6_ derivatives bearing exactly one Fm-group were obtained after oxidation. While the number of possible products decreased drastically, it was still not possible to identify the formed isomers. This assignment would also not be necessary, if it were possible to hydrolyze the cyclic pyrophosphates. One would expect the mixture to converge into a single product: InsP_6_ with an Fm protecting group located at the phosphate in the 1-position. Cyclic pyrophosphate hydrolysis was therefore studied next using inositoltrispyrophosphate (ITPP) as a model compound (see ESI Tables 4, 5 and Fig. 4†).^60,61^ Reaction conditions were identified that led to complete hydrolysis of the anhydrides (either HBr at 80 °C or ZnCl_2_ and HCl at 37 °C). After optimization, the anhydrides in the crude mixture of 1-Fm-InsP_6_ derivatives obtained above were hydrolysed and the reaction progress was monitored by ^31^P-NMR (see ESI Fig. 5†). Upon disappearance of all pyrophosphate signals, the reaction mixture was diluted with water, neutralized with NH_4_HCO_3_ (pH = 8.0, 1 M) and purified by SAX on a Q-sepharose column using a NaClO_4_ (1 M) gradient as eluent.

CE-MS analysis of the material before purification revealed formation of 1-Fm-InsP_6_8 as main product (Scheme 3c), while InsP_6_ and InsP_5_ were the sole side products and readily removable by SAX as shown by CE-MS analysis after purification. The position of the Fm group was initially verified *via* 2D-NMR spectroscopy (see ESI Fig. 7 and 8†). Overall, in the whole telescoping sequence, 1-Fm-InsP_6_8 was synthesized in 45% yield directly from 1-OH-InsP_5_ (Scheme 3d). To demonstrate further utility of this method, we used the sequence to introduce a 7-(diethylamino)-4-(hydroxymethyl)-coumarin (in the following DEACM) moiety at the phosphate in the 1-position. Thus, photocaged 1-DEACM-InsP_6_11 was synthesized, simply by exchanging the cyclization prone P-amidite (Scheme 3d).

We next studied the dephosphorylation of the Fm modified InsP_6_8 using the promiscuous commercial phytase Natuphos as a very mild and potentially selective alternative to acidic hydrolysis.^35,36^ Its action on InsP_6_ led to quantitative dephosphorylation to 2-InsP_1_ within 10 min (see ESI Table 6†).^49^ Importantly, this is also an interesting starting material for the introduction of further modifications. Isomer identity was assigned *via* NMR in accordance with literature.^62^

**Scheme 4:**
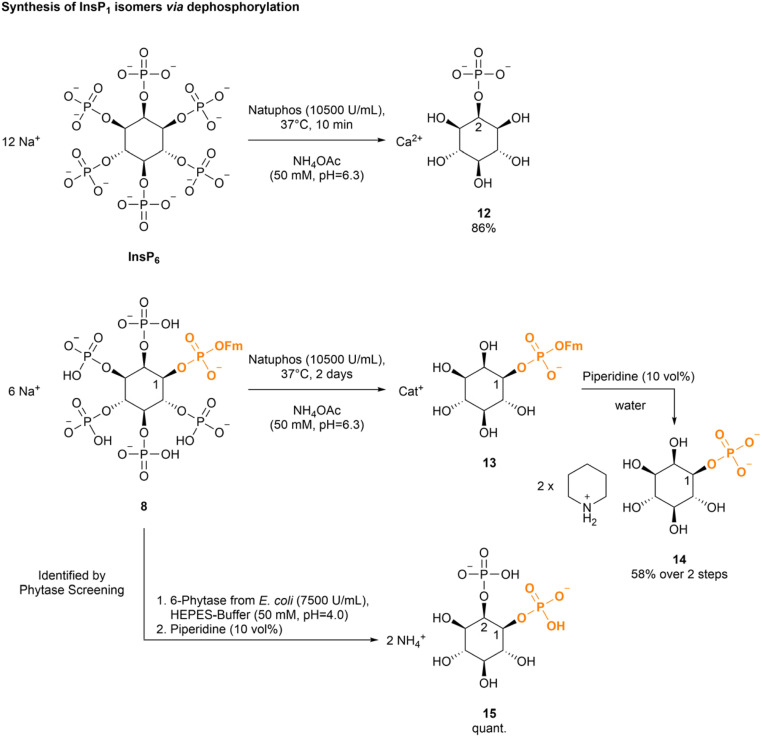
Dephosphorylation of InsP_6_ derivatives with Natuphos (10500 U mL^−1^, 3150 U was used for the digest of phytate, 2100 U was used for the digest of 8) in NH_4_OAc (50 mM, pH 6.3) leads to defined InsP_1_ isomers. 1,2-InsP_2_ was obtained as dephosphorylation product of 1-Fm-InsP_6_8 using the 6-Phytase from *E. coli* (7500 U mL^−1^, 1 U phytase was used) in HEPES-buffer (50 mM HEPES, 10 mM NaCl, 5% glycerol, 2 mM DTT, 0.5 mM, MgCl_2_, pH = 4.0; at 28 °C for 45 min) and subsequent basic deprotection.

Phytases initiate the stepwise dephosphorylation of phytate with a high stereo- and regioselectivity.^63^ 2-InsP_1_ with the phosphate in axial position is the endpoint of many naturally occurring phytases,^42–48^ but phytases stopping at different InsP derivatives (*e.g.* InsP_5_, InsP_3_) are known.^42,63–66^ However, unlike XopH these enzymes often deliver mixtures of *myo*-inositol phosphate intermediates. The dephosphorylation of InsP_6_ to 2-InsP_1_ by Natuphos produces 5 equivalents of phosphate (P_i_), which were removed by precipitation with Ba(OAc)_2_ ^48^ or CaCl_2_. The product was then obtained by precipitation from EtOH. Following this approach, 1 g of phytate was digested to 2-InsP_1_ in 86% yield (Scheme 4).^48^ Next, the digest of chiral 1-Fm-InsP_6_8 by Natuphos was monitored using ^31^P-NMR (see ESI Fig. 10†) and a slower reaction compared to unprotected phytate was observed (>24 h for complete conversion). However, a complete digestion of the phosphate monoesters to deliver phosphate diester 1-Fm-InsP_1_13 was achieved after 2 days of incubation. The product 13 was purified by reversed-phase medium pressure liquid chromatography (RP-MPLC) to remove excess P_i_. Subsequent basic deprotection led to 1-InsP_1_14 in 58% over two steps, starting from enantiomerically pure 1-Fm-InsP_6_8.

With 1-InsP_1_ in hand, enantiomer identity of the product was further corroborated using optical rotation and comparison to an original standard.^20,26,67^ This additionally affirms our assignment, that the 1-position in 1-OH-InsP_5_ is modified in our transient phosphitylation approach. Depending on the reaction time (<48 h), we observed transient accumulation of an InsP_2_ species resulting from incomplete dephosphorylation. Based on the selectivity of Natuphos described above, we tentatively assigned the isomer as 1-Fm-1,2-InsP_2_. Indeed, basic deprotection of the Fm group with piperidine after purification led to formation of enantiopure 1,2-InsP_2_ validated by CE-MS experiments and spiking with defined InsP_2_ isomers (see ESI Fig. 11†).^9^ In summary, the synthesis of enantiopure 1-InsP_1_ from InsP_6_ using a chemoenzymatic approach is possible in 3 steps.

**Scheme 5:**
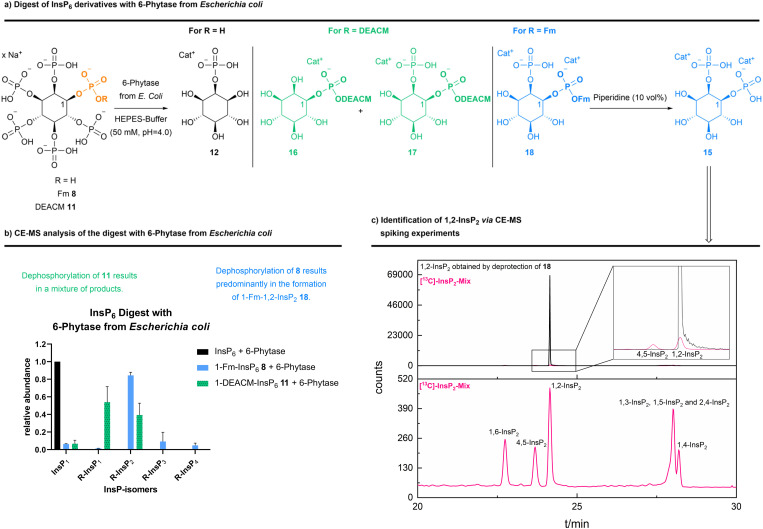
(a) Different InsP_6_ (15 mM) were dephosphorylated with 6-phytase from *Escherichia coli* (7500 U mL^−1^ at pH = 5.0, 1 U phytase was used) (used buffer: 50 mM HEPES, 10 mM NaCl, 5% glycerol, 2 mM DTT, 0.5 mM, MgCl_2_, pH = 4.0; at 28 °C for 45 min). CE-qTOF-MS (BGE: NH_4_OAc 35 mM pH = 9.7, CE voltage: 30 kV, CE current: 23 μA, injection: 100 mbar, 15 s (30 nL)). (b) Analysis revealed different InsPs as product mixtures, depending on the used InsP_6_ derivative. 1-Fm-InsP_6_8 was relatively cleanly dephosphorylated to an InsP_2_ derivative. (c) The formed 1,2-InsP_2_ was identified *via* CE-QQQ-MS (BGE: NH_4_OAc 35 mM pH = 9.7, CE voltage: 30 kV, CE current: 23 μA, injection: 100 mbar, 10 s (20 nL)) spiking experiments^9^ using a [^13^C]-InsP_2_ mix (obtained *via* decomposition of [^13^C]-InsP_6_ at 100 °C), after basic deprotection (piperidine (10 vol%)) of 1-Fm-InsP_2_18.

Next, we screened a small panel of phytases to potentially obtain other phosphorylated inositol isomers or a higher accumulation of defined intermediates. As starting materials, either InsP_6_, 1-DEACM-InsP_6_11 or 1-Fm-InsP_6_8 were studied. The resulting InsP mixtures were analyzed by CE-MS (see Scheme 5 and ESI Fig. 13†). All tested phytases hydrolyzed InsP_6_ to 2-InsP_1_, as confirmed by CE-MS spiking experiments with [^18^O]-2-InsP_1_ (see ESI Fig. 14†). 1-DEACM-InsP_6_11 was hydrolyzed by all tested phytases to a mixture of 1-DEACM-InsP_1_ and 1-DEACM-InsP_2_, which is not useful from a preparative perspective. However, 1-Fm-InsP_6_8 was predominantly (>80%) dephosphorylated under comparable conditions to 1-Fm-1,2-InsP_2_. Again, isomer assignment was achieved after basic deprotection and subsequent CE-MS spiking experiments (Scheme 5 and ESI Fig. 12†).^9^ Decomposition of [^13^C]-labeled InsP_6_ (by incubation at 100 °C) led to a mixture of all InsP_2_ isomers, which was used in the following for spiking experiments, to verify the initial assignment (see Scheme 5).^9^

Glycerophosphoinositols (hereafter referred to as GroPIns) are produced *in vivo* by phospholipase A_2_ cleavage of the glycerol-phosphate-diester in PtdInsP's. These metabolites are active as cellular signals.^68–70^ Previous syntheses of GroPIns were for example achieved *via* saponification of PtdIns.^71^

**Scheme 6:**
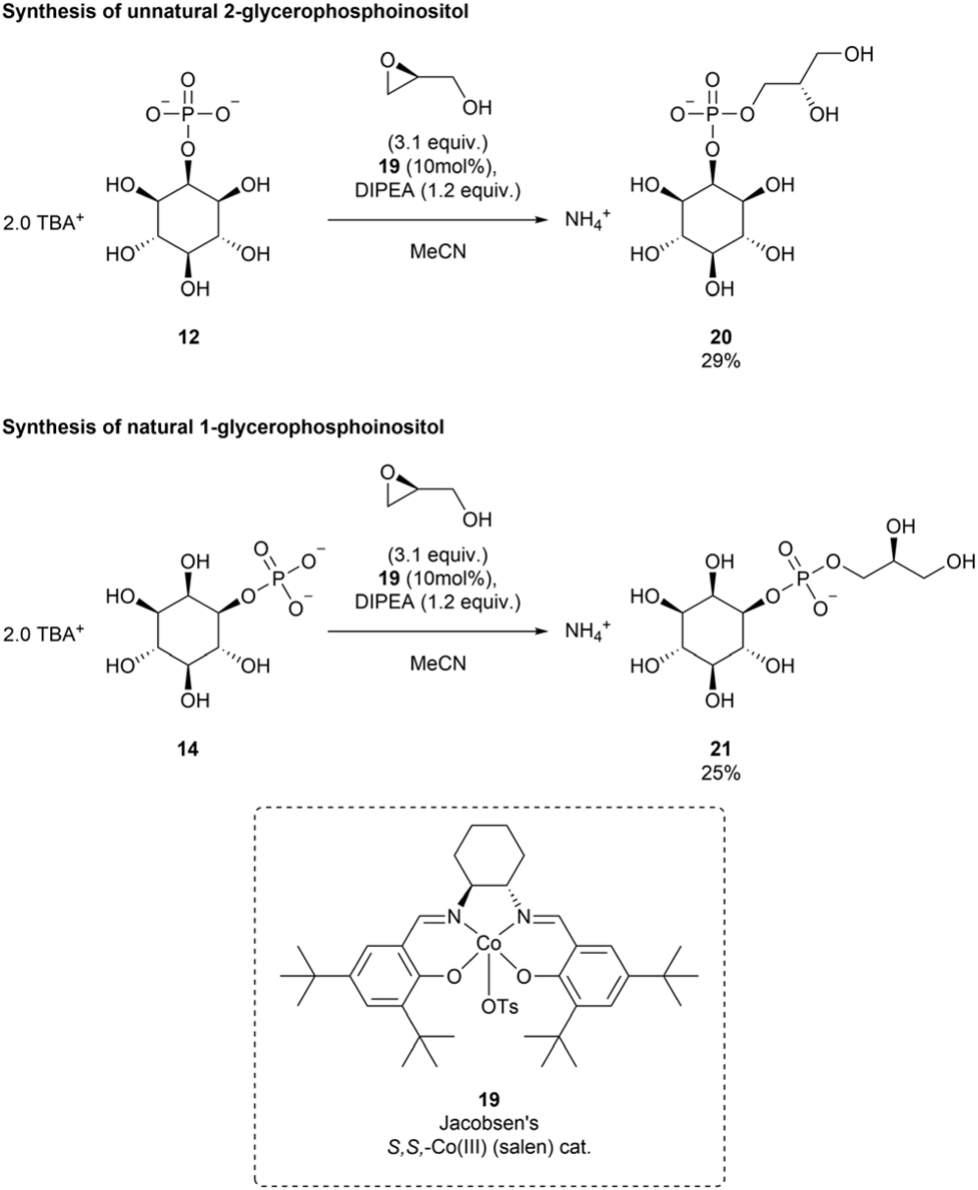
Synthesis of 1-GroPIns 21 and the non-natural 2-GroPIns derivative 20*via* Co(iii) catalyzed epoxide ring opening of *S*-(−)-glycidol with InsP_1_ isomers.

We envisioned synthetic access to GroPIns and non-natural derivatives using the unprotected InsP_1_ isomers obtained in this study. Minnard recently demonstrated the catalytic ring-opening of epoxides with phosphate diester nucleophiles using Co(salen) complexes.^72^ Herein, we extend this approach to InsP_1_ TBA salts as phosphate monoester nucleophiles. We started our exploration of suitable reaction conditions (see ESI Table 7 and Fig. 15†) with 2-InsP_1_12 since it is readily available from InsP_6_. (*S*)-glycidol was used as electrophile. Jacobsen's Co(iii) salen catalyst^73^ was added to promote ring opening of the epoxide (Scheme 6).

The reactions did not go to completion. No improvements were observed neither for longer reaction times nor for additional epoxide additions, and the crude reaction mixture was directly purified *via* SAX after an overnight reaction. This allowed isolation of the desired GroPIns derivatives and re-isolation of unreacted InsP_1_ starting material. The optimized reaction conditions gave access to 2-GroPIns 20 in 29% isolated yield from unprotected 2-InsP_1_12. Natural 1-GroPIns 21 was then obtained under analogous conditions in 25% yield. These isolated yields are comparatively low, however, this is compensated by re-isolation of the starting material (up to 65%). Of note, the required enantiopure 1-InsP_1_ is now available on larger scale using the XopH dephosphorylation approach described in this paper.

## Conclusion

The stereoselective synthesis of inositol phosphates and their derivatives is usually based on desymmetrization of *myo*-inositol. However, dephosphorylation of abundant *meso* InsP_6_ as a complementary approach has rarely been studied and was abandoned despite promising studies in the 1990s.^35,36^ Here, we demonstrate the synthetic potential of selective enzymatic dephosphorylations as a tool to access defined InsP_*x*_ isomers and their transformation into otherwise difficult to access derivatives.

After enzymatic desymmetrization using XopH, 1-OH-InsP_5_ is converted into phosphate diesters attached selectively to the 1-position using a transient phosphitylation approach with a cyclization prone P-amidite. Acidic ring opening of the ensuing cyclic pyrophosphate esters achieves convergence of complex mixtures into a single product.

This methodology unlocks new synthetic pathways, allowing hydroxyl group phosphorylation even in the presence of unprotected phosphates. The obtained InsP_6_ derivatives were subjected to dephosphorylation by different phytases. With this approach, it is possible to obtain enantiopure 1-InsP_1_, 1,2-InsP_2_ and other derivatives. To further demonstrate the versatility of the obtained building blocks, other metabolites (2-InsP_1_,^8^ 1-GroPIns^68,70^ and 2-GroPIns) were synthesized using Co(iii) catalyzed glycidol epoxide openings.

Selective dephosphorylation of InsP_6_ can be considered as an alternative desymmetrization route compared to classical approaches starting from inositol. One can imagine an even wider scope of selective dephosphorylations with appropriate phytases that have the potential to transform the way of how we generally think about synthesizing InsPs. Our paper provides a new entry point into the world of phosphorylated metabolites using phosphates as key strategic components that are still too “seldom used as intermediates by organic chemists”.^1^

## Author contributions

G. M. H. and H. J. designed the molecules. G. M. H. synthesized and analyzed the compounds. G. L. helped with compound characterization by CE-MS. G. M. H. drafted the initial manuscript and prepared the figures. K. D. and R. G. provided expertise regarding phytases and provided different phytases for experiments. E. L. and G. S. provided XopH digests and enzymes and helped with scaling-up and the phyatese screening. N. J. helped with purification of molecules and isomer assignment. H. J. conceived the project. All authors provided input on the final version of the paper.

## Conflicts of interest

There are no conflicts to declare.

## Supplementary Material

SC-016-D5SC02844B-s001

## Data Availability

Experimental procedures, characterization data and data supporting this article have been included as part of the electronic (ESI).†
