# Supplementary material for: Stereoselective chemoenzymatic phytate transformations provide access to diverse inositol phosphate derivatives
Source: Chem Sci. 2025 Jun 23;16(29):13459–67. doi: 10.1039/d5sc02844b (PMC12203238; doi:10.1039/d5sc02844b)
Supplement: SC-016-D5SC02844B-s001 [file SC-016-D5SC02844B-s001.pdf]

Supplementary Information (SI) for Chemical Science.
This journal is © The Royal Society of Chemistry 2025

Stereoselective chemoenzymatic phytate transformations provide access to diverse inositol phosphate derivatives

Georg Markus Häner[a], Guizhen Liu[a], Esther Lange[b], Nikolaus Jork[a], Klaus Ditrich[c], Ralf Greiner[d], Gabriel Schaaf[b], Henning J. Jessen[a*]

Supporting Information

Contents

1. General Informations ... 3
1.1. Capillary Electrophoresis ... 4
2. Experimental ... 6
2.1. Phytase dephosphorylation assay ... 6
2.1.1. Natuphos dephosphorylations ... 6
2.2. Tetrabutylammonium salt formation ... 6
2.3. Synthesis of 1-OH-InsP$_5$ 2 ... 7
2.4. Salt metathesis of 1-OH-InsP$_5$ 2 ... 7
2.5. Synthesis of 1,1-dichloro-*N,N*-dicyclohexylphosphanamine 22 ... 8
2.6. Synthesis of bis((9*H*-fluoren-9-yl)methyl) dicyclohexylphosphoramidite 1 ... 8
2.7. Synthesis of *N,N,N′,N′*-tetraisopropyl-1-phenoxyphosphanediamine 23 ... 9
2.8. Synthesis of (9*H*-fluoren-9-yl)methyl phenyl diisopropylphosphoramidite 9 ... 10
2.9. Synthesis of (diethylamino)-4-(hydroxymethyl)-2*H*-chromen-2-one 24 ... 11
2.10. Synthesis of (7-(diethylamino)-2-oxo-2*H*-chromen-4-yl)methyl phenyl diisopropylphosphoramidite 10 ... 11
2.11. Synthesis of Inositoltrispyrophosphate 25 ... 12
2.12. Synthesis of 1-Fm-InsP$_6$ 8 ... 13
2.13. Synthesis of 1-DEACM-InsP$_6$ 11 ... 14
2.14. Synthesis of 2-InsP$_1$ 12 ... 15
2.15. Synthesis of 1-Fm-InsP$_1$ 13 ... 16
2.16. Synthesis of 1-InsP$_1$ 14 ... 17
2.17. Synthesis of 1,2-InsP$_2$ 15 ... 17
2.18. Synthesis of Co(III) catalyst 19 ... 18
2.19. Synthesis of 2-glycerophosphoinositol 20 ... 19
2.20. Synthesis of 1-glycerophosphoinositol 21 ... 19
3. Transient Phosphitylation monitored by ^{31}P-NMR ... 21
3.1. Transient Phosphitylation of ADP ... 21
3.2. Transient Phosphitylation of 1-OH-InsP$_5$ with P-amidite 1 ... 22
3.3. Transient Phosphitylation of 1-OH-InsP$_5$ with P-amidite 9 ... 23
4. Towards the linearization of cyclic inositolpyrophosphates to InsP$_6$ derivatives ... 24
4.1. Screening for conditions to ring open the cyclic pyrophosphates of inositoltrispyrophosphate (ITPP) ... 24
4.1.1. Attempted nucleophilic ring-opening of ITPP with amines ... 24
4.1.2. Towards hydrolysis of ITPP to InsP$_6$ ... 25
4.2. NMR monitoring of the hydrolysis of ITPP ... 26
4.3. ^{31}P-NMR monitoring of the hydrolysis of 1-Fm-InsP$_6$ 8 ... 27
4.4. ^{31}P-NMR monitoring of the hydrolysis of 1-DEACM-InsP6 11 ... 28
4.5. 2D-NMR of 1-Fm-InsP$_6$ 8 ... 29
5. Natuphos digest of InsP$_6$ ... 31
5.1. Purification of 2-InsP$_1$ *via* precipitation monitored *via* ^{31}P-NMR monitoring ... 32
5.2. Natuphos digest of 1-Fm-InsP$_6$ 8 monitored *via* ^{31}P-NMR ... 33
6. Identification of the formed InsP$_2$ isomers obtained by dephosphorylation of 1-Fm-InsP$_6$ 8 ... 34
6.1. by Natuphos – spiking experiments with InsP$_2$ standards ... 34
6.2. by 6-Phytase from *Escherichia coli (7500 U/mL)* – spiking experiments with InsP$_2$ standards ... 35
7. Phytase Screen ... 36
7.1. Identification of the formed InsP$_1$ *via* spiking experiments ... 37
8. Co(III) catalysis screening ... 38
8.1. NMR monitoring for extend reaction times ... 39
9. Literature ... 40
10. Capillary Electrophoresis Integration ... 41
10.1. Blank ... 41
10.2. Dephosphorylation of phytate with 6-phytase from *Citrobacter braakii* (30000 U/mL) ... 41
10.3. Dephosphorylation of phytate with 6-phytase from *Escherichia coli* (7500 U/mL) ... 42
10.4. Dephosphorylation of phytate with 6-phytase from *Escherichia coli* (5000 U/mL) ... 42
10.5. Dephosphorylation of phytate with 3-phytase from *Aspergillus niger* (10000 U/mL) ... 43
11. NMR Spectra ... 43

1. General Informations

All solvents and reagents (analytical grade) were obtained from commercial sources (VWR/Merck, Alfa Aeser, ChemPur, Roth, TCI, Acros, Sigma Aldrich) and used without further purifications, if not stated otherwise.

Dry solvents were either obtained from commercial sources (VWR/Merck, Alfa Aeser, ChemPur, Roth, TCI, Acros, Sigma Aldrich) or purified on a *Braun Solvent Purification System 800* and stored over activated molecular sieves (3Å) und an argon atmosphere.

Flame dried glass ware was used for all reactions. If moisture sensitive reagents were applied, the reactions were conducted under an atmosphere of Argon.

Strong Anion Exchange (SAX) chromatography was performed on an ÄKTA™ pure system. Samples were eluted from the solid phase (Q-sepharose fast flow (Cytiva) columns) using a gradient of $NaClO_4$ (1 M), NH_4HCO_3 (1 M, pH = 8) or NH_4COOH (1 M, pH = 7).

A PuriFlash® 5.125 (Interchim®) was used for reverse phase medium pressure liquid chromatography (RP-MPLC), using prepacked columns, obtained from Interchim®, as stationary phase.

Christ Alpha 1-4 LDplus or *Alpha 1-2 LDplus* freeze drier were used for lyophilisation of samples. *Bruker Avance III HD 300 MHz*, *Avance 400 MHz* or *Avance III HD 500 MHz* spectrometers were used measure nuclear magnetic resonante (NMR) spectra. Deuterated solvents were purchased from commercial suppliers (Eurisotop, Deutero), used without further purifications and stored over activated molecular sieves (3Å). *MestreNova 12.01* was used to analyse the resulting spectra, referenced to the respective deuterated solvent peaks (for ^{1}H-NMR: $CDCl_3$: $\delta = 7.26$ ppm, DMSO-d_6: $\delta = 2.50$, MeCN-d_3: $\delta = 1.94$, DMF-d_7: $\delta = 2.92$ ppm, D_2O: $\delta = 4.79$ ppm; for ^{31}C-NMR: $CDCl_3$: $\delta = 77.16$ ppm, DMSO-d_6: $\delta = 39.52$, MeCN-d_3: $\delta = 1.32$ ppm, DMF-d_7: $\delta = 34.9$ ppm.).[1]

1.1. Capillary Electrophoresis

Capillary electrophoresis (CE) was measured on a fused silica capillary (100 cm, 50 µm, internal diameter and 365 µm outer diameter) on an *Agilent 7100* capillary electrophoresis system coupled to an *Agilent 6520* Q-TOF- or *Agilent 6495C* QQQ-mass spectrometer, equipped with an *Agilent Jet Stream* electrospray ionization source. Data acquisition was performed with *Agilent OpenLAB CDS Chemstation 2.3.53*, the resulting data was analyzed with *Agilent MassHunter Workstation Version B.08.00*. The capillary was flushed with NaOH (1 N, 10 min) and water (10 min) for equilibration. The capillary was equilibrated with back ground electrolyte (BGE) (10 min) prior to every measurement. The following BGE were used: NH_4OAc (35 mM, pH = 9.9, titrated with NH_3) and NH_4OAc (40 mM, pH = 9.1, titrated with NH_3). Isopropanol:water (1:1), spiked with mass references (TFA anion, $[M\text{-}H]^-$: 1112.9855 and HP-0921, $[M\text{-}H\text{+}CH_3COOH]^-$: 980.0163) for qTOF measurements, was deployed as sheath liquid using an isocratic pump with constant flow rate (1.5 µL/min).

Samples were applied *via* pressure (100 mbar, 10-15 s, 20-30 µL), followed by BGE injection (50 mbar, 2 s) and voltage (30 kV) was applied over the capillary to establish a constant current (23 µA). The following source parameters (Supplementary Table 1) were used for measurements in negative ionization mode.

Analytes were identified based on exact *m/z* ratios, for qTOF measurements and based on characteristic fragmentation patterns (Supplementary Table 2), for QQQ measurements.

Supplementary Table 1 ESI source parameters.

	q-TOF	QQQ
**Capillary Voltage**	-3000 V	-2000 V
**Fragmentor**	140 V	166 V
**Drying Gas Temperature**	250°C	150°C
**Drying Gas Flow**	8 L/min	11 L/min
**Nebulizer**	8 psi	8 psi

Supplementary Table 2 Optimized fragmentation source parameters, used for CE-QQQ-MS measurements.

Analyte	Precursor Ion	Product Ion	dwell	Fragmentor (V)	Collision Energy (V)	Cell Accelerator Voltage	Polarity
$InsP_1$	259	79.1	50	166	41	4	Negative
$[^{18}O]$-1-$InsP_1$	263	83	50	166	17	1	Negative
$[^{18}O]$-2-$InsP_1$	263	83	50	166	17	1	Negative
$[^{18}O]$-5-$InsP_1$	263	83	50	166	17	1	Negative
$[^{13}C_6]$-$InsP_1$	265	79.1	50	166	41	4	Negative
$InsP_2$	339	241	50	166	21	4	Negative
$[^{13}C_6]$-$InsP_2$	345	247	50	166	21	4	Negative
$InsP_3$	418.9	320.8	50	166	17	4	Negative
$[^{13}C_6]$-$InsP_3$	424.9	326.8	50	166	17	4	Negative
$InsP_4$	249	418.9	50	166	5	1	Negative
$[^{13}C_6]$-$InsP_4$	252	424.9	50	166	5	1	Negative
$InsP_5$	289	498.9	50	166	9	3	Negative
$[^{13}C_6]$-$InsP_5$	292	504.9	50	166	9	3	Negative
$InsP_6$	328.9	480.9	50	166	13	4	Negative
$[^{13}C_6]$-$InsP_6$	331.9	486.9	50	166	13	4	Negative
$[^{18}O_{12}]$-$InsP_6$	340.9	494.9	50	166	13	4	Negative
$InsP_7$	368.9	319.9	50	166	9	3	Negative
$[^{13}C_6]$-$InsP_7$	371.9	322.9	50	166	9	3	Negative
$InsP_8$	408.9	359.8	50	166	9	1	Negative
$[^{13}C_6]$-$InsP_8$	411.9	362.8	50	166	9	1	Negative

2. Experimental

2.1. Phytase dephosphorylation assay

The phytase assay was performed by incubating the respecting $InsP_6$ derivatives (15 mM, 0.66 µL) and 1U enzyme in buffer (HEPES 50 mM, NaCl, 10 mM, glycerol 5%, DTT 2 mM, $MgCl_2$ 0.5 mM, pH = 4, total reaction volume 15 µL) at 28°C for 45 min and the reaction outcome was analysed by CE-MS.

Supplementary Table 3 Screened phytases. The activity was expressed as U = µmol phosphate released per min. All phytases are commercially available and purified prior to use.

Entry	Phytase	Origin	optimal pH	Activity
1	6-phytase	*Citrobakter braaki*	2.5 to 5.5	30000 U/mL
2	6-phytase	*Escherichia coli*	3 to 6	7500 U/mL
3	6-phytase	*Escherichia coli*	2.5 to 6	5000 U/mL
4	3-phytase	*Aspergillus niger*	4 to 6	10000 U/mL

2.1.1. Natuphos dephosphorylations

$InsP_6$ derivatives were dissolved in NH_4OAc (pH = 6.3), Natuphos (kindly provided by BASF, Art.Nr.: 52587753, Lot.Nr.: 52913677L0, 10500 U/mL at pH = 6.3 with U = µmol phosphate released per minute) was added and the reaction mixture was incubated at 37°C. The reaction was monitored by $^{31}P\{^1H\}$-NMR and the reaction mixture was precipitated in EtOH upon completion.

2.2. Tetrabutylammonium salt formation

To exchange the cations of the here used phosphates, a solution of the respective anion was passed through a column loaded with activated Dowex® 50WX8 H^+ form, a solution of tetrabutylammonium hydroxide (TBA-OH) was added to the eluate and the solution was lyophilized to obtain the resulting TBA salt. The solid residue was equilibrated before and after use with aq. HCl (1 M) and washed with miliQ water until neutral subsequently.

2.3. Synthesis of 1-OH-InsP5 2

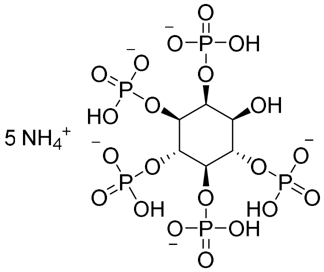

2

Chemical Formula: $C_6H_{12}O_{21}P_5^{5-}$

Molecular Weight: 575.0126

The procedure was adapted from literature:[2]

InsP6 (10 g, 8.95 mmol) in NH_4OAc (800 mL, 150 mM, pH = 7) was incubated in the presence of XopH (0.13 µg/mL, 14*10^{-6} U/µg with U = µmol phosphate released per minute[2]) at 28°C for 8 h. The enzyme was deactivated by incubation at 95°C for 15 min and portioned for storage.

A portion (50 mL) was diluted with water (4 × 50 mL), applied onto a Q-sepharose columne (*XK26*) and eluted using a NH_4HCO_3 (1 M, pH = 8) gradient. The fractions were analyzed by ^{31}P-NMR and product containing fractions were lyophilized thrice to obtain 1-OH-InsP5 (255 mg, 383 µmol, 69% for the pentaammonium salt) as a white solid.

Analytical data is in accordance with literature.[2]

2.4. Salt metathesis of 1-OH-InsP5 2

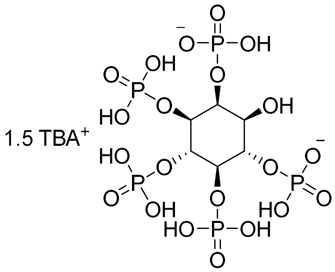

2

Chemical Formula: $C_6H_{12}O_{21}P_5^{5-}$

Molecular Weight: 575.0126

The salt metathesis was performed according to the general procedure (see chapter 2.2).

1-OH-InsP5 (255 mg, 383 µmol, 1.0 equiv.) was dissolved in water (5 mL), and applied to an activated Dowex® 50WX8 H^+ column. The column was eluted with water until neutral, TBA-OH (20wt% in water, 0.75 mL, 150 mg, 578 µmol, 1.5 equiv.) was added and the solution was lyophilized to obtain a white solid (300 mg, 316 µmol, 82% for 1.5 equiv. TBA^+).

^{1}H NMR (400 MHz, Deuterium Oxide) δ = 4.70 (dt, *J* = 9.2, 2.6 Hz, 1H), 4.41 (q, *J* = 9.5 Hz, 1H), 4.33 – 4.14 (m, 3H), 3.75 (dt, *J* = 9.8, 2.2 Hz, 1H), 3.09 – 2.92 (m, 12H), 1.61 – 1.38 (m, 12H), 1.20 (h, *J* = 7.4 Hz, 12H), 0.78 (t, *J* = 7.4 Hz, 18H) ppm.
^{31}P NMR (162 MHz, Deuterium Oxide) δ = 0.09 (d, *J* = 9.2 Hz), -0.16 (d, *J* = 9.3 Hz), -0.61 (d, *J* = 9.2 Hz), -0.89 (d, *J* = 9.0 Hz) ppm.

2.5. Synthesis of 1,1-dichloro-*N,N*-dicyclohexylphosphanamine 22

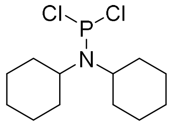

22
Chemical Formula: $C_{12}H_{22}Cl_2NP$
Molecular Weight: 282.1888

Distilled PCl_3 (5.0 mL, 7.9 g, 58 mmol, 1.0 equiv.) was dissolved in Et_2O (100 mL) in a sulphuration flask and cooled to 0°C. Dicyclohexylamine (23.0 mL, 20.9 g, 115 mmol, 2.0 equiv.) in Et_2O (150 mL) was added dropwise. The reaction mixture was stirred for 2 h and then filtered over neutral Al_2O_3. The solution was concentrated to 80 mL under reduced pressure and incubated at -20°C for 2 days. The formed crystals were filtered, the supernatant was concentrated *in vacuo* and crystalized and the target compound was obtained as colorless crystals (12.0 g, 42.8 mmol, 78%).
^{1}H NMR (300 MHz, Chloroform-*d*): δ = 3.52-3.35 (m, 2H), 1.94-0.95 (m, 20H) ppm.
^{13}C NMR (101 MHz, Chloroform-*d*): δ = 29.44, 26.34, 25.48, 24.96 ppm.
^{31}P{^{1}H} NMR (122 MHz, Chloroform-*d*): δ = 169.65 ppm.

2.6. Synthesis of bis((9*H*-fluoren-9-yl)methyl) dicyclohexylphosphoramidite 1

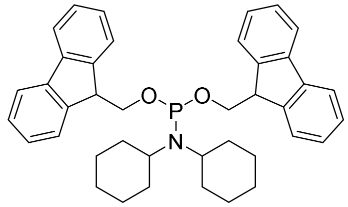

1
Chemical Formula: $C_{40}H_{44}NO_2P$
Molecular Weight: 601.7708

9-Fluroenylmethanol (5.25 g, 16.8 mmol, 1.0 equiv.) was dried in a desiccator, dissolved in THF (150 mL), NEt_3 (5.46 mL, 3.97 g, 39.2 mmol, 2.1 equiv.) was added and the mixture was stirred at 0°C for 30 min. 1,1-dichloro-*N,N*-dicyclohexylphosphanamine **22** (5.25 g, 16.8 mmol, 1.0 equiv.)

was added, the mixture was stirred at 0°C for 30 min and allowed to reach RT overnight. The reaction mixture was filtered over neutral Al_2O_3 and the solvent was removed under reduced pressure. The crude product was purified by flash column chromatography on SiO_2 (cyclohexane:Et_2O, 9:1) to obtain **1** as a colourless sticky solid (9.78 g, 15.5 mmol, 92%).

^{1}H NMR (300 MHz, Chloroform-*d*): δ = 7.73-7.63 (m, 4H), 7.63-7.52 (m, 4H), 7.31 (qd, *J* = 7.5, 1.2 Hz, 4H), 7.26-7.20 (m, 4H), 4.12 (t, *J* = 7.0 Hz, 2H), 3.95 (dt, *J* = 9.8, 6.7 Hz, 2H), 3.75 (dt, *J* = 9.9, 7.2 Hz, 2H), 3.15-2.94 (m, 2H), 1.71-1.47 (m, 10H), 1.37 (ddd, *J* = 15.3, 12.6, 6.5 Hz, 4H), 1.29-1.07 (m, 4 H), 0.97 (qt, *J* = 12.8, 3.3 Hz, 2H) ppm.

^{13}C NMR (101 MHz, Chloroform-*d*): δ = 144.97 (d, *J* = 29.6 Hz), 141.37, 127.49, 127.45, 126.94 (d, *J* = 1.9 Hz), 125.61, 125.33, 119.95, 119.88, 68.12, 66.07 (d, *J* = 17.5 Hz), 52.31 (d, *J* = 10.2 Hz), 49.31 (d, *J* = 8.0 Hz), 35.68 (d, *J* = 7.3), 26.81, 25.78, 25.76 ppm

^{31}P{^{1}H} NMR (122 MHz, Chloroform-*d*): δ = 146.55 ppm.

HRMS (ESI) for $C_{40}H_{44}O_2NP$ $[M+H]^+$: calculated 602.3182, found 602.3180.

2.7. Synthesis of *N,N,N',N'*-tetraisopropyl-1-phenoxyphosphanediamine 23

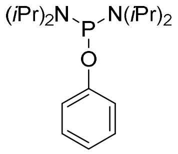

23

Chemical Formula: $C_{18}H_{33}N_2OP$

Molecular Weight: 324.4488

Phenol (1.68 g, 17.9 mmol, 1.0 equiv.) was dried under high vacuum for 2 h, dissolved in dry Et_2O (40 mL), NEt_3 (2.6 mL, 1.89 g, 18.6 mmol, 1.04 equiv.) was added, the reaction mixture was cooled to -20°C (ice/NaCl) and bis-(diisopropylamino)-chlorophosphine (4.79 g, 17.9 mmol, 1.0 equiv.) was added. The reaction mixture was diluted with Et_2O (50 mL) after 1.5 h and filtered over neutral Al_2O_3, the residue was washed with Et_2O (4 × 100 mL) and the solvent was removed under reduced pressure to obtain colorless crystals (5.43 g, 16.7 mmol, 94%, 97% purity).

^{1}H NMR (400 MHz, Chloroform-*d*): δ = 7.22 (dd, *J* = 8.6, 7.4 Hz, 2H), 7.04 (ddt, *J* = 7.8, 2.0, 1.1 Hz, 2H), 6.90 (tt, *J* = 7.3, 1.1 Hz, 1H), 3.61 (dp, *J* = 11.3, 6.8 Hz, 4H), 1.20 (d, *J* = 6.8 Hz, 12H), 1.15 (d, *J* = 6.7 Hz, 12H) ppm.

^{31}P NMR (162 MHz, Chloroform-*d*): δ = 120.11 (t, *J* = 11.5 Hz) ppm.

^{13}C NMR (101 MHz, Chloroform-*d*): δ = 156.19 (d, *J* = 10.1 Hz), 129.20, 120.72 (d, *J* = 1.5 Hz), 118.88 (d, *J* = 11.8 Hz), 45.01 (d, *J* = 12.5 Hz), 24.41 (d, *J* = 8.9 Hz), 24.05 (d, *J* = 5.4 Hz) ppm.

HRMS (ESI) for $C_{18}H_{33}N_2OP$ $[M+H]^+$: calculated 325.2404, found 325.2406.

2.8. Synthesis of (9*H*-fluoren-9-yl)methyl phenyl diisopropylphosphoramidite 9

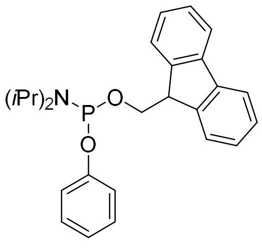

9

Chemical Formula: $C_{26}H_{30}NO_2P$

Molecular Weight: 419.5048

9-Fluorenylmethanol (1.23 g, 6.27 mmol, 1.0 equiv.) and **23** (2.04 g, 6.27 mmol, 1.0 equiv.) were dried *in vacuo* for 2 h. The reagents were dissolved in dry Et_2O (60 mL) and ETT (100 mg/mL in dry MeCN, 8.2 mL, 820 mg, 6.23 mmol, 1.0 equiv.) was added at 0°C. The reaction mixture was diluted with dry Et_2O (20 mL) after 19 h, filtered over neutral Al_2O_3, the stationary phase was washed with Et_2O (100 mL) and the solvents were removed under reduced pressure to obtain the crude as a colorless oil (2.26 g, 5.38 mmol, 86%, containing 10% unreacted starting material).

The crude was purified by DCVC on SiO_2 using a cyclohexan:EtOAc solvent mixture containing NEt_3 (5 %) and solvents were removed *in vacuo* to obtain a colorless oil (587 mg, 1.40 mmol, 22%).

^{1}H NMR (400 MHz, Chloroform-*d*): δ = 7.77 (dq, *J* = 7.6, 1.1 Hz, 2H), 7.69 (dq, *J* = 7.5, 0.9 Hz, 1H), 7.61 (dq, *J* = 7.5, 0.9 Hz, 1H), 7.40 (tddd, *J* = 7.5, 3.5, 1.2, 0.7 Hz, 2H), 7.34 – 7.23 (m, 4H), 7.10 – 7.04 (m, 2H), 7.04 – 6.99 (m, 1H), 4.28 – 4.22 (m, 1H), 4.11 (dt, *J* = 9.9, 6.7 Hz, 1H), 3.92 (dt, *J* = 9.9, 7.4 Hz, 1H), 3.78 (dp, *J* = 10.5, 6.8 Hz, 2H), 1.21 (d, *J* = 3.6 Hz, 6H), 1.20 (d, *J* = 3.6 Hz, 6H) ppm.

^{13}C NMR (101 MHz, Chloroform-*d*): δ = 154.86 (d, *J* = 7.4 Hz), 144.95, 144.58, 141.47 (d, *J* = 7.9 Hz), 129.48, 127.61, 127.58, 127.04, 126.99, 125.60, 125.39, 122.39 (d, *J* = 1.6 Hz), 120.31, 120.22, 119.98, 119.95, 66.61 (d, *J* = 17.2 Hz), 49.20 (d, *J* = 7.9 Hz), 43.73, 43.60, 24.78, 24.71, 24.63, 24.55 ppm.

^{31}P{^{1}H}-NMR (162 MHz, Chloroform-*d*): δ = 145.79 ppm.

HRMS (ESI) for $C_{26}H_{30}O_2NP$ $[M+H]^+$: calculated 420.2087, found 420.2084.

2.9. Synthesis of (diethylamino)-4-(hydroxymethyl)-2*H*-chromen-2-one 24

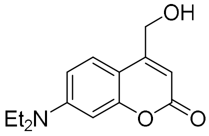

24
Chemical Formula: $C_{14}H_{17}NO_3$
Molecular Weight: 247.2940

20 was synthesized according to literature.[3]

2.10. Synthesis of (7-(diethylamino)-2-oxo-2*H*-chromen-4-yl)methyl phenyl diisopropylphosphoramidite 10

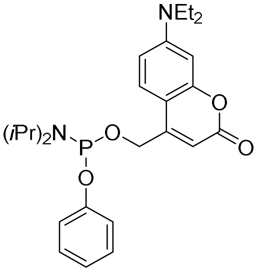

10
Chemical Formula: $C_{26}H_{35}N_2O_4P$
Molecular Weight: 470.5498

19 (349 mg, 1.08 mmol, 1.0 equiv.) and DEACM-OH **24** (267 mg, 1.08 mmol, 1.0 equiv.) were dried *in vacuo* for 4.5 h. The reagents were dissolved in dry Et_2O:CH_2Cl_2 (1:1, 30 mL) and ETT (100 mg/mL in dry MeCN, 1.4 mL, 1.08 mmol, 1.0 equiv.) was added at 0°C. The reaction mixture was diluted with Et_2O (50 mL) after 17.5 h, filtered over neutral Al_2O_3, the stationary phase was washed with Et_2O (100 mL) and the solvents were removed under reduced pressure to obtain a yellow oil (440 mg, 935 µmol, 87%).

^{1}H NMR (400 MHz, Chloroform-*d*): δ = 7.33 – 7.22 (m, 3H), 7.10 – 6.98 (m, 3H), 6.58 – 6.49 (m, 2H), 6.28 (t, *J* = 1.4 Hz, 1H), 4.86 (ddd, *J* = 7.6, 6.7, 1.4 Hz, 2H), 3.79 (dp, *J* = 10.7, 6.8 Hz, 2H), 3.40 (q, *J* = 7.1 Hz, 4H), 1.26 (d, *J* = 6.8 Hz, 6H), 1.20 (t, *J* = 7.1 Hz, 6H), 1.20 (d, *J* = 6.9 Hz, 6H).

^{13}C NMR (101 MHz, Chloroform-*d*) δ 162.41, 156.30, 154.54 (d, *J* = 7.2 Hz), 152.58 (d, *J* = 7.6 Hz), 150.58, 129.59, 124.53, 122.70 (d, *J* = 1.7 Hz), 120.17 (d, *J* = 8.9 Hz), 108.57, 106.55, 97.96, 62.05 (d, *J* = 18.7 Hz), 44.87, 43.93 (d, *J* = 12.9 Hz), 24.91 (d, *J* = 7.1 Hz), 24.53 (d, *J* = 7.2 Hz), 12.59 ppm.

^{31}P{^{1}H} NMR (162 MHz, Chloroform-*d*): δ = 147.30 ppm.

^{13}**C NMR** (101 MHz, Chloroform-*d*): δ = 162.41, 156.30, 154.54 (d, *J* = 7.2 Hz), 152.58 (d, *J* = 7.6 Hz), 150.58, 129.59, 124.53, 122.70 (d, *J* = 1.7 Hz), 120.17 (d, *J* = 8.9 Hz), 108.57, 106.55, 97.96, 62.05 (d, *J* = 18.7 Hz), 44.87, 43.93 (d, *J* = 12.9 Hz), 24.91 (d, *J* = 7.1 Hz), 24.53 (d, *J* = 7.2 Hz), 12.59 ppm.
HRMS (ESI) for $C_{26}H_{35}O_4N_2P$ $[M+Na]^+$: calculated 493.2227, found 493. 2232.

2.11. Synthesis of Inositoltrispyrophosphate 25

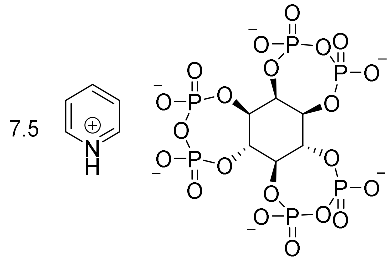

25
Chemical Formula: $C_6H_6O_{21}P_6^{6-}$
Molecular Weight: 599.9389

Based on literature procedures[4,5]:
Myo-inositolhexakisphosphate dodecasodium salt (1.07 g, 0.96 mmol, 1.0 equiv.) was passed through an activated DOWEX H^+ column, the eluate was adjusted to pH = 8 with pyridine and the solution was lyophilised. The obtained *myo*-inositolhexakisphosphate pyridinium salt was dissolved in water (10 mL) and added to a solution of dicyclohexylcarbodiimid (1.8 g, 8.72 mmol, 9.1 equiv.) in pyridine (30 mL) and heated to 70°C for 8 h. After stirring at RT overnight dicyclohexylcarbodiimide (750 mg, 3.63 mmol, 3.8 equiv.) was added and the reaction mixture was heated to 70°C for 10 h. After stirring at RT overnight dicyclohexylcarbodiimide (1.4 g, 6.79 mmol, 7.1 equiv.) was added and the reaction mixture was heated to 70°C for 8 h. The solvents were evaporated under reduced pressure, the resulting solid was suspended in water (20 mL) and filtered, the white residue was washed with water (4 × 20 mL) and the filtrate was lyophilized to obtained a white hygroscopic solid (988 mg, 0.82 mmol, 86%).
^{1}**H NMR** (400 MHz, Deuterium Oxide): δ = 8.87 – 8.76 (m, 15H), 8.65 (tt, *J* = 7.8, 1.4 Hz, 8H), 8.10 (t, *J* = 6.9 Hz, 15H), 5.21 – 5.11 (m, 1H), 4.70 (dt, *J* = 9.0, 4.3 Hz, 1H), 4.56 – 4.49 (m, 2H), 4.44 – 4.31 (m, 2H) ppm.
^{31}**P{^{1}H} NMR** (162 MHz, Deuterium Oxide): δ = -9.69 (d, J = 22.1 Hz), -10.75 – -11.14 (m), -11.26, -14.49 (d, J = 21.9 Hz) ppm.
HRMS (ESI) for $C_6H_{12}O_{21}P_6$ $[M-2H]^{2-}$: calculated 301.9075, found 301.9082.

2.12. Synthesis of 1-Fm-InsP$_6$ 8

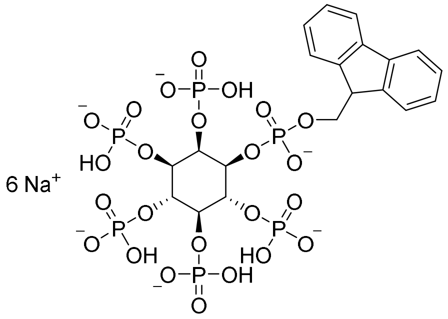

8

Chemical Formula: $C_{20}H_{22}O_{24}P_6^{6-}$

Molecular Weight: 832.2179

1-OH-InsP$_5$ × 1.15 tetrabutylammonium salt **2** (165 mg, 172 µmol, 1.0 equiv.) and **9** (579 mg, 1.38 mmol, 8.0 equiv.) were dissolved in dry MeCN (5.0 mL) and co-evaporated. ETT (300 mg, 2.31 mmol, 13.4 equiv.) was dissolved in dry MeCN (5.0 mL) and co-evaporated. The reagents were dissolved in dry DMF (16.0 mL), ETT was dissolved in dry MeCN (3.0 mL) and added to the reaction mixture at RT. Pyridinium Hydrobromide (244 mg, 1.53 mmol, 8.9 equiv.) was added after 22 h, *m*CPBA (70%wet, 235 mg, 953 µmol, 5.5 equiv.) was added at 0°C after 15 min and the reaction mixture was precipitated in ice cold Et_2O (8 vol.) in portions after 30 min. The solid residue was washed with acetone (30 mL) and dried under ambient conditions to obtain an off-white solid. The crude was dissolved in D_2O (5 mL), HBr (33% in acetic acid, 0.7 mL) was added and the reaction was heated at 80°C. The reaction mixture was diluted with water (30 mL) after 7 h, NH_4HCO_3 buffer (1 M, pH = 8, 5 mL) was added and the crude was purified by SAX on a Q-sepharose column (*XK16/20*) on a $NaClO_4$ (1 M) gradient. Product containing fractions were lyophilised, the residue was washed with acetone (2 × 20 mL), dissolved in water and lyophilised to obtain a white solid (75 mg, 77 µmol, 45% for the hexasodium salt).

NMR for NH_4^+ cations

^{1}H NMR (400 MHz, Deuterium Oxide): δ = 7.98 – 7.86 (m, 4H), 7.60 – 7.46 (m, 4H), 5.01 (d, J = 9.7 Hz, 1H), 4.58 – 4.35 (m, 5H), 4.23 (q, J = 9.5 Hz, 1H), 4.15 (t, J = 9.5 Hz, 1H), 4.05 (t, J = 9.4 Hz, 1H) ppm.

^{31}P NMR (162 MHz, Deuterium Oxide): δ = 1.75 (d, J = 9.9 Hz), 1.01 (d, J = 9.9 Hz), 0.36 (d, J = 9.6 Hz), -0.51 (d, J = 9.3 Hz), -0.75 (dd, J = 6.3 Hz) ppm.

^{13}C NMR (101 MHz, Deuterium Oxide): δ: = 144.35, 144.23, 140.96, 140.91, 127.95, 127.91, 127.56, 127.53, 125.81, 125.60, 120.04, 120.01, 77.30, 76.26, 75.17 (d, J = 7.5 Hz), 75.04 (d, J = 3.4 Hz), 73.36, 73.17, 67.54 (d, J = 5.9 Hz), 48.05 (d, J = 8.0 Hz) ppm.

NMR for Na^+ cations

^{1}H NMR (400 MHz, Deuterium Oxide): δ = 7.99 – 7.85 (m, 4H), 7.59 – 7.45 (m, 4H), 5.01 (t, J = 2.4 Hz, 1H), 4.55 (t, J = 9.4 Hz, 1H), 4.51 (t, J = 9.4 Hz, 1H), 4.58 – 4.38 (m, 3H), 4.25 (t, J = 9.4 Hz, 1H), 4.17 (dd, J = 9.8, 2.5 Hz, 1H), 4.07 (dd, J = 10.0, 2.3 Hz, 1H) ppm.

^{31}P NMR (162 MHz, Deuterium Oxide): δ = 1.80 (d, J = 9.7 Hz), 1.09 (d, J = 9.7 Hz), 0.42 (d, J = 9.5 Hz), 0.39 (d, J = 9.2 Hz), -0.37 – -0.76 (m) ppm.

^{13}C NMR (101 MHz, Deuterium Oxide): δ = 144.36, 144.26, 140.95, 140.89, 127.94, 127.89, 127.57, 127.53, 125.85, 125.63, 120.02, 119.99, 77.37, 76.19 (td, J = 6.2, 2.6 Hz), 75.21 (d, J = 6.5 Hz), 75.05 (dq, J = 6.4, 3.0 Hz), 73.46 (dd, J = 5.8, 2.8 Hz), 73.20 (dd, J = 6.1, 3.2 Hz), 67.55 (d, J = 5.7 Hz), 48.05 (d, J = 8.1 Hz) ppm.

HRMS (ESI) for $C_{20}H_{28}O_{24}P_6$ $[M-H]^-$: calculated 836.9323, found 836.9328.

$[\alpha]^{20}_{D}$ = 3.6 (0.25, water).

2.13. Synthesis of 1-DEACM-InsP$_6$ 11

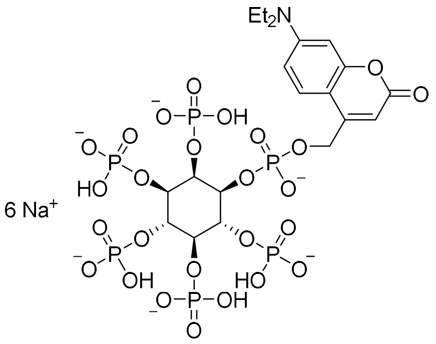

11

Chemical Formula: $C_{20}H_{27}NO_{26}P_6^{6-}$

Molecular Weight: 883.2629

1-OH-InsP$_5$ × 1.57 tetrabutylammonium salt **2** (23 mg, 24 µmol, 1.0 equiv.) was co-evaporated with dry MeCN (2 × 3 mL), the reagent was dissolved in dry DMF (1.0 mL) and **10** (88 mg, 187 µmol, 7.8 equiv.) and ETT (100 mg/mL in dry MeCN, 0.4 mL, 40 mg, 307 µmol, 12.8 equiv.) were added. Pyridinium hydrobromide (32 mg, 200 µmol, 8.3 quiv.) was added after 16 h, *m*CPBA (70%wet, 62 mg, 252 µmol, 10.5 equiv.) was added at 0°C after 45 min, the reaction mixture was precipitated in ice cold Et_2O (40 mL) after 30 min. The residue was dissolved in D_2O (1 mL), $ZnCl_2$ (114 mg, 837 µmol, 34.9 equiv.) and $HCl_{conc.}$ (0.35 mL, 154 mg, 4.24 mmol, 177 equiv.) were

added and the reaction mixture was incubated at 37°C for 19 h. The solution was diluted with water (20 mL) and NH_4HCO_3 buffer (1 M, 5 mL, pH = 8) and purified by SAX on a Q-sepharose column (*XK16/20*) on a $NaClO_4$ (1 M) gradient. Product containing fractions were lyophilised, washed with acetone (2 × 5 mL) and dried *in vacuo* to obtain a yellow solid (8 mg, 8 µmol, 35% for the hexasodium salt).

^{1}H NMR (400 MHz, D_2O): δ = 7.75 (d, *J* = 9.1 Hz, 1H), 6.92 (dd, *J* = 9.1, 2.4 Hz, 1H), 6.72 (d, *J* = 2.5 Hz, 1H), 6.37 (d, *J* = 1.4 Hz, 1H), 5.40 (d, *J* = 8.5 Hz, 2H), 5.08 (d, *J* = 9.5 Hz, 1H), 4.61 – 4.43 (m, 3H), 4.30 – 4.11 (m, 4H), 3.50 (q, *J* = 7.1 Hz, 6H), 1.22 (t, *J* = 7.1 Hz, 9H) ppm.

^{13}C NMR (101 MHz, D_2O): δ = 166.03, 155.66, 155.60, 155.55, 151.27, 125.88, 110.31, 106.18, 103.54, 97.34, 77.41, 74.51, 73.61, 63.84 (d, *J* = 5.0 Hz), 44.48, 11.52 ppm.

^{31}P NMR (162 MHz, D_2O): δ = 2.79 (d, *J* = 10.0 Hz), 1.85, 1.18 (d, *J* = 9.7 Hz), 0.77 (d, *J* = 9.9 Hz), -0.20 (dt, *J* = 9.1, 9.1 Hz) ppm.

HRMS (ESI) for $C_{20}H_{33}NO_{26}P_6$ $[M-2H]^{2-}$: calculated 443.4785, found 443.4789.

2.14. Synthesis of 2-InsP$_1$ 12

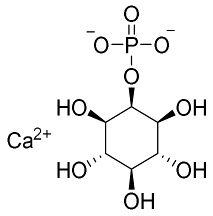

12

Chemical Formula: $C_6H_{11}CaO_9P$

Molecular Weight: 298.1968

$InsP_6$-dodecasodium salt (1.00 g, 901 µmol) was dissolved in NH_4OAc buffer (50 mM, pH = 6.3, 6.0 mL) Natuphos (300 µL, 10500 U/mL) was added and the solution was incubated at 37°C for 10 min. The reaction mixture was precipitated in EtOH (4 vol.), the precipitate was dissolved in water (20 mL) and precipitated by addition of $CaCl_2$ (640 mg, 5.77 mmol, 6.4 equiv.). The motherliquor was precipitated by addition of acetone (4 vol.) in two portions to obtain a white solid. The $CaCl_2$-precipitation of inorganic calcium phosphate and the following acetone precipitation of 2-InsP$_1$ was repeated six times, the resulting precipitate was dried under reduced pressure to obtain a white solid (231 mg, 775 µmol, 86% for the calcium salt).

^{1}H NMR (400 MHz, D_2O) δ = 4.56 (dt, *J* = 7.5, 2.5 Hz, 1H), 3.73 (t, *J* = 9.6 Hz, 2H), 3.54 (ddd, *J* = 9.9, 2.5, 1.2 Hz, 2H), 3.29 (t, *J* = 9.3 Hz, 1H) ppm.

^{13}C NMR (101 MHz, D_2O) δ = 76.13 (d, *J* = 5.6 Hz), 74.42, 72.88, 71.32, 71.29 ppm.

^{31}**P NMR** (162 MHz, D_2O) δ = 3.02 (d, *J* = 7.5 Hz) ppm.

HRMS (ESI) for $C_6H_{13}O_9P$ $[M-H]^-$: calculated 259.0224, found 259.0231.

2.15. Synthesis of 1-Fm-InsP$_1$ 13

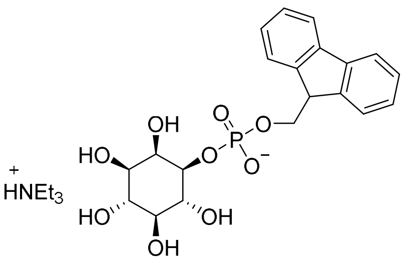

13

Chemical Formula: $C_{20}H_{22}O_9P^-$

Molecular Weight: 437.3613

1-Fm-InsP$_6$ **8** (50 mg, 52 µmol) was dissolved in NH_4OAc (50 mM in D_2O, pH = 6.3, 0.8 mL), Natuphos (0.2 mL, 10500 U/mL) was added and the reaction as incubated at 37°C for 2 days. The reaction mixture was precipitated in EtOH (30 mL), the precipitate was dissolved in water (1 mL), purified by MPLC on a C_{18}-Aq column using a Water:MeCN gradient (containing 10% triethylammonium acetate buffer and product containing fractions were lyophilised twice to obtain a white solid (3 mg). The motherliquor was concentrated *in vacuo*, the residue was dissolved in water (1 mL), purified by MPLC and product containing fractions were lyophilised twice to obtain a white solid. The material was used directly for subsequent basic deprotection.

^{1}**H NMR** (400 MHz, Deuterium Oxide): δ = 7.96 (dt, *J* = 8.0, 0.9 Hz, 2H), 7.84 – 7.76 (m, 2H), 7.55 (td, *J* = 7.5, 1.3 Hz, 2H), 7.51 – 7.44 (m, 2H), 4.50 – 4.42 (m, 1H), 4.41 – 4.31 (m, 2H), 3.91 (t, *J* = 2.8 Hz, 1H), 3.71 – 3.52 (m, 2H), 3.32 (dd, *J* = 10.0, 2.9 Hz, 1H), 3.23 (q, *J* = 7.3 Hz, 6H, $HNEt_3^+$), 3.18 (t, *J* = 9.2 Hz, 1H), 1.31 (t, *J* = 7.3 Hz, 9H, $HNEt_3^+$) ppm.

^{13}**C NMR** (101 MHz, Deuterium Oxide): δ = 144.23, 144.11, 141.03, 140.99, 128.02, 127.48, 125.32, 125.16, 120.16 (d, *J* = 2.5 Hz), 76.04 (d, *J* = 6.1 Hz), 73.83, 72.19, 71.29, 70.95, 70.62, 66.93 (d, *J* = 5.6 Hz), 46.69, 8.22 ppm.

^{31}**P{^{1}H} NMR** (162 MHz, Deuterium Oxide): δ = -0.34 ppm.

HRMS (ESI) for $C_{20}H_{23}O_9P$ $[M-H]^-$: calculated 437.1006, found 437.1008.

2.16. Synthesis of 1-$InsP_1$ 14

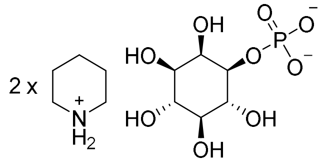

14
Chemical Formula: $C_6H_{11}O_9P^{2-}$
Molecular Weight: 258.1199

1-Fm-$InsP_1$ **13** (Starting from 1-Fm-$InsP_6$ **8** 50 mg, 52 µmol) was dissolved in water (5 mL), piperidine (0.5 mL) was added and the reaction mixture was incubated at RT for 30 min. The reaction mixture was precipitated in acetone (8 vol.), washed with acetone (10 mL) and the precipitate was discarded. The motherliquor was concentrated *in vacuo* to obtain a brown solid. (13 mg, 30 µmol, 58% starting from **158**; containing 2 equiv. piperidinium ions based on NMR.)

^{1}H NMR (400 MHz, D_2O): δ = 4.15 (t, J = 2.8 Hz, 1H), 3.83 (ddd, J = 9.8, 8.5, 2.8 Hz, 1H), 3.68 (t, J = 9.6 Hz, 1H), 3.57 (dd, J = 10.0, 9.1 Hz, 1H), 3.50 (dd, J = 10.0, 2.9 Hz, 1H), 3.26 (t, J = 9.2 Hz, 1H), 3.13 – 3.02 (m, 8H), 1.76 – 1.65 (m, 8H), 1.63 – 1.53 (m, 4H) ppm.

^{13}C NMR (101 MHz, D_2O): δ = 74.42, 74.31 (d, *J* = 5.1 Hz), 72.33, 72.25 (d, *J* = 3.8 Hz), 71.71 (d, *J* = 3.0 Hz), 70.85, 44.52, 22.21, 21.49 ppm.

^{31}P NMR (162 MHz, D_2O): δ = 3.96 (d, *J* = 8.4 Hz) ppm.

HRMS (ESI) for $C_6H_{13}O_9P$ $[M-H]^-$: calculated 259.0224, found 259.0230.

$[\alpha]^{20}_D$ = 3.1 (0.45, water, pH = 9).

2.17. Synthesis of 1,2-$InsP_2$ 15

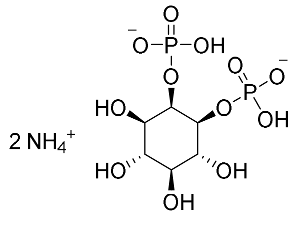

15
Chemical Formula: $C_6H_{12}O_{12}P_2^{2-}$
Molecular Weight: 338.0986

1-Fm-$InsP_6$ (15 mM, 0.66 µL) was digested by 6-Phytase from *Escherichia coli* (7500 U/mL, 1 U) in buffer (HEPES 50 mM, NaCl, 10 mM, glycerol 5%, DTT 2 mM, $MgCl_2$ 0.5 mM, pH = 4, total reaction volume 15 µL) at 28°C for 45 min and the solution was purified by MPLC on a C_{18}-Aq column using a Water:MeCN gradient (containing 10% triethylammonium acetate buffer and

product containing fractions were lyophilised twice to obtain a white solid (**HRMS** (CE-ESI-MS) for $C_{20}H_{23}O_{12}P_2$ $[M-H]^-$: calculated 517.0670, found 517.0671.).

The solid was dissolved in water (0.5 mL), piperidine (50 µL) was added and the resulting suspension was filtered after 30 min. The solution was lyophilized to obtain a brown solid. The crude was purified by SAX on a Q-sepharose column (*Capto Q-ImpRes, 1 mL*) on a NH_4HCO_3 gradient and product containing fractions were lyophilized to obtain a colorless solid (0.4 mg, quant.).

HRMS (CE-ESI-MS) for $C_6H_{14}O_{12}P_2$ $[M-H]^-$: calculated 338.9888, found 338.9892.

^{31}P NMR (162 MHz, Deuterium Oxide) δ = 4.85, 4.55 ppm.

2.18. Synthesis of Co(III) catalyst 19

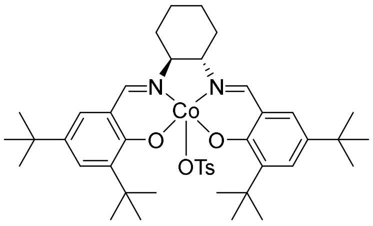

19

Chemical Formula: $C_{43}H_{59}CoN_2O_5S$

Molecular Weight: 774.9472

Based on literature[6]:

S,S-Co(II)-Salen (105 mg, 0.174 mmol, 1.0 equiv.) and *p*TsOH (36 mg, 0.189 mmol, 1.09 equiv.) were dissolved in CH_2Cl_2 (3 mL) and stirred with an open flask for 1.15 h. The reaction mixture was precipitated in ice cold pentane (24 mL), the motherliquor was concentrated *in vacuo*, the precipitates were combined, washed with pentante (25 mL) and dried under reduced pressure to obtain a green solid.

Analytical data is in accordance with literature[6]:

^{1}H NMR (300 MHz, DMSO-d_6): δ = 7.78 (s, 2H), 7.52 – 7.40 (m, 6H), 7.15 – 7.06 (m, 2H), 3.66 – 3.56 (m, 2H), 3.11 – 3.01 (m, 2H), 2.28 (s, 3H), 2.05 – 1.87 (m, 2H), 1.74 (s, 18H), 1.66 – 1.64 (m, 2H), 1.67 – 1.54 (m, 2H), 1.30 (s, 18H) ppm.

2.19. Synthesis of 2-glycerophosphoinositol 20

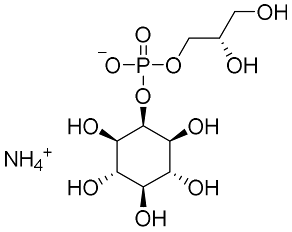

20

Chemical Formula: $C_9H_{18}O_{11}P^-$

Molecular Weight: 333.2063

A stocksolution of **19** (1 mg/mL, 99 µL, 767 µg, 1 µmol, 10 mol%) in MeCN was added to a solution of 2-InsP₁ × 2.0 TBA **12** (8.5 mg, 10 µmol, 1.0 equiv., 0.5 equiv. of TBA-OH were present as minor impurity) in MeCN (1.0 mL), DIPEA (2 µL; 1.48 mg, 12 µmol, 1.2 equiv.) and *S*-(-)-glycidol (2 µL, 2.23 mg, 30 µmol, 3.1 equiv.) was added and the reaction mixture was stirred with an open flask for 5 min, closed to the atmosphere with an septum and stirred overnight. The reaction mixture was diluted with water (8 mL) and purified by SAX on a Q-sepharose column (*Capto Q-ImpRes, 1 mL*) on a NH_4HCO_3 gradient. Fractions were analyzed by CE-qTOF-MS and product containing fractions were lyophilized thrice to obtain a white solid (1 mg, 3 µmol, 29% for the ammonium salt).

^{1}H NMR (400 MHz, Deuterium Oxide): δ = 4.61 (dt, *J* = 7.8, 2.4 Hz, 2H), 4.09 – 4.00 (m, 2H), 4.02 – 3.91 (m, 3H), 3.76 – 3.67 (m, 4H), 3.68 – 3.56 (m, 5H), 3.32 (t, *J* = 9.3 Hz, 2H) ppm.

^{31}P NMR (162 MHz, Deuterium Oxide): δ = 1.06 (ddd, *J* = 6.6 Hz) ppm.

HRMS (ESI) for $C_9H_{19}O_{11}P$ [M-H]$^-$: calculated 333.0592, found 333.0568.

2.20. Synthesis of 1-glycerophosphoinositol 21

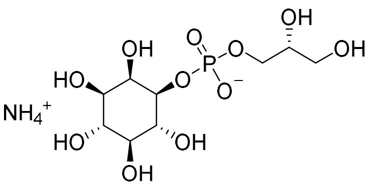

21

Chemical Formula: $C_9H_{18}O_{11}P^-$

Molecular Weight: 333.2063

A stocksolution of **19** in MeCN (10 mM, 123 µL, 10 mol%) was added to 1-InsP₁ × 2.0 TBA **14** (10.0 mg, 12 µmol, 1.0 equiv., 0.3 equiv. of TBA-OH were present as minor impurity) in MeCN (1.0 mL), DIPEA (2.5 µL, 14 µmol, 1.2 equiv.) and *S*-(-)-glycidol (2.5 µL, 28 µg, 3.1 equiv.) were added and the reaction mixture was stirred with an open flask for 5 min, closed to the atmosphere

with an septum and stirred overnight. The reaction mixture was diluted with water (8 mL) and purified by SAX on a Q-sepharose column (*Capto Q-ImpRes, 1 mL*) on a NH_4HCO_3 gradient. Fractions were analyzed by CE-qTOF-MS and product containing fractions were lyophilized thrice to obtain a white solid (1 mg, 3 µmol, 25% for the ammonium salt).

Analytical data is in accordance with literature[7]:

^{1}H{^{31}P}NMR (400 MHz, Deuterium Oxide): δ = 4.29 (dd, *J* = 2.8 Hz, 1H), 4.00 (dd, *J* = 9.9, 2.8 Hz, 1H), 4.04 – 3.90 (m, 3H), 3.78 (dd, *J* = 9.7 Hz, 1H), 3.74 – 3.61 (m, 3H), 3.58 (dd, *J* = 10.0, 2.8 Hz, 1H), 3.36 (dd, *J* = 9.4 Hz, 1H) ppm.

^{13}C NMR (101 MHz, Deuterium Oxide): δ = 76.29, 73.94, 72.22, 71.36 (d, *J* = 6.8 Hz), 71.18, 70.79, 70.74, 66.40 (d, *J* = 5.9 Hz), 62.07 ppm.

^{31}P{^{1}H} NMR (162 MHz, Deuterium Oxide): δ = -0.05 ppm.

HRMS (ESI) for $C_9H_{19}O_{11}P$ $[M-H]^-$: calculated 333.0592, found 333.0588.

3. Transient Phosphitylation monitored by ^{31}P-NMR

3.1. Transient Phosphitylation of ADP

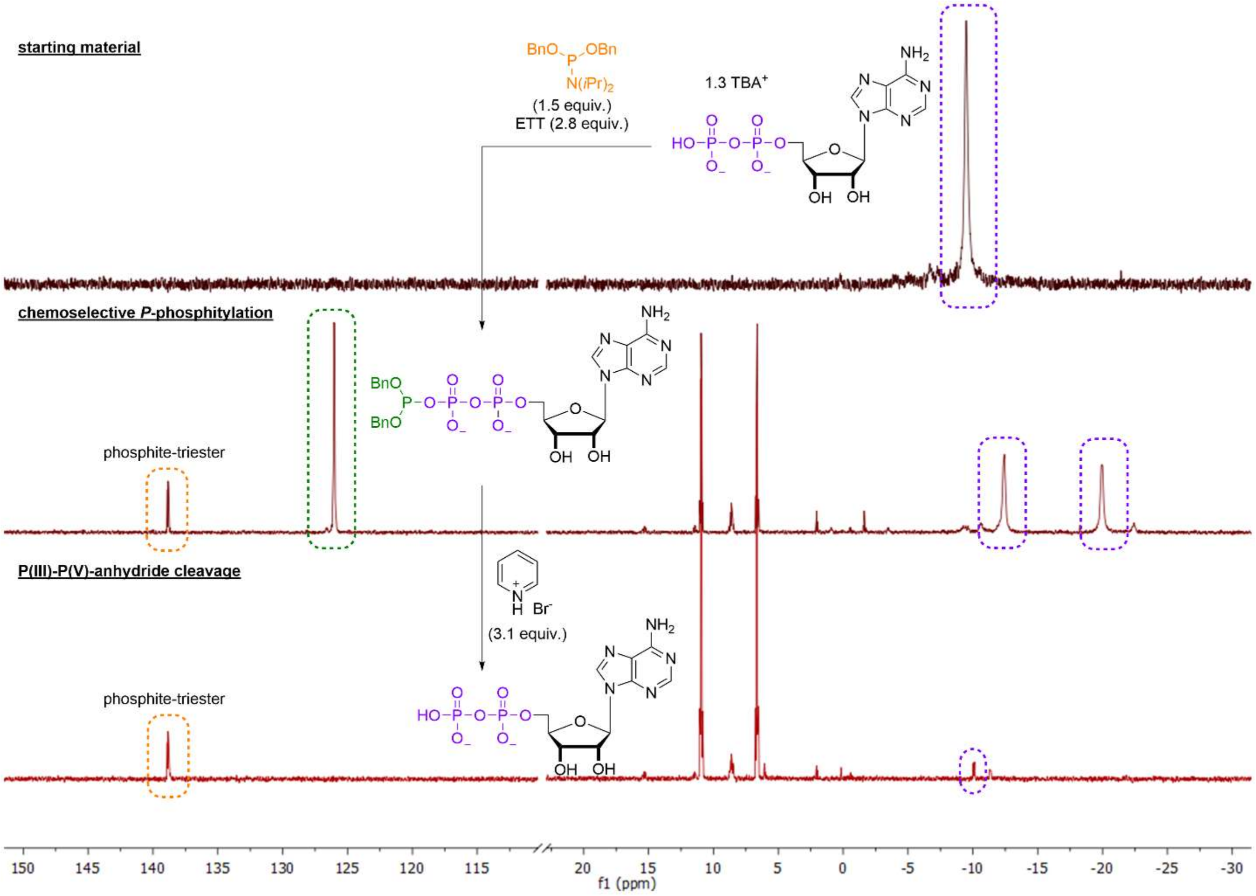

Supplementary Figure 1 Transient Phosphitylation of ADP monitored *via* ^{31}P-NMR. Coupling of ADP with commercially available dibenzyl-*N,N*-diisopropyl-P-amidite led to a P(III)-P(V) anhydride. Subsequent addition of pyridinium hydrobromide led to cleavage of the P(III)-P(V) anhydride. The chemical shifts of the observed intermediates are in accordance with literature.[8]

3.2. Transient Phosphitylation of 1-OH-InsP$_5$ with P-amidite 1

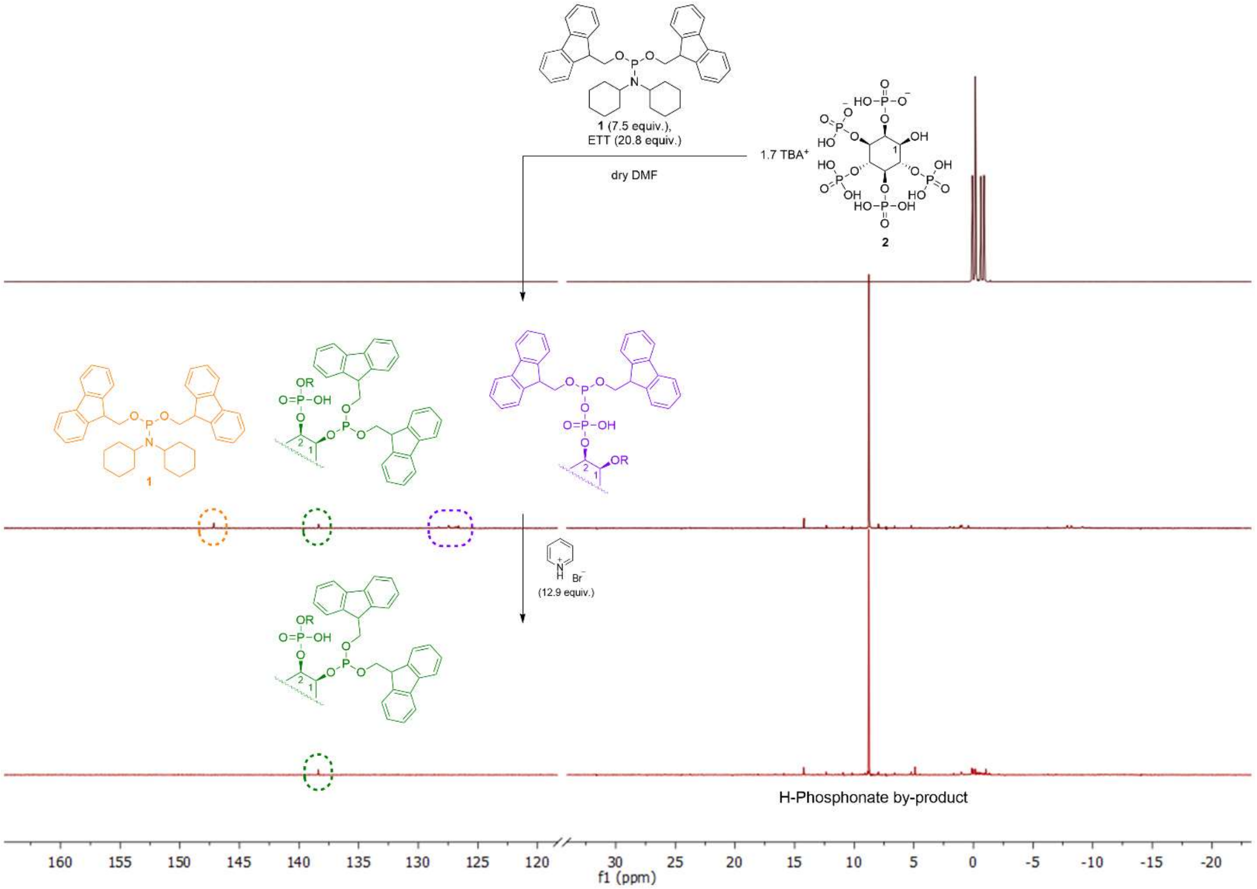

Supplementary Figure 2 Transient Phosphitylation of 1-OH-InsP$_5$ **2** monitored *via* $^{31}P\{^{1}H\}$-NMR. Coupling of **2** with P-amidite **1** led to a complex mixture of P(III)-P(V) anhydrides and to a P(OR)$_3$-species. Subsequent addition of pyridinium hydrobromide led to cleavage of the P(III)-P(V) anhydrides, while the P(OR)$_3$-species stayed intact. The chemical shifts of the observed intermediates are in accordance with literature.[8]

3.3. Transient Phosphitylation of 1-OH-InsP$_5$ with P-amidite 9

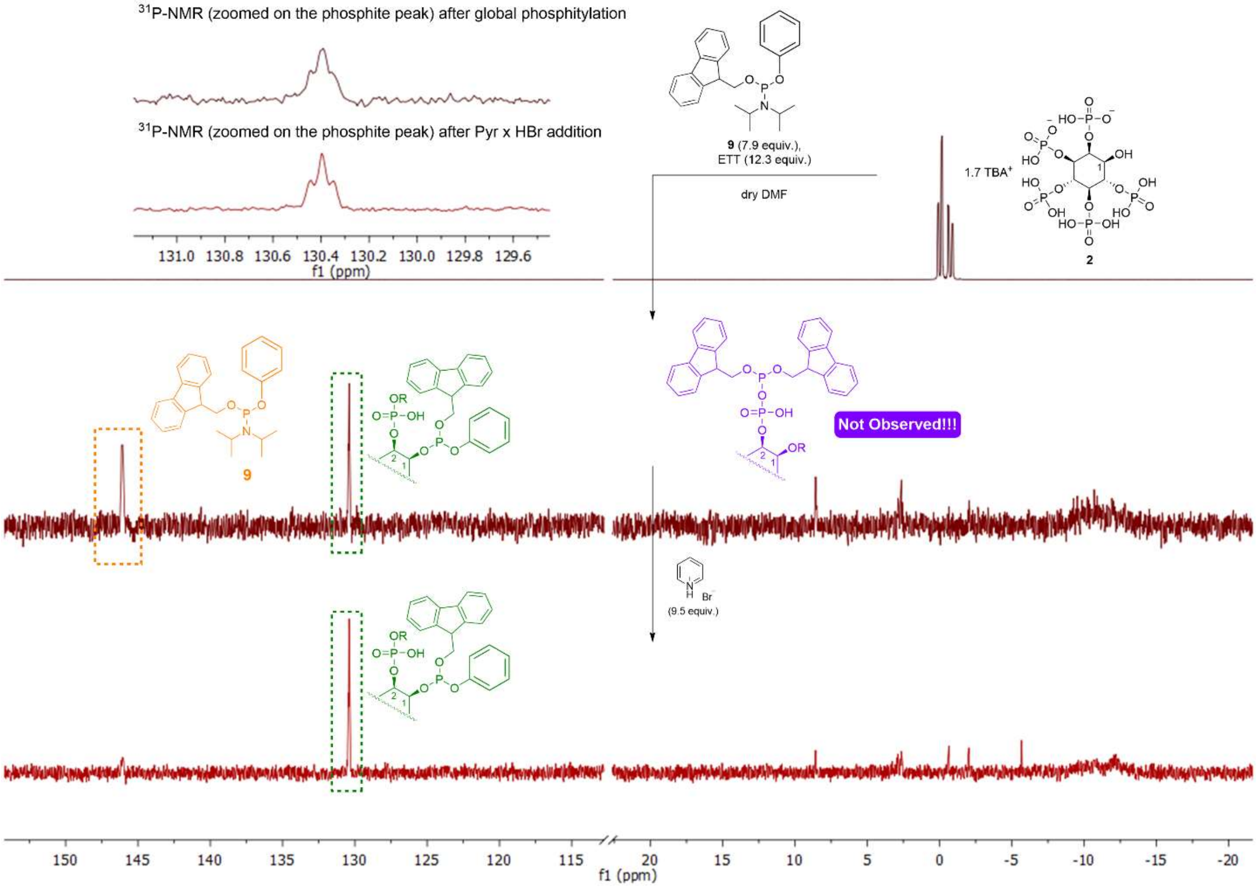

Supplementary Figure 3 Transient Phosphitylation of 1-OH-InsP$_5$ **2** monitored *via* ^{31}P-NMR. Coupling of **2** with P-amidite **9** led to a P(OR)$_3$-species, while no P(III)-P(V)-anhydrides were detectable. Subsequent addition of pyridinium hydrobromide led to complete consumption of unreacted P-amidite, P(OR)$_3$-species stayed intact. The chemical shifts of the observed intermediates are in accordance with literature.[8]

4. Towards the linearization of cyclic inositolpyrophosphates to $InsP_6$ derivatives

4.1. Screening for conditions to ring open the cyclic pyrophosphates of inositoltrispyrophosphate (ITPP)

4.1.1. Attempted nucleophilic ring-opening of ITPP with amines

Supplementary Table 4 Ring-opening screening of ITPP **25** with amine nucleophiles at RT. If not otherwise stated, 20 vol% amine were used. [1]Based on CE-qTOF-MS analysis (BGE: NH_4OAc 35 mM pH 9.7, CE voltage 30 kV, CE current: 23µA, injection 100 mbar, 15 s (30 nL)) and ^{31}P-NMR. [2]Based on CE-qTOF-MS analysis (BGE: NH_4OAc 35 mM pH = 9.7, CE voltage 30 kV, CE current: 23µA, injection 100 mbar, 15 s (30 nL)) ring opening occurred to some degree, but was not quantifiable by NMR.

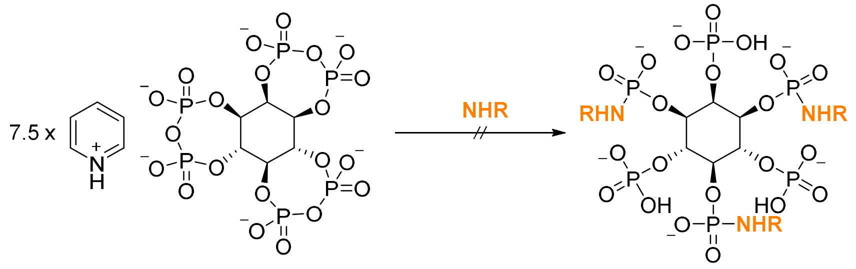

25

Entry	Amine	Reaction Time	Solvent	Outcome[1]
1	Dimethyl amine (40 vol%)	Overnight	Water	/*
2	*o*-Hydroxylaniline (128 equiv.)	Overnight	Water:DMSO (5:2)	/
3	Hydroxylamine Hydrochloride (103 equiv.)	Overnight	Water	/
4	Morpholine	Overnight	Water	/[2]
5	Morpholine	Overnight	MeCN	/
6	Hydrazine	3 days	Water	One opened pyrophosphate
7	Hydrazine	Overnight	MeCN	/
8	Propargyl Amine	3 days	Water	/[2]
9	Ammonia	3 days	Water	/[2]
10	Piperidine	Overnight	MeCN	/
11	Piperidine	Overnight	DMF	/

4.1.2. Towards hydrolysis of ITPP to $InsP_6$

Supplementary Table 5 Ring-opening screening of ITPP **25**. The reaction outcome was analyzed by CE-qTOF-MS (BGE: NH_4OAc 35 mM pH = 9.7, CE voltage 30 kV, CE current: 23µA, injection 100 mbar, 15 s (30 nL)) and $^{31}P\{^1H\}$-NMR. [1]No complete conversion was observed. [2]Dephosphorylation was observed by ^{31}P-NMR. [3]Based on $^{31}P\{^1H\}$-NMR the 1/3'-2' pyrophosphate was hydrolysed.[4]

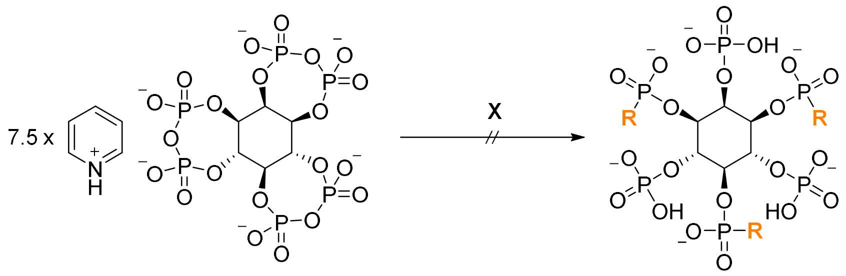

25 InsP6

Entry	X	T	Reaction Time	Solvent	Outcome
1	DBU	65°C	60 min	DMSO:water (6:1)	/
2	DABCO	65°C	60 min	DMSO:water (6:1)	/
3	Morpholine	65°C	60 min	DMSO:water (6:1)	/
4	Hydrazine	65°C	60 min	DMSO:water (6:1)	/
5	NaI	65°C	60 min	DMSO:water (6:1)	/
6	TMS-Cl	65°C	60 min	DMSO:water (6:1)	Complete ring-opening[1]
7	AcCl	0°C to RT	overnight	MeOH	Complete ring-opening
8	AcCl	0°C to RT	overnight	BnOH:DMSO:water (5:2.5:1)	/
9	AcCl	65°C	60 min	DMSO:water (6:1)	Complete ring-opening[1]
10	TfOH	65°C	60 min	DMSO:water (6:1)	Complete ring-opening[2]
11	*p*TSA × H_2O	65°C	60 min	DMSO:water (6:1)	Partial ring-opening[3]
12	CSA	65°C	60 min	DMSO:water (6:1)	Partial ring-opening[3]
13	Dichloroacetic acid	65°C	60 min	DMSO:water (6:1)	Partial ring-opening[3]
14	TFA	65°C	60 min	DMSO:water (6:1)	Partial ring-opening[3]
15	AgOTf	65°C	60 min	DMSO:water (6:1)	/
16	*Amberlite* 15 H^+-Form	65°C	60 min	DMSO:water (6:1)	/
17	Conc. HCl (40vol%)	65°C	2 days	water	/
18	HBr (30% in acetic acid, 30vol%)	80°C	2 h	water	Complete ring-opening
19	Phenyl Phosphate (6 equiv.)	RT	overnight	water	/
20	Benzyl Phosphate (6 equiv.)	RT	overnight	water	/

4.2. NMR monitoring of the hydrolysis of ITPP

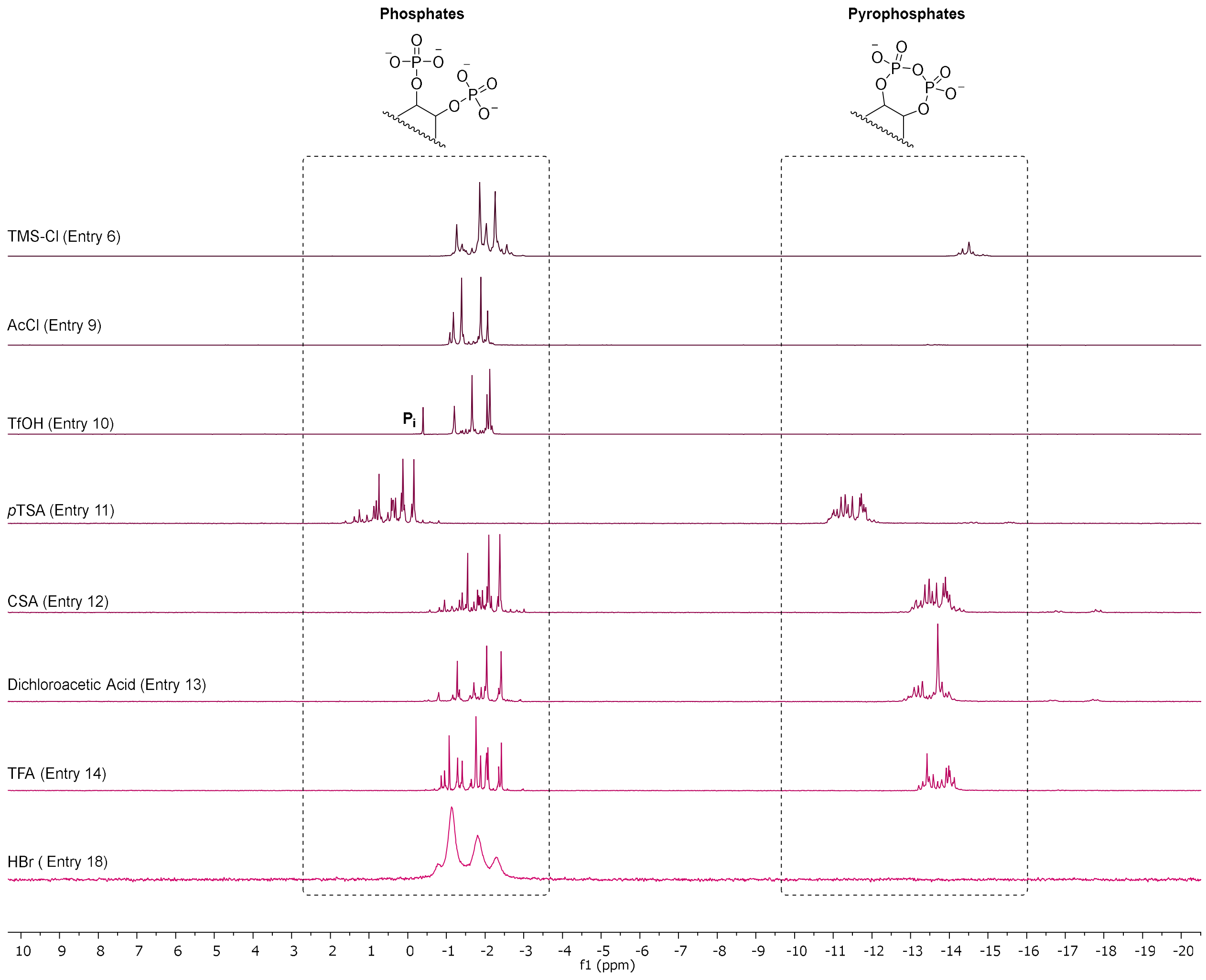

Supplementary Figure 4 ^{31}P-NMR comparison of (partial)-ring openings of ITPP **25** with organic acids in DMSO:water (see Supplementary Figure 5 Entry 6 and 9 – 14).

4.3. ^{31}P-NMR monitoring of the hydrolysis of 1-Fm-InsP$_6$ 8

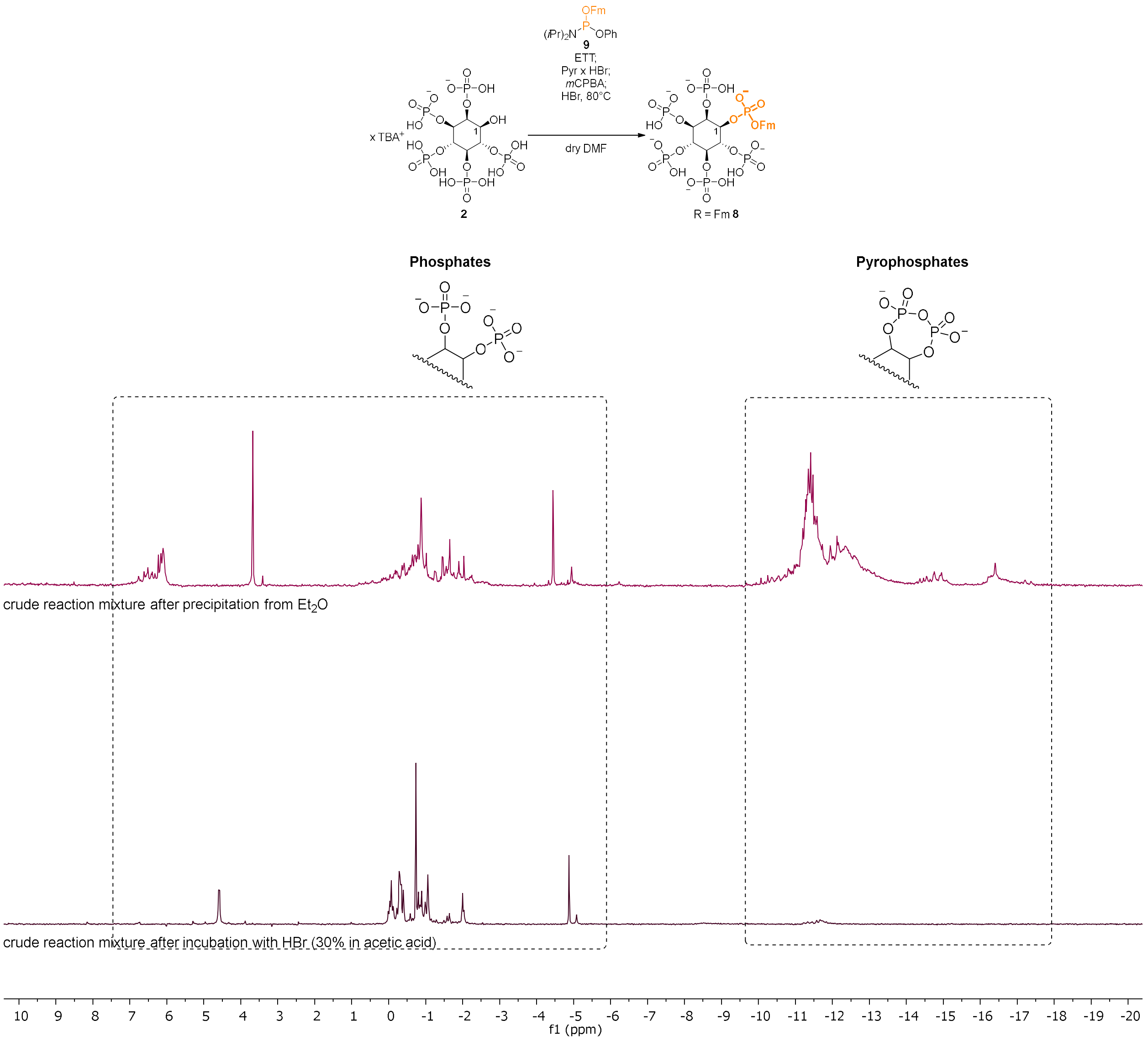

Supplementary Figure 5 The ring opening of cyclic 1-Fm-InsP$_6$ derivatives by HBr treatment was monitored by ^{31}P-NMR.

4.4. ^{31}P-NMR monitoring of the hydrolysis of 1-DEACM-InsP6 11

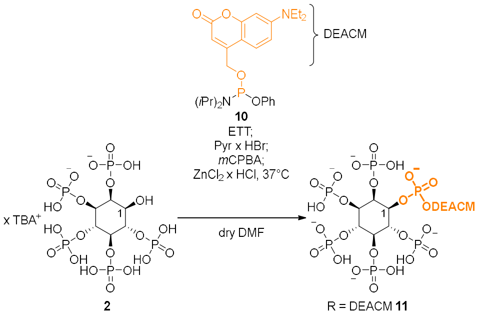

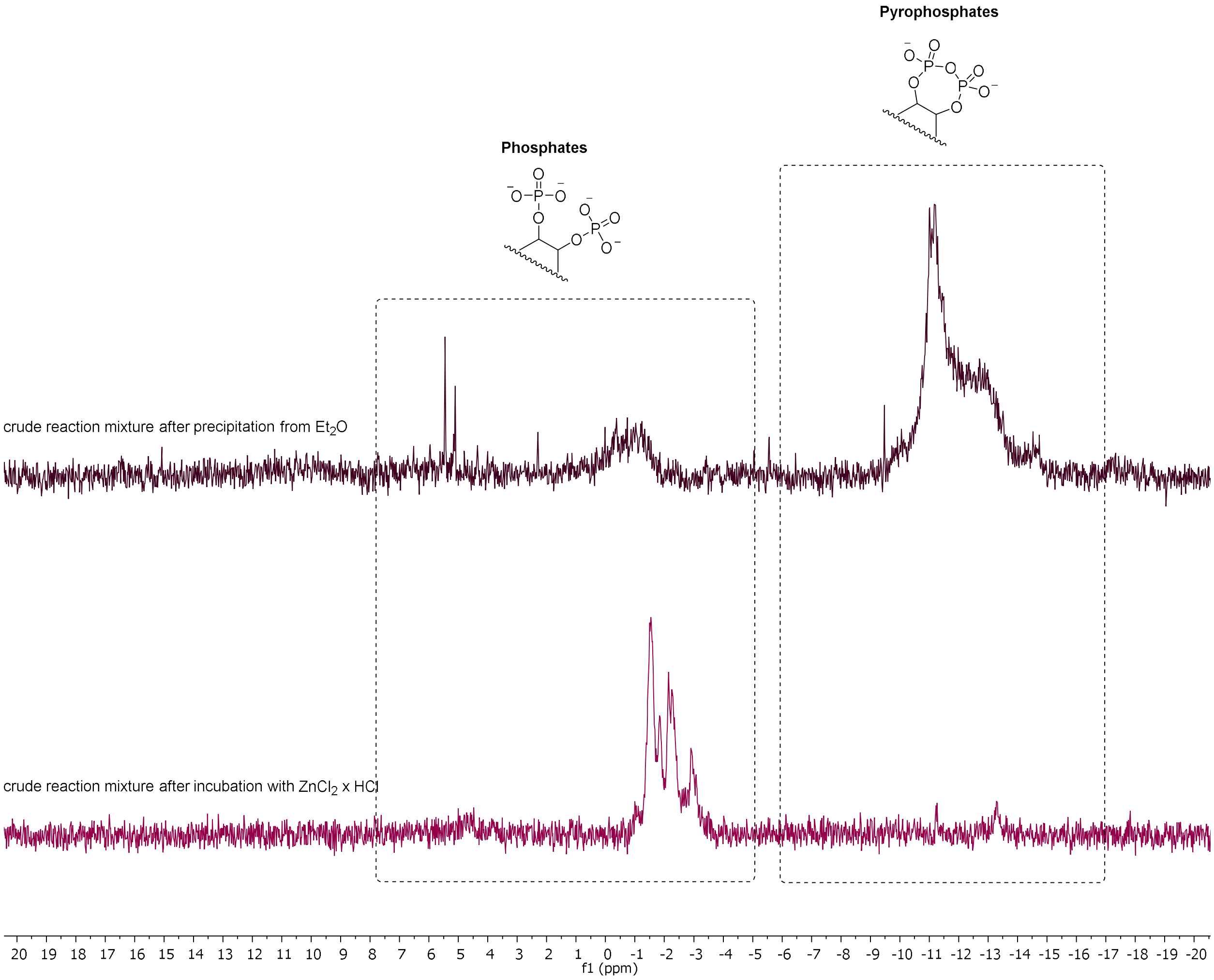

Supplementary Figure 6 The ring opening of cyclic 1-DEACM-InsP$_6$ derivatives by $ZnCl_2 \times HCl$ treatment was monitored by ^{31}P-NMR.

4.5. 2D-NMR of 1-Fm-InsP$_6$ 8

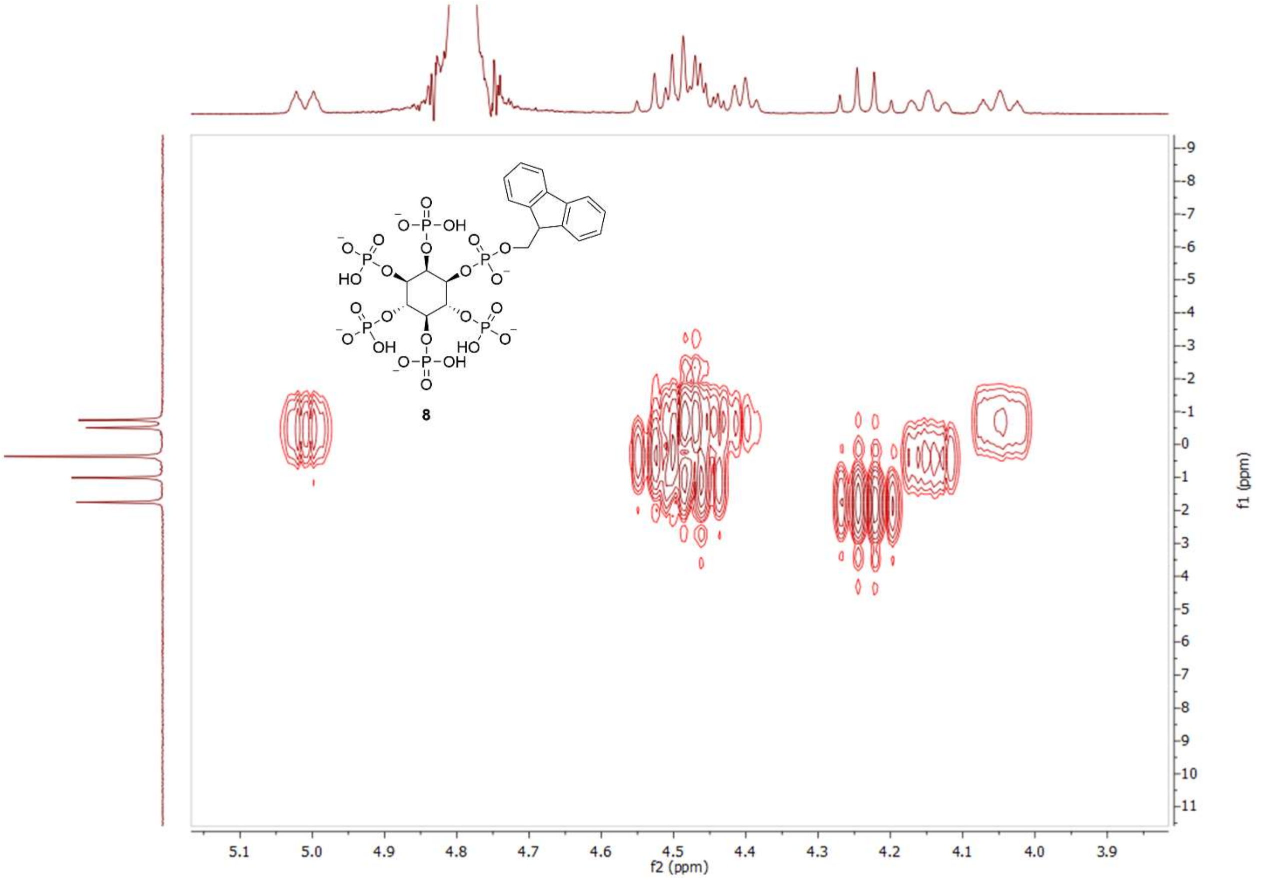

Supplementary Figure 7 ^{31}P-^{1}H-HMBC-NMR of 1-Fm-InsP$_6$ **8**.

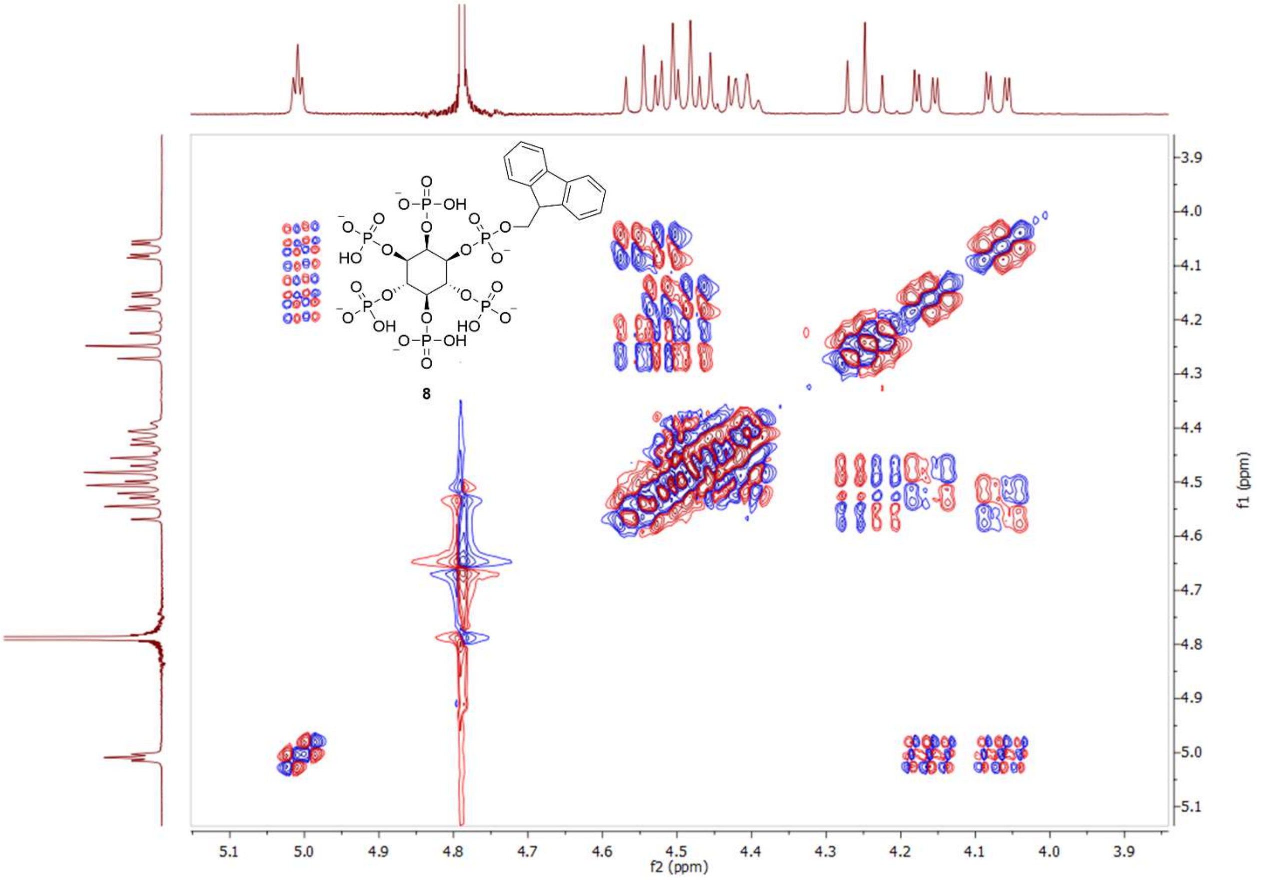

Supplementary Figure 8 {^{31}P}-^{1}H-^{1}H-DQF-COSY-NMR of 1-Fm-InsP$_6$ **8**.

5. Natuphos digest of $InsP_6$

Supplementary Table 6 Screening for optimal conditions for the Natuphos (10500 U/mL) digest of $InsP_6$. 5vol% of Natuphos (10500 U/mL) solution (270 mg/mL) was used. [1]Conditions used in literature.[9]

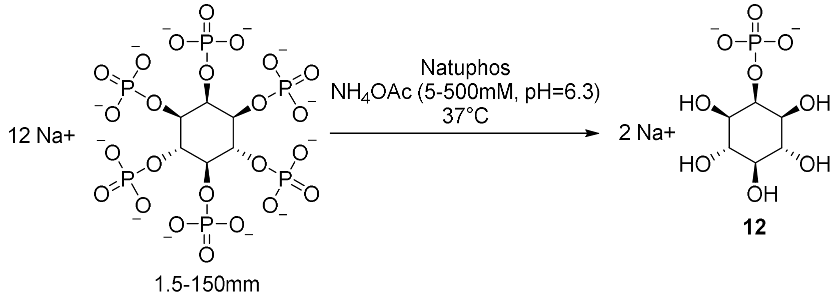

Entry	$[InsP_6]$	$[NH_4OAc]$	Main Product after 10 min
1	150 mM	500 mM	2-$InsP_1$
2	150 mM	50 mM	2-$InsP_1$
3	150 mM	5 mM	2-$InsP_1$
4	150 mM	50 mM	2-$InsP_1$
5[1]	15 mM	50 mM	2-$InsP_1$
6	1.5 mM	50 mM	2-$InsP_1$

5.1. Purification of 2-InsP$_1$ *via* precipitation monitored *via* ^{31}P-NMR monitoring

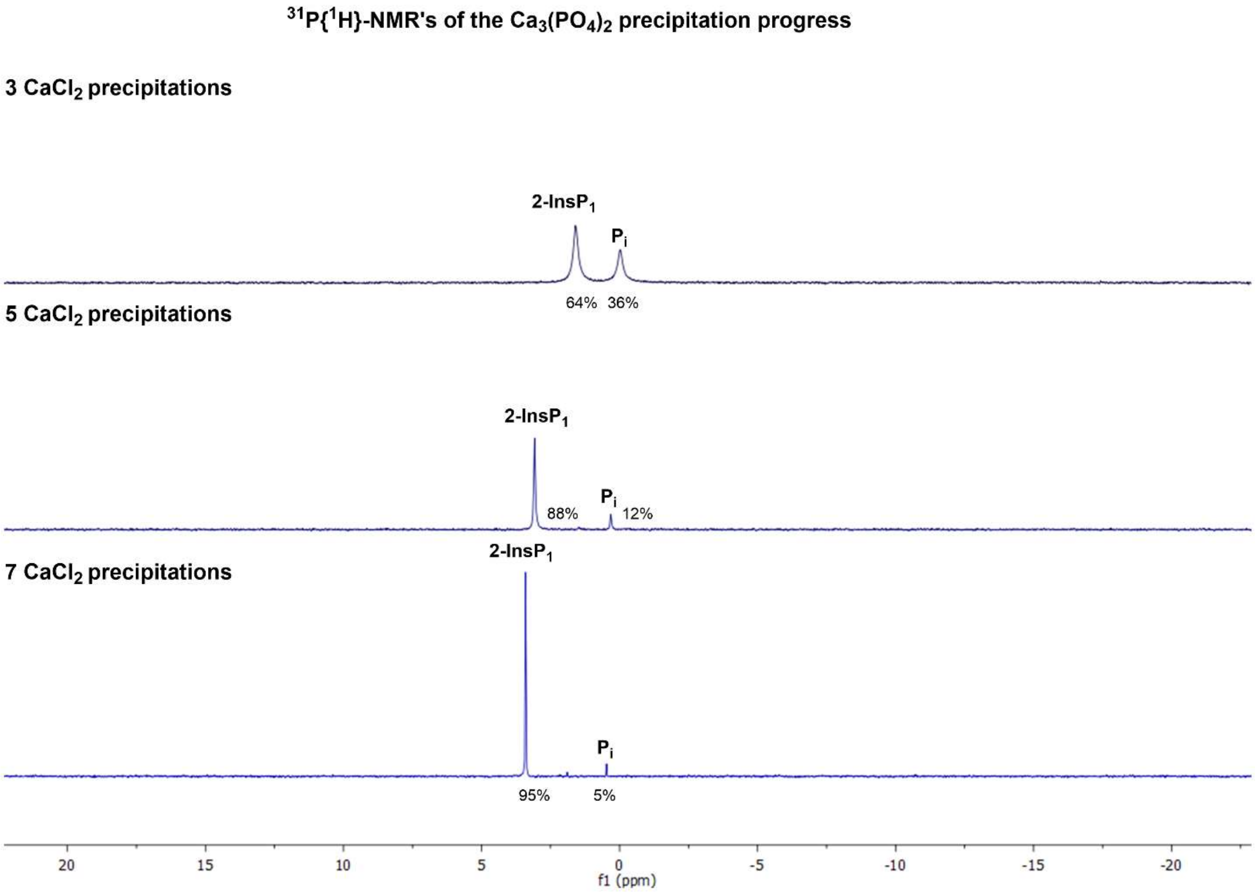

Supplementary Figure 9 The $Ca_3(PO_4)_2$ precipitation progress during 2-InsP$_1$ purification was monitored by $^{31}P\{^1H\}$-NMR.

5.2. Natuphos digest of 1-Fm-InsP$_6$ 8 monitored *via* ^{31}P-NMR

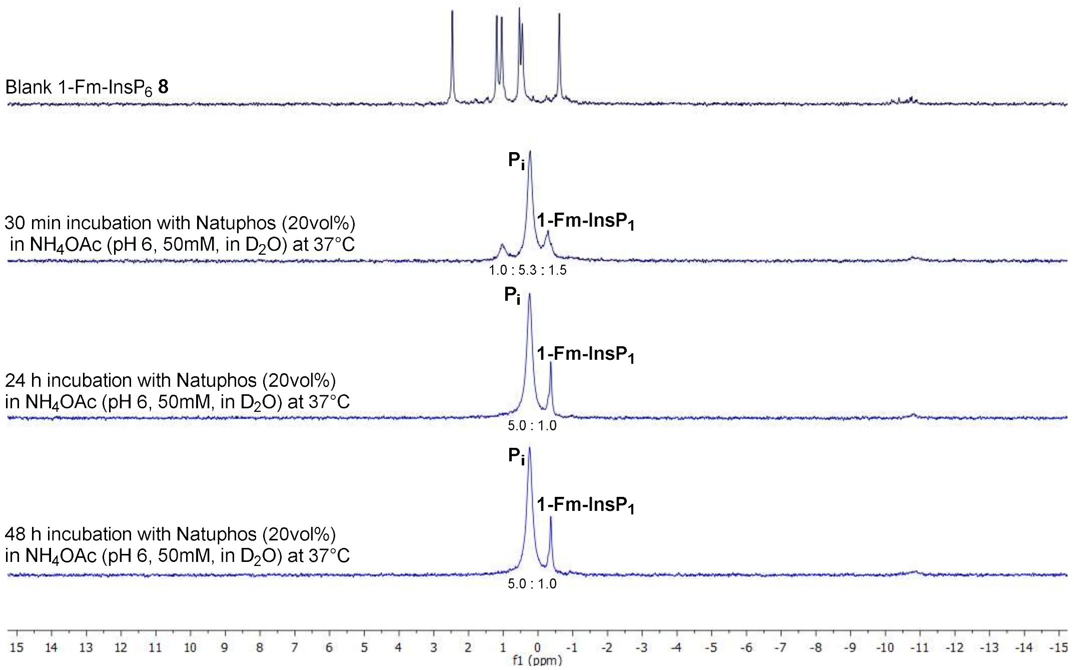

Supplementary Figure 10 The Natuphos (10500 U/mL) digest of 1-Fm-InsP$_6$ **8** to 1-Fm-InsP$_1$ **13** and inorganic phosphate was monitored by ^{31}P{^{1}H}-NMR

6. Identification of the formed $InsP_2$ isomers obtained by dephosphorylation of 1-Fm-$InsP_6$ 8

6.1. by Natuphos – spiking experiments with $InsP_2$ standards

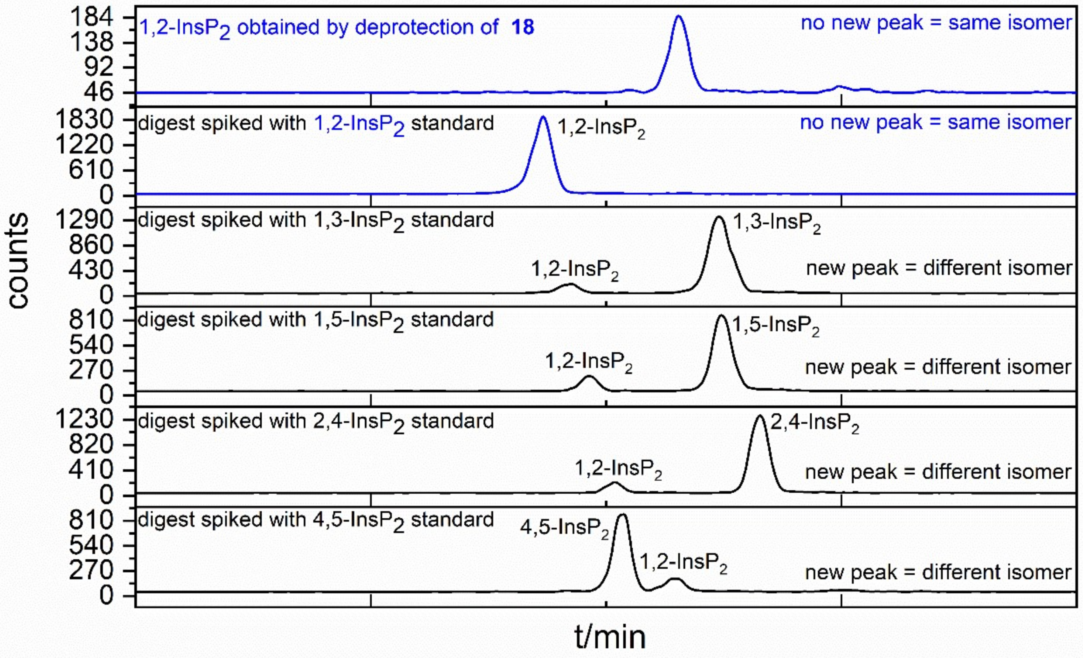

Supplementary Figure 11 CE-QQQ-MS (BGE: NH_4OAc 35 mM pH = 9.7, CE voltage: 30 kV, CE current: 23 µA, injection: 100 mbar, 10 s (20 nL)) analysis of the digest of 1-Fm-$InsP_6$ **8** by Natuphos (10500 U/mL) after subsequent basic deprotection. Spiking experiments with defined isomers allowed to identification of 1,2-$InsP_2$.[10]

6.2. by 6-Phytase from *Escherichia coli (7500 U/mL)* – spiking experiments with $InsP_2$ standards

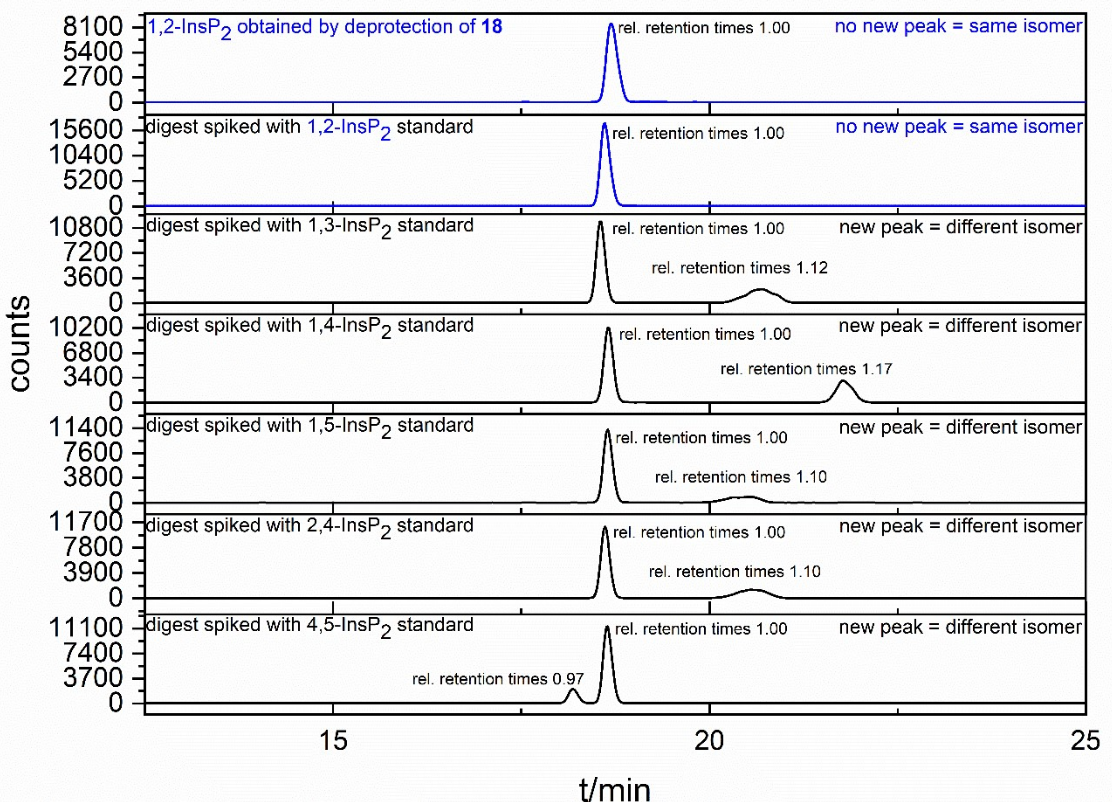

Supplementary Figure 12 CE-QQQ-MS (BGE: NH_4OAc 35 mM pH = 9.7, CE voltage: 30 kV, CE current: 23 µA, injection: 100 mbar, 10 s (20 nL)) analysis of the digest of 1-Fm-$InsP_6$ **8** with 6-Phytase from *Escherichia coli (7500 U/mL)* after subsequent basic deprotection. Spiking experiments with defined isomers allowed to identification of 1,2-$InsP_2$.[10]

7. Phytase Screen

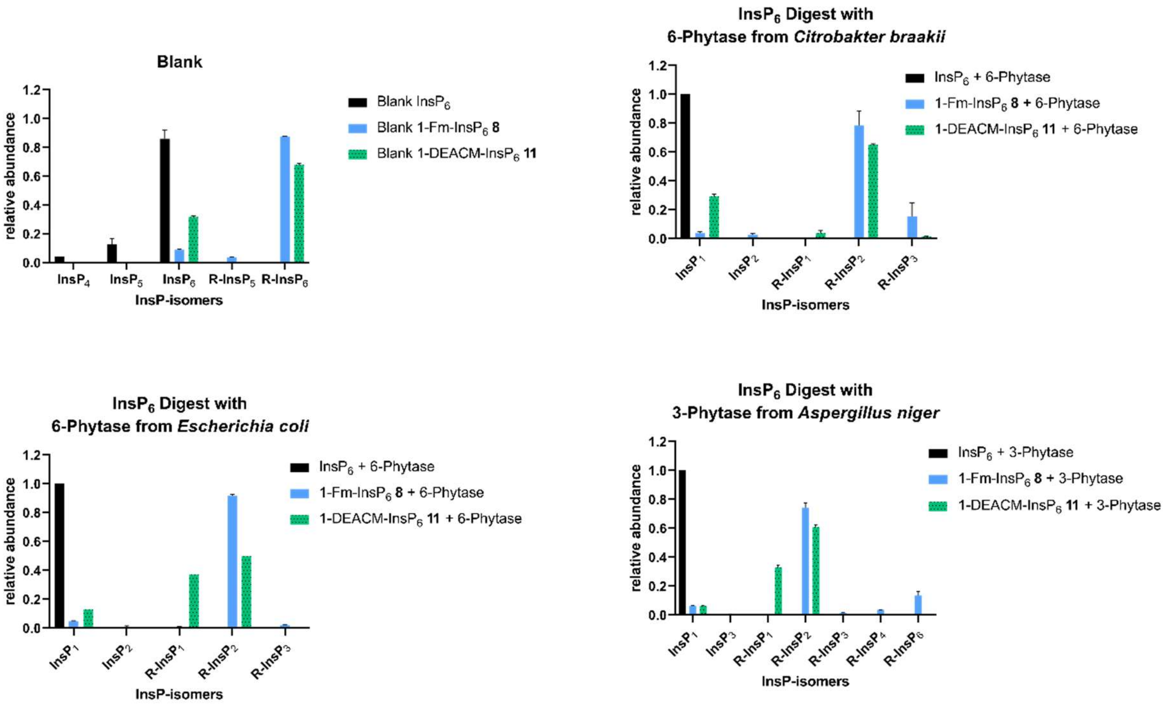

Supplementary Figure 13 CE-qTOF-MS (BGE: NH_4OAc 35 mM pH = 9.7, CE voltage: 30 kV, CE current: 23µA, injection: 100 mbar, 15 s (30 nL)) analysis of the digest of modified $InsP_6$ derivatives with different phytases (6-phytase from *Citrobakter braaki,* 30000 U/mL; 6-Phytase from *Escherichia coli,* 5000 U/mL; 3-phytase from *Asperigillus niger*, 10000 U/mL).

7.1. Identification of the formed $InsP_1$ *via* spiking experiments

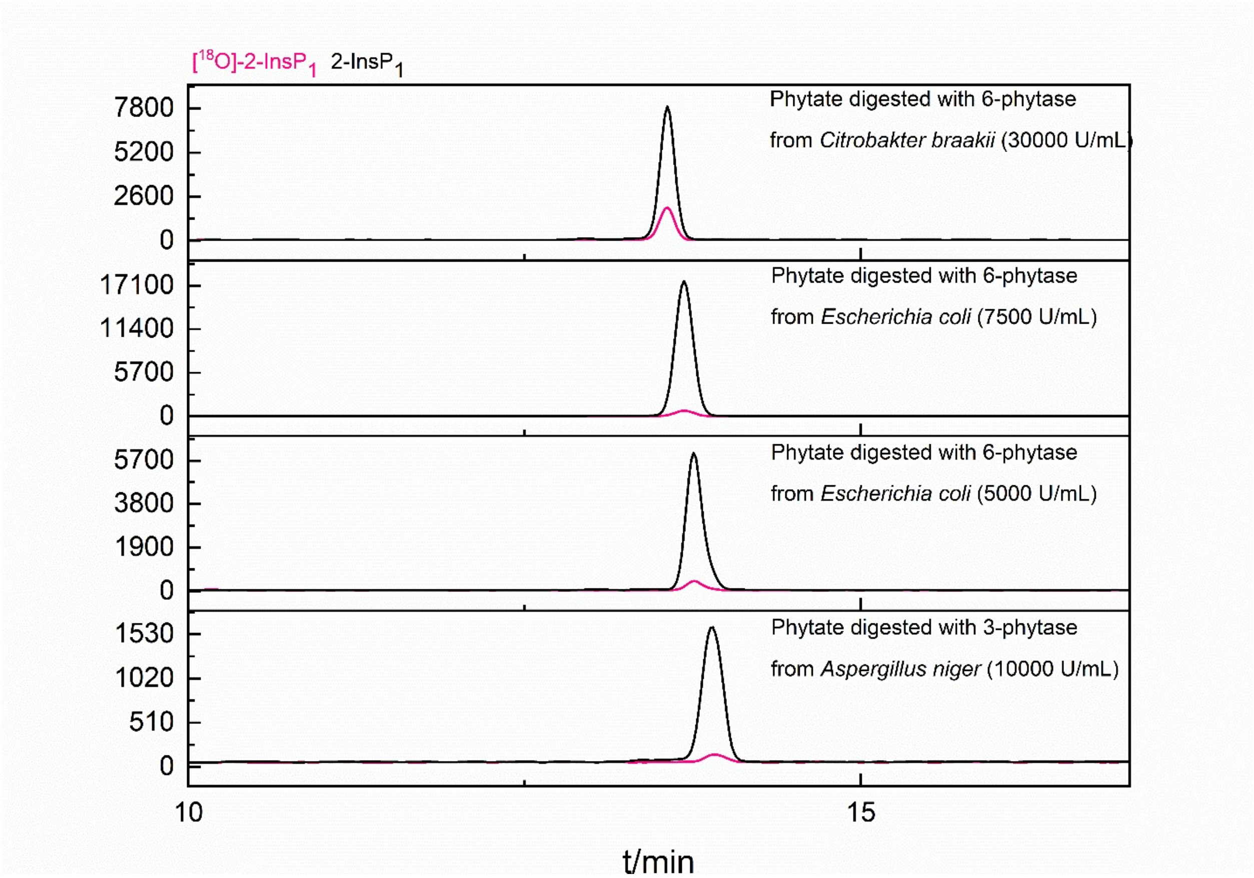

Supplementary Figure 14 CE-QQQ-MS (BGE: NH_4OAc 35 mM pH = 9.7, CE voltage: 30 kV, CE current: 23 µA, injection: 100 mbar, 10 s (20 nL)) spiking experiments with [^{18}O]-2-$InsP_1$ allowed identification of 2-$InsP_1$ as product of the $InsP_6$ digests of the tested phytases.

8. Co(III) catalysis screening

Supplementary Table 7 Reaction screening of the ring opening of *S*-(-)-glycidol with 2-InsP$_1$ **12** under Co(III) catalysis using JACOBSENS catalyst **19**.[1]Based on $^{31}P\{^1H\}$-NMR integration. [2]2-InsP$_1$ × 1.3 TBA was poorly soluble in MeCN. [3]Based on ^1H-NMR 0.6 equiv. TBA-OH were present.

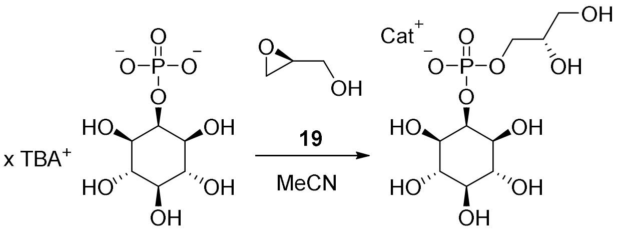

12 **20**

Entry	TBA$^+$	*S*-(-)-glycidol	19	t	12:20[1]
1[2]	1.3 equiv.	1.0 equiv.	10mol%	Overnight	/
2[2]	1.3 equiv.	1.5 equiv.	10mol%	Overnight	/
3[2]	1.3 equiv.	2.0 equiv.	10mol%	Overnight	/
4[2]	1.3 equiv.	1.0 equiv.	5mol%	Overnight	/
5[2]	1.3 equiv.	1.5 equiv.	5mol%	Overnight	/
6[2]	1.3 equiv.	2.0 equiv.	5mol%	Overnight	1.0:0.16
7	1.5 equiv.	0.7 equiv.	35mol%	8 days	1.0:0.3
8	1.5 equiv.	1.0 equiv.	35mol%	2 days	1.0:0.3
9	1.5 equiv.	1.4 equiv.	35mol%	8 days	1.0:0.3
10	1.5 equiv.	0.7 equiv.	21mol%	8 days	1.0:0.3
11	1.5 equiv.	0.7 equiv.	7mol%	8 days	1.0:0.4
12[3]	2.6 equiv.	1.0 equiv.	10mol%	Overnight	1.0:0.1
13[3]	2.6 equiv.	1.5 equiv.	10mol%	Overnight	1.0:0.1
14[3]	2.6 equiv.	2.0 equiv.	10mol%	overnight	1.0:0.1

8.1. NMR monitoring for extend reaction times

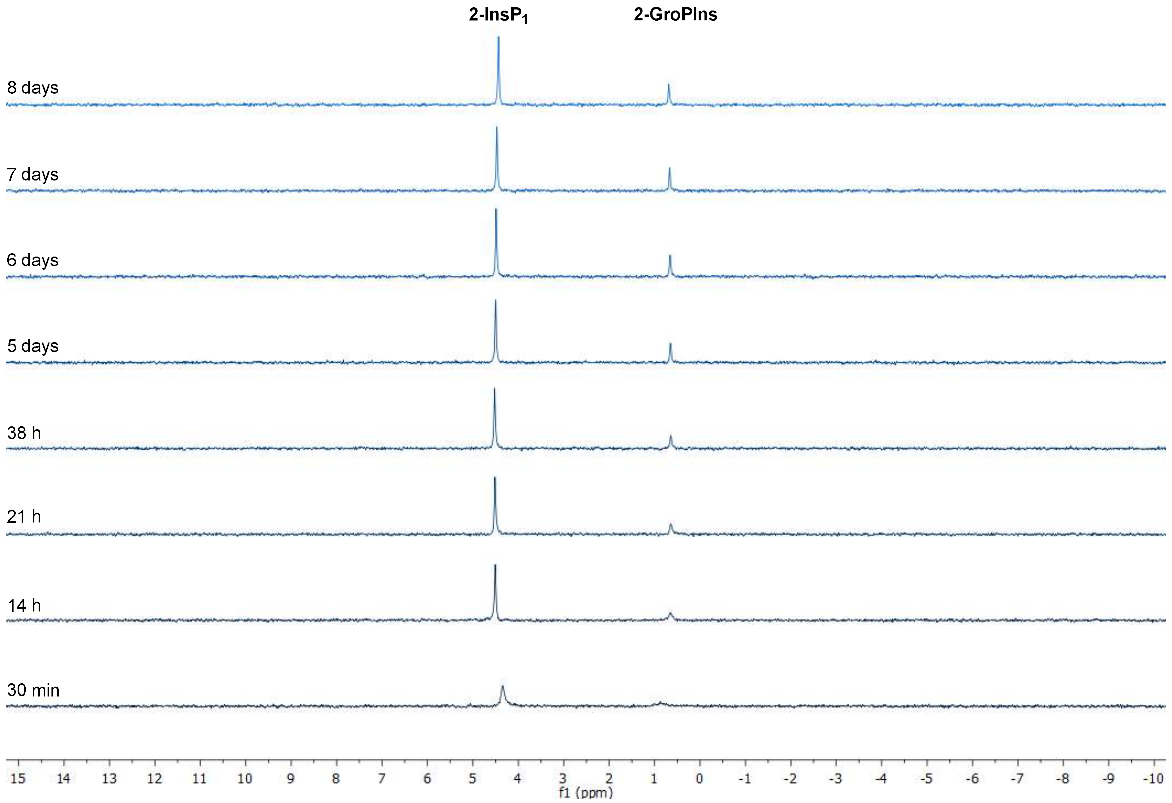

Supplementary Figure 15 $^{31}P\{^{1}H\}$-NMR of the ring opening of *S*-(-)-glycidol with 2-InsP$_1$ **12** catalysed by **19** (Supplementary Table 7, Entry 11).

9. Literature

1 G. R. Fulmer, A. J. M. Miller, N. H. Sherden, H. E. Gottlieb, A. Nudelman, B. M. Stoltz, J. E. Bercaw and K. I. Goldberg, *Organometallics*, 2010, **29**, 2176–2179.

2 D. Blüher, D. Laha, S. Thieme, A. Hofer, L. Eschen-Lippold, A. Masch, G. Balcke, I. Pavlovic, O. Nagel, A. Schonsky, R. Hinkelmann, J. Wörner, N. Parvin, R. Greiner, S. Weber, A. Tissier, M. Schutkowski, J. Lee, H. Jessen, G. Schaaf and U. Bonas, *Nat. Commun.*, 2017, **8**, 2159.

3 T. Weinrich, M. Gränz, C. Grünewald, T. F. Prisner and M. W. Göbel, *European J. Org. Chem.*, 2017, **2017**, 491–496.

4 L. F. Johnson and M. E. Tate, *Can. J. Chem.*, 1969, **47**, 63–73.

5 K. C. Fylaktakidou, J.-M. Lehn, R. Greferath and C. Nicolau, *Bioorg. Med. Chem. Lett.*, 2005, **15**, 1605–1608.

6 L. P. C. Nielsen, C. P. Stevenson, D. G. Blackmond and E. N. Jacobsen, *J. Am. Chem. Soc.*, 2004, **126**, 1360–1362.

7 K. S. Bruzik, Z. Guan, S. Riddle and M.-D. Tsai, *J. Am. Chem. Soc.*, 1996, **118**, 7679–7688.

8 H. J. Jessen, T. Dürr-Mayer, T. M. Haas, A. Ripp and C. C. Cummins, *Acc. Chem. Res.*, 2021, **54**, 4036–4050.

9 R. Greiner, *Prep. Biochem. Biotechnol.*, 2021, **51**, 985–989.

10 G. Liu, E. Riemer, R. Schneider, D. Cabuzu, O. Bonny, C. A. Wagner, D. Qiu, A. Saiardi, A. Strauss, T. Lahaye, G. Schaaf, T. Knoll, J. P. Jessen and H. J. Jessen, *RSC Chem. Biol.*, 2023, **4**, 300–309.

10. Capillary Electrophoresis Integration

10.1. Blank

Supplementary Table 8 CE-qTOF-MS (CE-qTOF-MS (BGE: NH_4OAc 35 mM pH = 9.7, CE voltage: 30 kV, CE current: 23µA, injection: 100 mbar, 15 s (30 nL)) analysis of the blank of $InsP_6$ derivatives incubated under dephosphorylation conditions in the absence of phytase.

Blank

	$InsP_6$			1-Fm-$InsP_6$			1-DEACM-$InsP_6$		
$InsP_1$									
$InsP_2$									
$InsP_3$									
$InsP_4$		0.04249							
$InsP_5$	0.10514	0.1711	0.10348						
$InsP_6$	0.89486	0.78641	0.89652	0.08378	0.09227	0.08797	0.31129	0.32104	0.32252
R-$InsP_1$									
R-$InsP_2$									
R-$InsP_3$									
R-$InsP_4$									
R-$InsP_5$				0.03735	0.03946	0.03851			
R-$InsP_6$				0.87887	0.86827	0.87351	0.68871	0.67896	0.67748

10.2. Dephosphorylation of phytate with 6-phytase from *Citrobacter braakii* (30000 U/mL)

Supplementary Table 9 CE-qTOF-MS (CE-qTOF-MS (BGE: NH_4OAc 35 mM pH = 9.7, CE voltage: 30 kV, CE current: 23µA, injection: 100 mbar, 15 s (30 nL)) analysis digest of $InsP_6$ derivatives with 6-phytase from *Citrobacter braakii* (30000 U/mL).

Phytase 6

	$InsP_6$			1-Fm-$InsP_6$			1-DEACM-$InsP_6$		
$InsP_1$	1.000	1.000	1.000	0.03347	0.04877	0.03255	0.29791	0.27684	0.30418
$InsP_2$				0.03168	0.01397	0.03319			
$InsP_3$									
$InsP_4$									
$InsP_5$									
$InsP_6$									
R-$InsP_1$							0.04121	0.05571	0.02471
R-$InsP_2$				0.72665	0.89827	0.72564	0.6484	0.65481	0.65543
R-$InsP_3$				0.20821	0.039	0.20861	0.01248	0.01265	0.01568
R-$InsP_4$									
R-$InsP_5$									
R-$InsP_6$									

10.3. Dephosphorylation of phytate with 6-phytase from *Escherichia coli* (7500 U/mL)

Supplementary Table 10 CE-qTOF-MS (CE-qTOF-MS (BGE: NH_4OAc 35 mM pH = 9.7, CE voltage: 30 kV, CE current: 23µA, injection: 100 mbar, 15 s (30 nL)) analysis digest of $InsP_6$ derivatives with 6-phytase from *Escherichia coli* (7500 U/mL).

Phytase 9

	$InsP_6$			1-Fm-$InsP_6$			1-DEACM-$InsP_6$		
$InsP_1$	1.000	1.000	1.000	0.06348	0.06870	0.05893	0.11411	0.04383	0.04181
$InsP_2$									
$InsP_3$									
$InsP_4$									
$InsP_5$									
$InsP_6$									
R-$InsP_1$				0.01495	0.01316	0.01642	0.33670	0.63468	0.64847
R-$InsP_2$				0.83251	0.81683	0.88445	0.54919	0.32149	0.30972
R-$InsP_3$				0.03087	0.03256	0.21370			
R-$InsP_4$				0.05819	0.06876	0.01884			
R-$InsP_5$									
R-$InsP_6$									

10.4. Dephosphorylation of phytate with 6-phytase from *Escherichia coli* (5000 U/mL)

Supplementary Table 11 CE-qTOF-MS (CE-qTOF-MS (BGE: NH_4OAc 35 mM pH = 9.7, CE voltage: 30 kV, CE current: 23µA, injection: 100 mbar, 15 s (30 nL)) analysis digest of $InsP_6$ derivatives with 6-phytase from *Escherichia coli* (5000 U/mL).

Phytase 20

	$InsP_6$			1-Fm-$InsP_6$			1-DEACM-$InsP_6$		
$InsP_1$	1	1	1	0.05105	0.04681	0.04371	0.12857		
$InsP_2$									
$InsP_3$									
$InsP_4$									
$InsP_5$									
$InsP_6$									
R-$InsP_1$				0.01131	0.00944	0.00637	0.37333		
R-$InsP_2$				0.92078	0.92377	0.90794	0.49809		
R-$InsP_3$				0.01686	0.01997	0.02569			
R-$InsP_4$									
R-$InsP_5$									
R-$InsP_6$									

10.5. Dephosphorylation of phytate with 3-phytase from *Aspergillus niger* (10000 U/mL)

Supplementary Table 12 CE-qTOF-MS (CE-qTOF-MS (BGE: NH_4OAc 35 mM pH = 9.7, CE voltage: 30 kV, CE current: 23μA, injection: 100 mbar, 15 s (30 nL)) analysis digest of $InsP_6$ derivatives with 3-phytase from *Aspergillus niger* (10000 U/mL).

Phytase 21

	$InsP_6$			1-Fm-$InsP_6$			1-DEACM-$InsP_6$		
$InsP_1$	1	1	1	0.06391	0.06366	0.06560	0.06079	0.06406	0.06451
$InsP_2$									
$InsP_3$					0.00530	0.00726			
$InsP_4$									
$InsP_5$									
$InsP_6$									
R-$InsP_1$							0.34033	0.30993	0.33751
R-$InsP_2$				0.77362	0.74720	0.71100	0.59888	0.62603	0.59798
R-$InsP_3$				0.01532	0.01490	0.01496			
R-$InsP_4$				0.03641	0.03488	0.03663			
R-$InsP_5$									
R-$InsP_6$				0.11074	0.13406	0.16455			

11. NMR Spectra

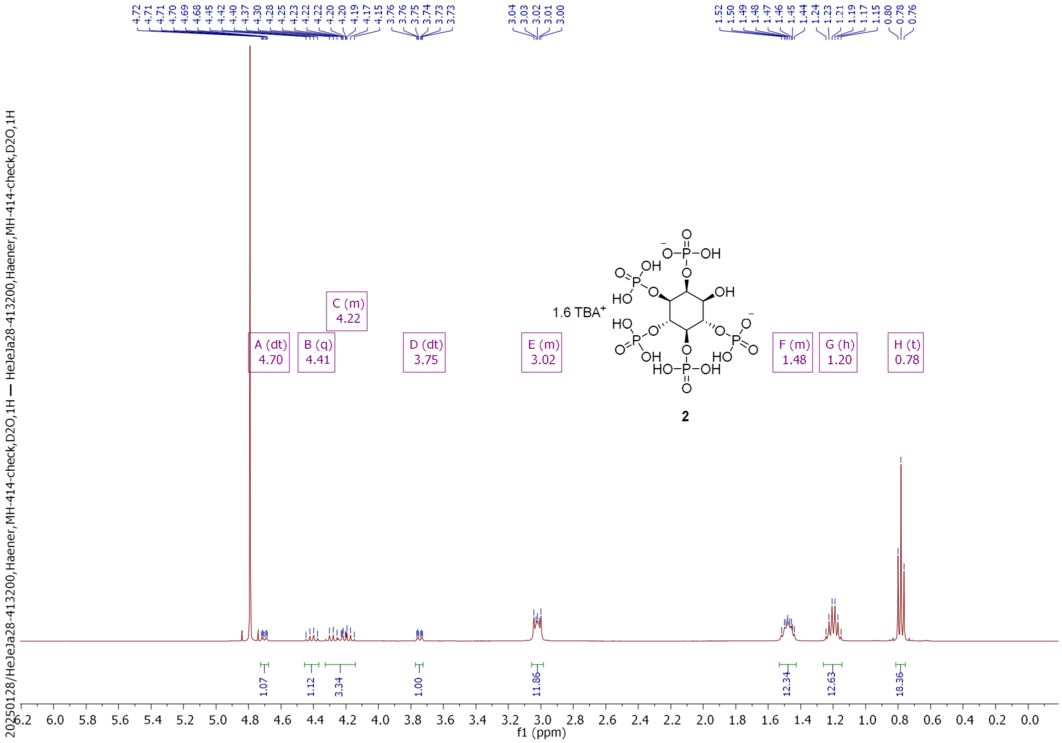

Supplementary Figure 16 ^{1}H-NMR of the TBA salt of 1-OH-$InsP_5$ **2**.

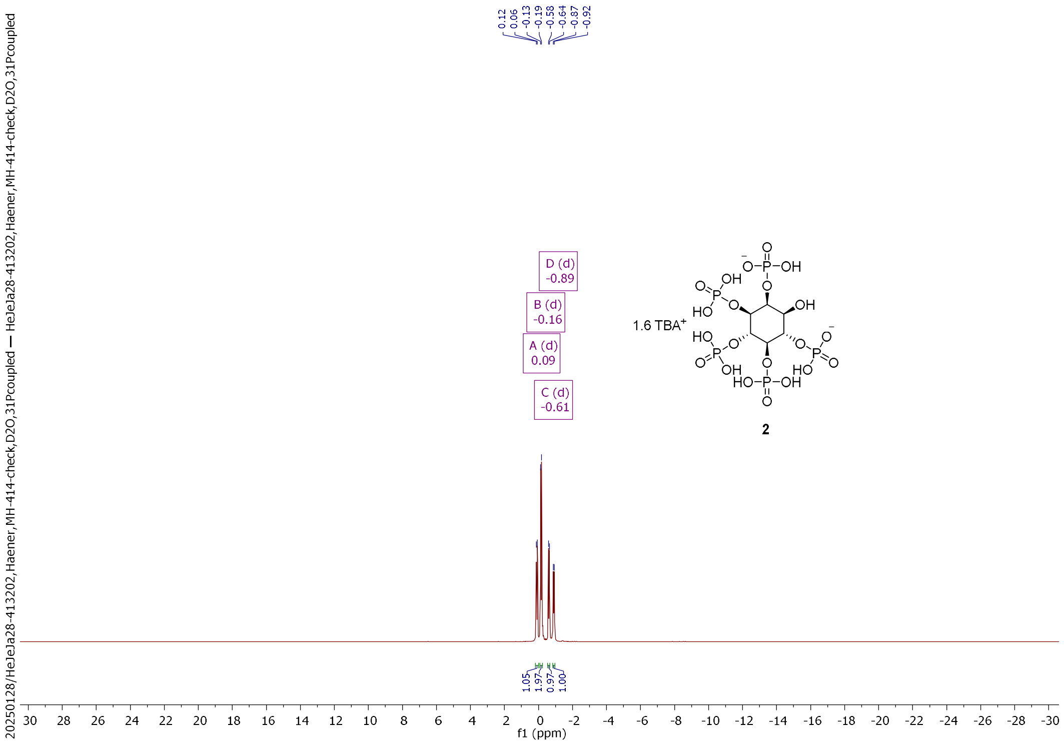

Supplementary Figure 17 ^{31}P-NMR of the TBA-salt of 1-OH-InsP$_5$ **2**.

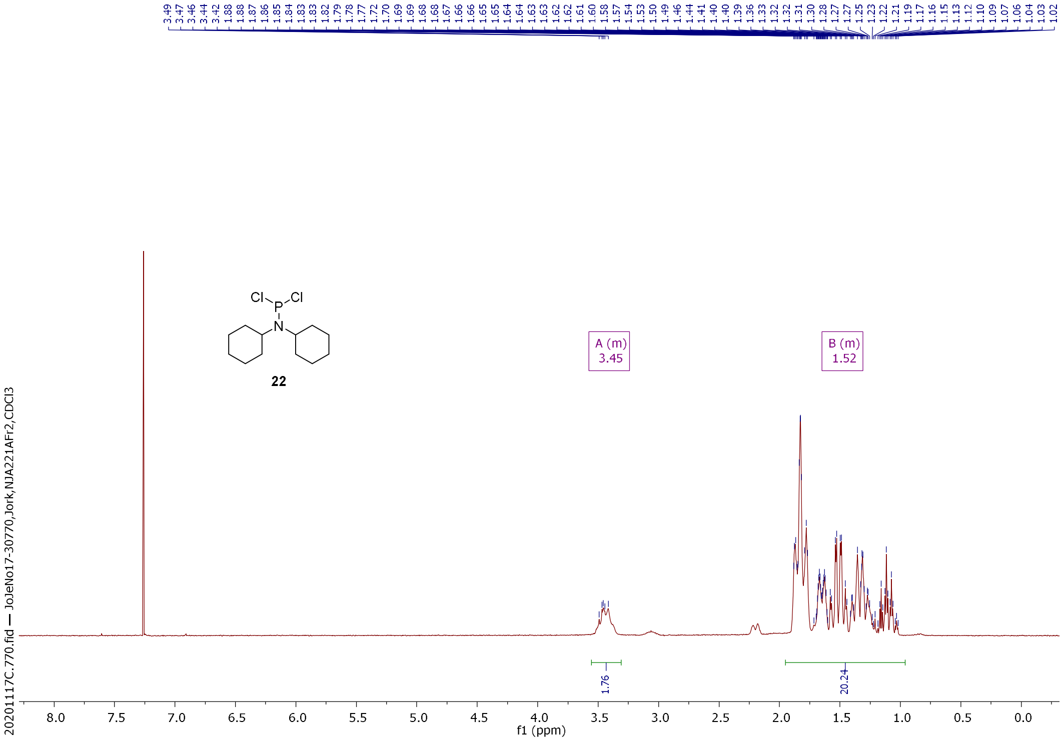

Supplementary Figure 18 ^{1}H-NMR of **22**.

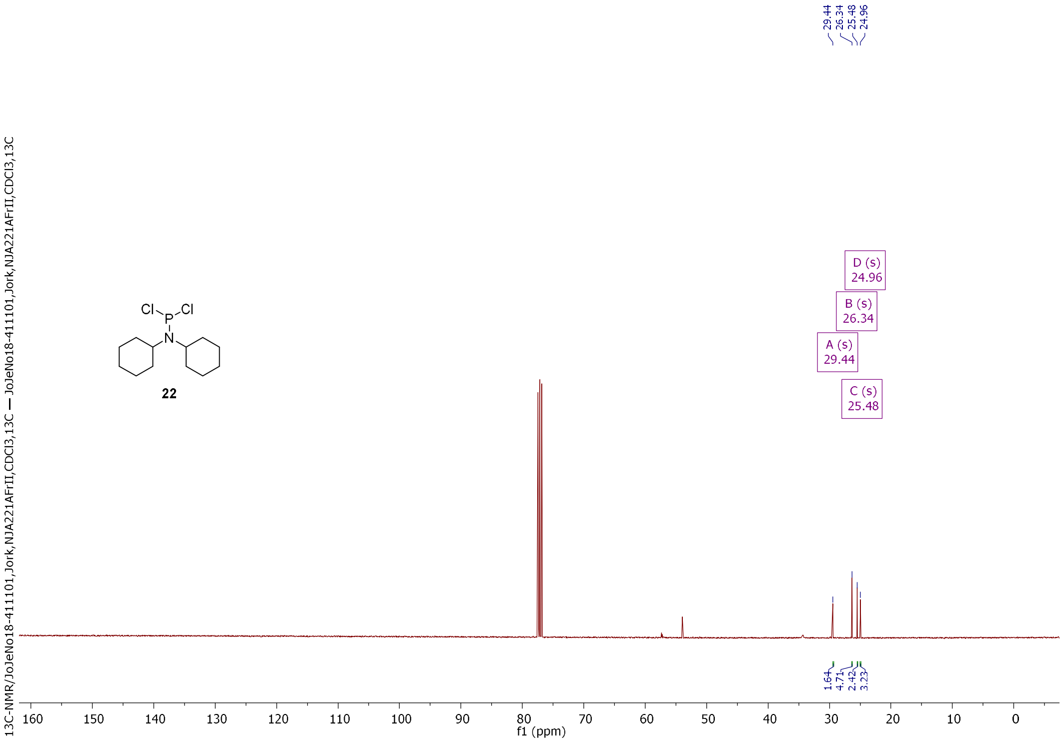

Supplementary Figure 19 ^{13}C-NMR of **22**.

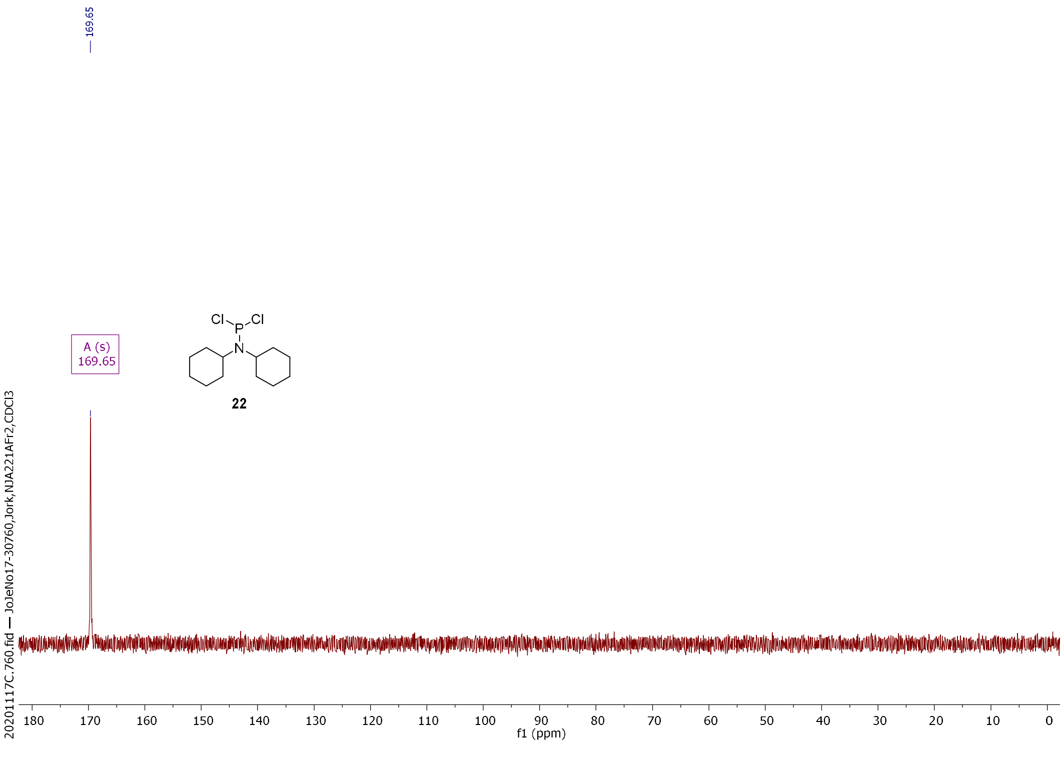

Supplementary Figure 20 ^{31}P{^{1}H}-NMR of **22**.

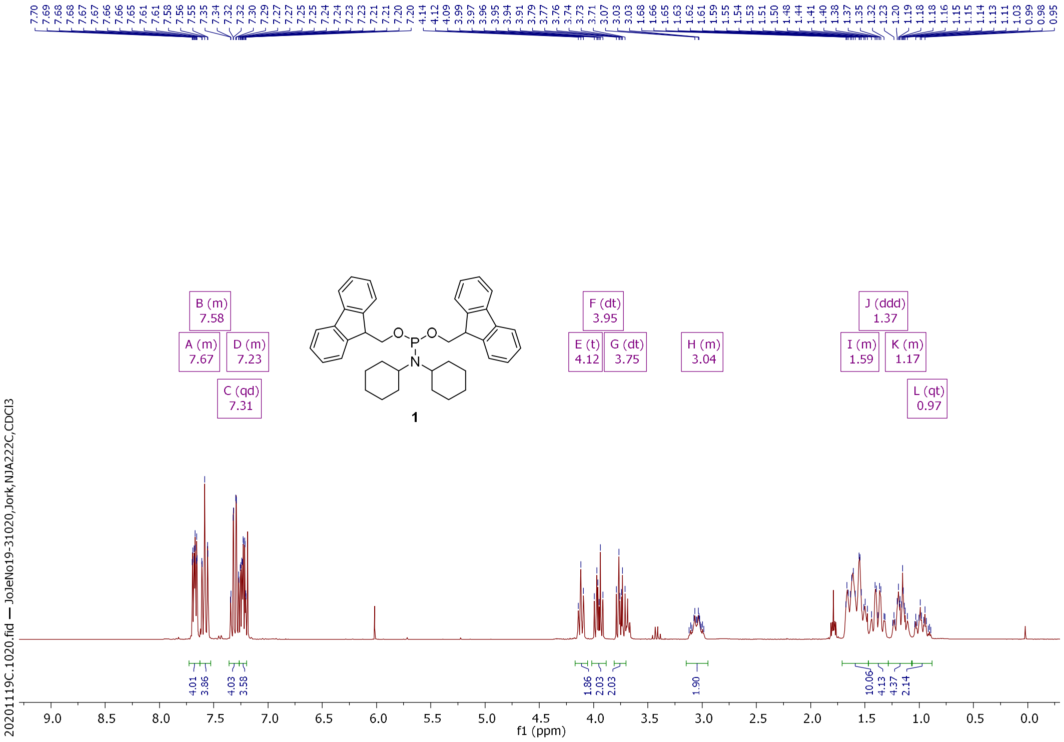

Supplementary Figure 21 ^{1}H-NMR of **1**.

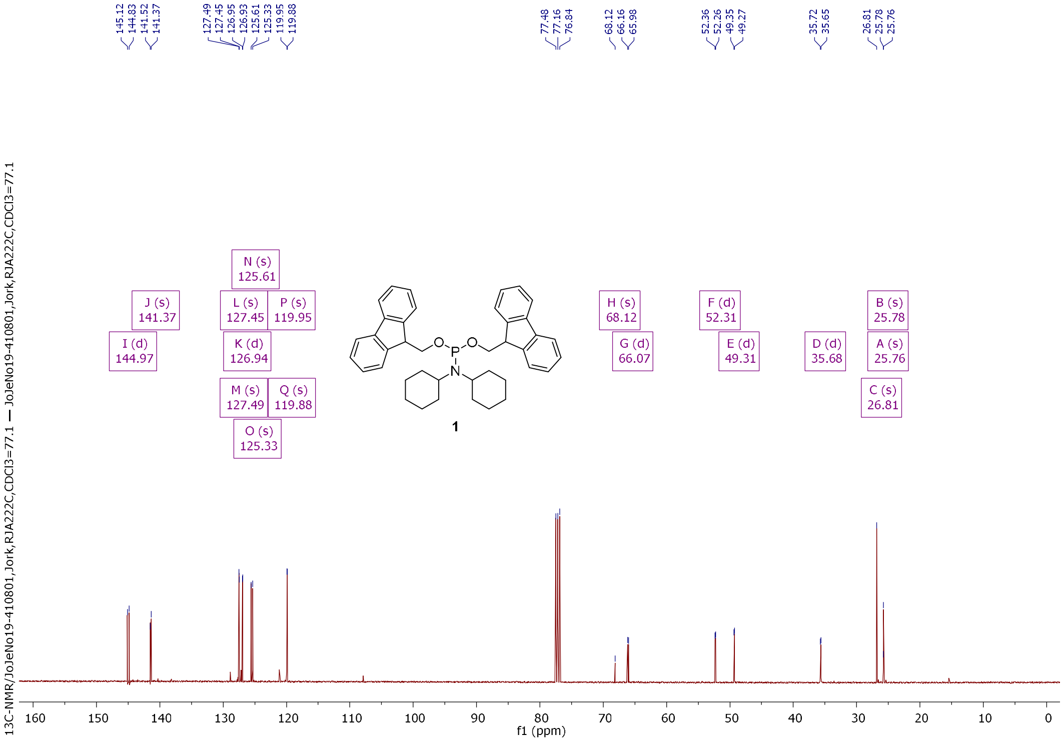

Supplementary Figure 22 ^{13}C-NMR of **1**.

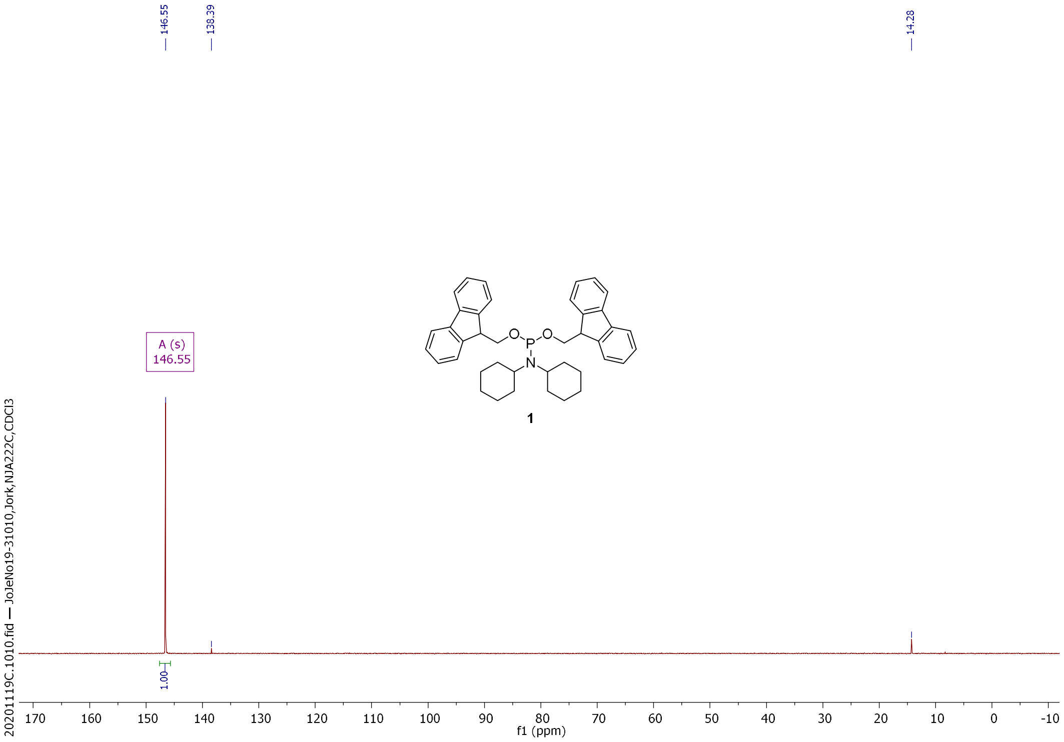

Supplementary Figure 23 $^{31}P\{^{1}H\}$-NMR of **1**.

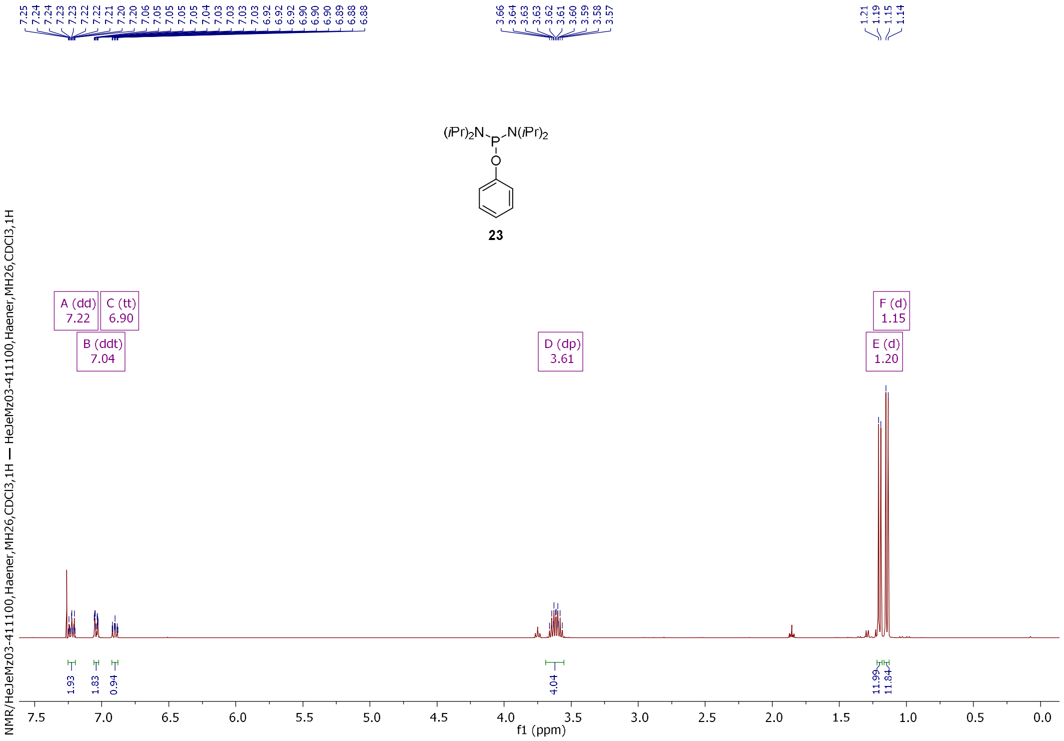

Supplementary Figure 24 ^{1}H-NMR of **23**.

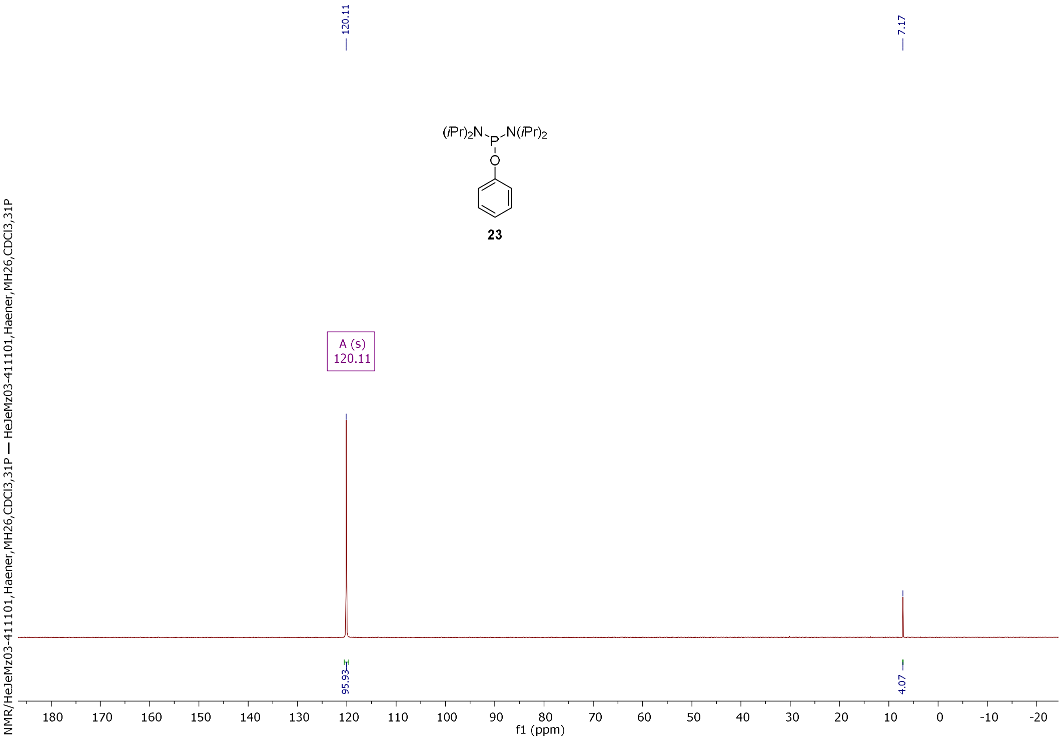

Supplementary Figure 25 $^{31}P\{^{1}H\}$-NMR of **23**.

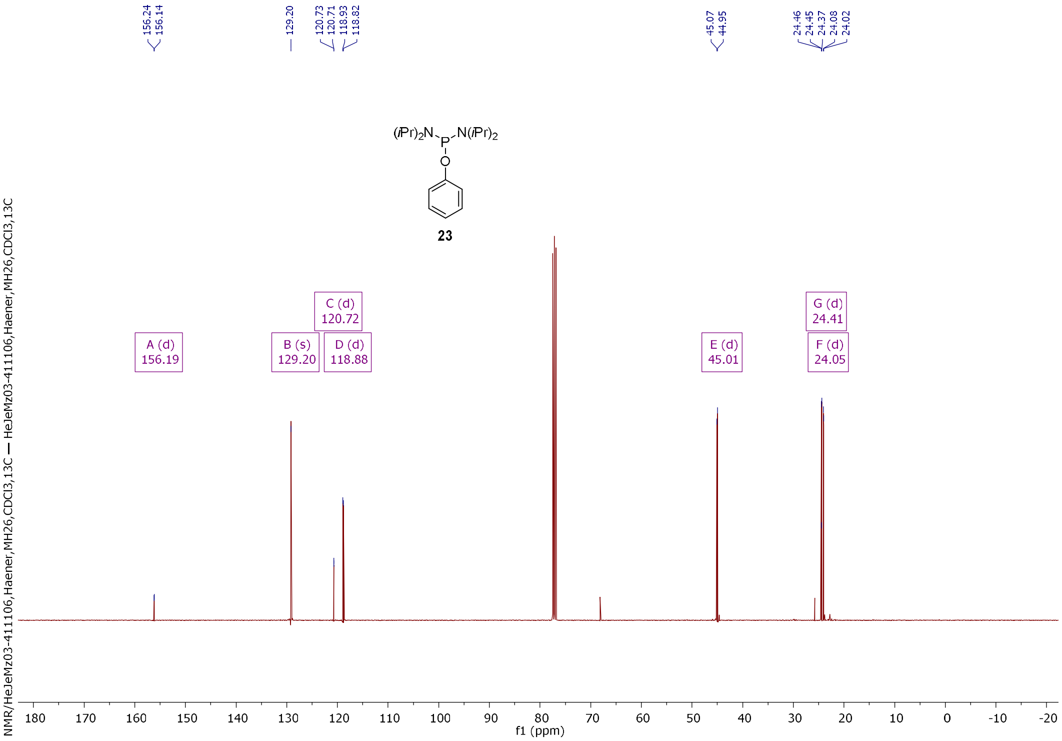

Supplementary Figure 26 ^{13}C-NMR of **23**.

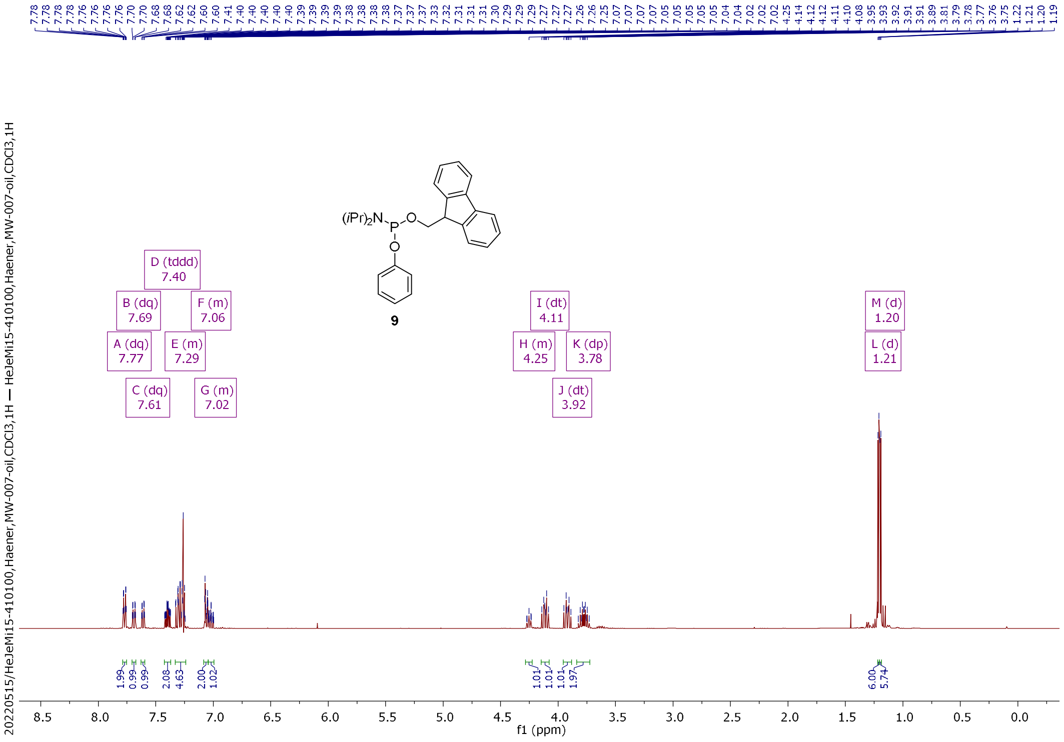

Supplementary Figure 27 ^{1}H-NMR of **9**.

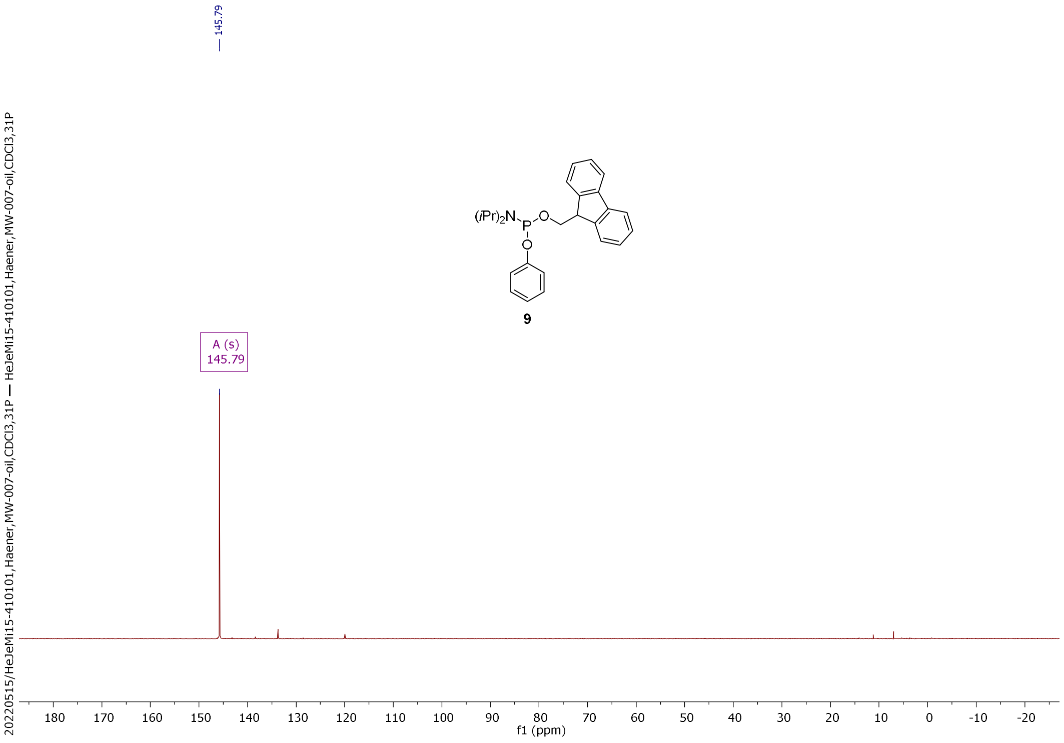

Supplementary Figure 28 ^{31}P{^{1}H}-NMR of **9**.

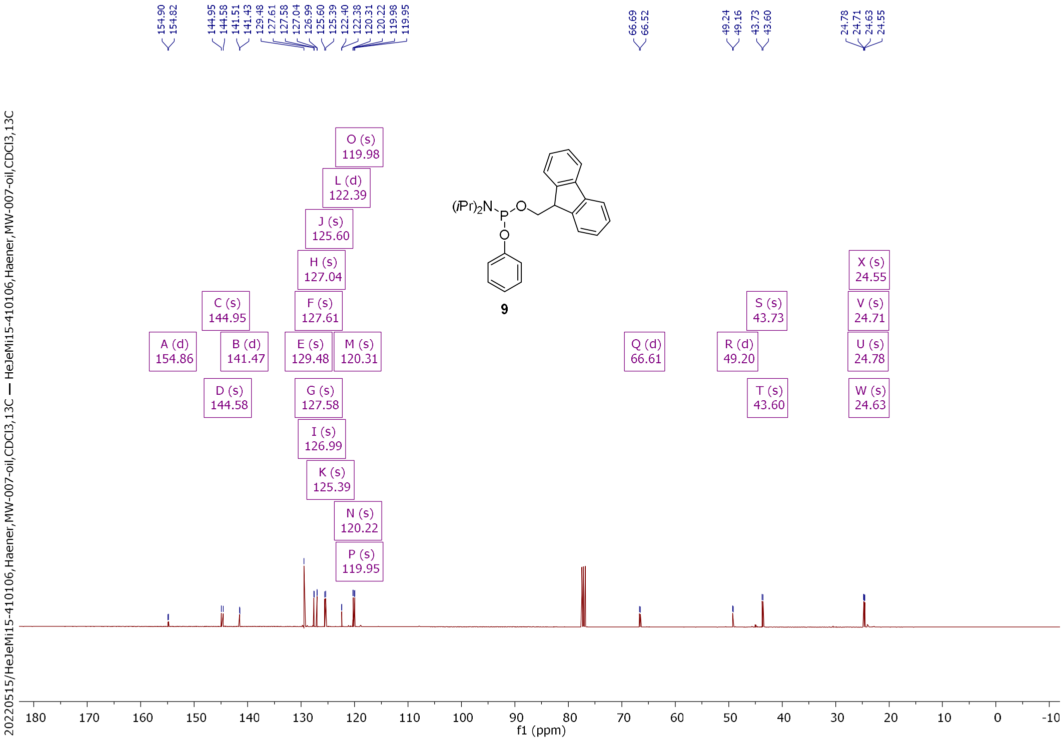

Supplementary Figure 29 ^{13}C-NMR of **9**.

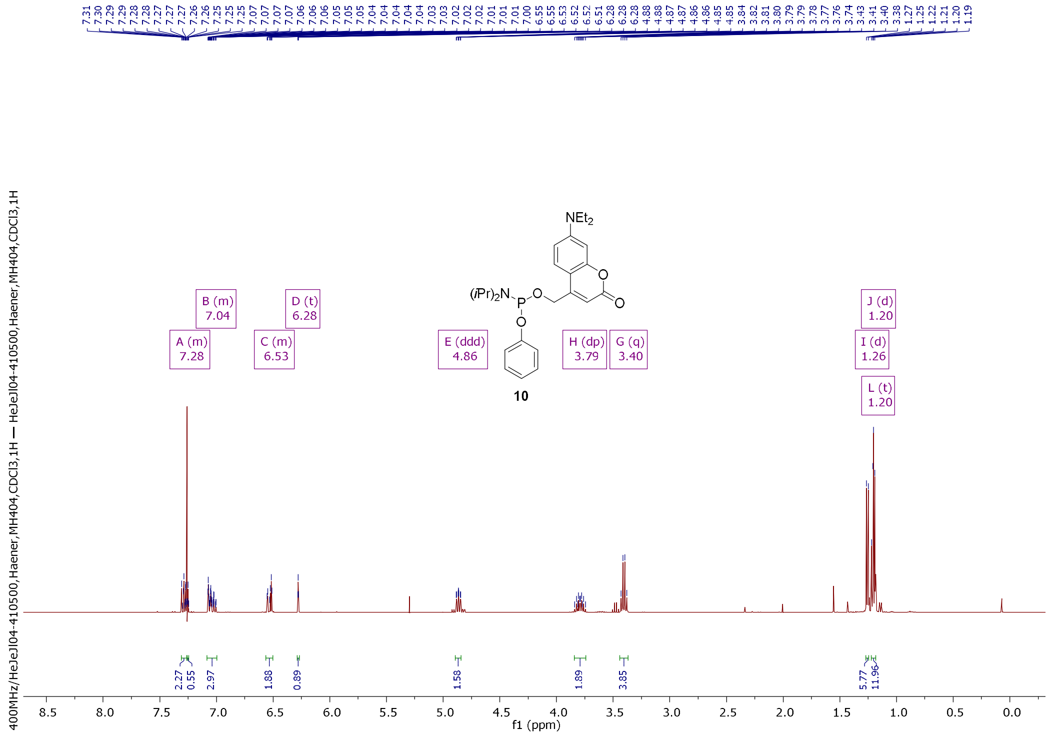

Supplementary Figure 30 ^{1}H-NMR of **10**.

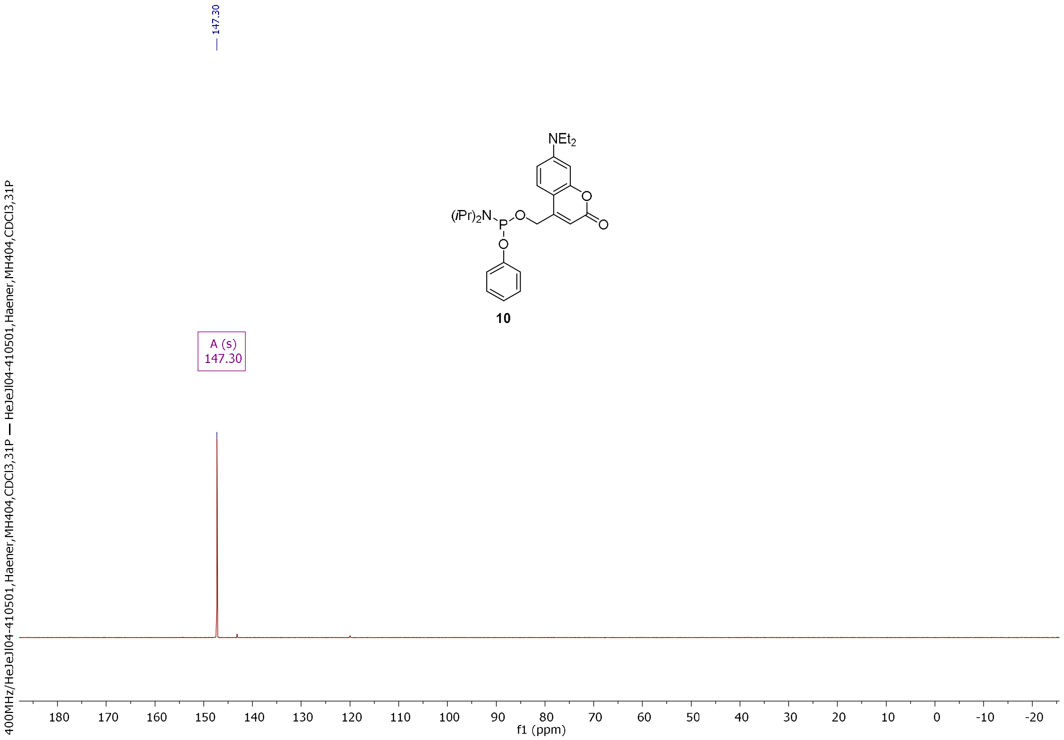

Supplementary Figure 31 $^{31}P\{^{1}H\}$-NMR of **10**.

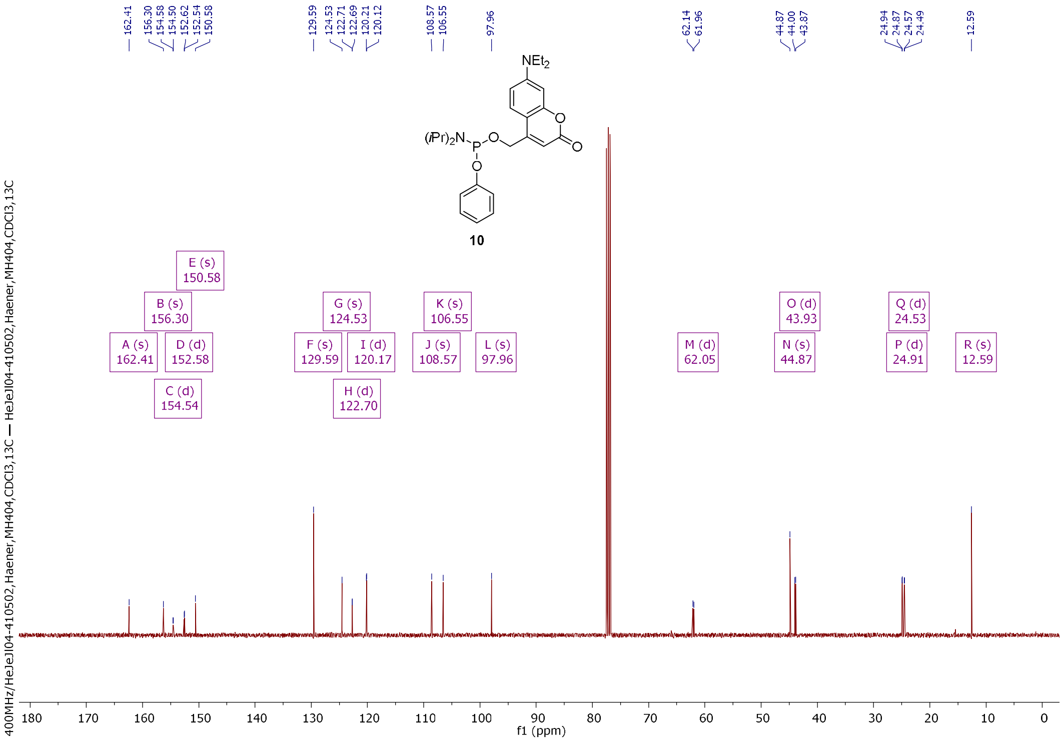

Supplementary Figure 32 ^{13}C-NMR of **10**.

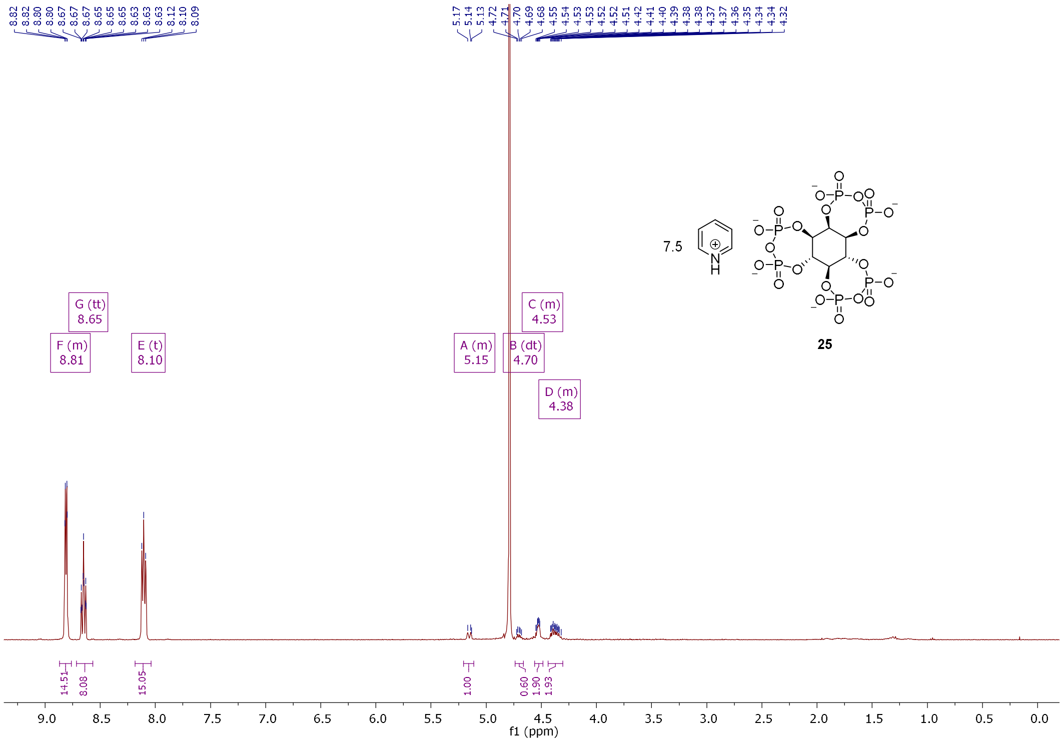

Supplementary Figure 33 ^{1}H-NMR of **25**.

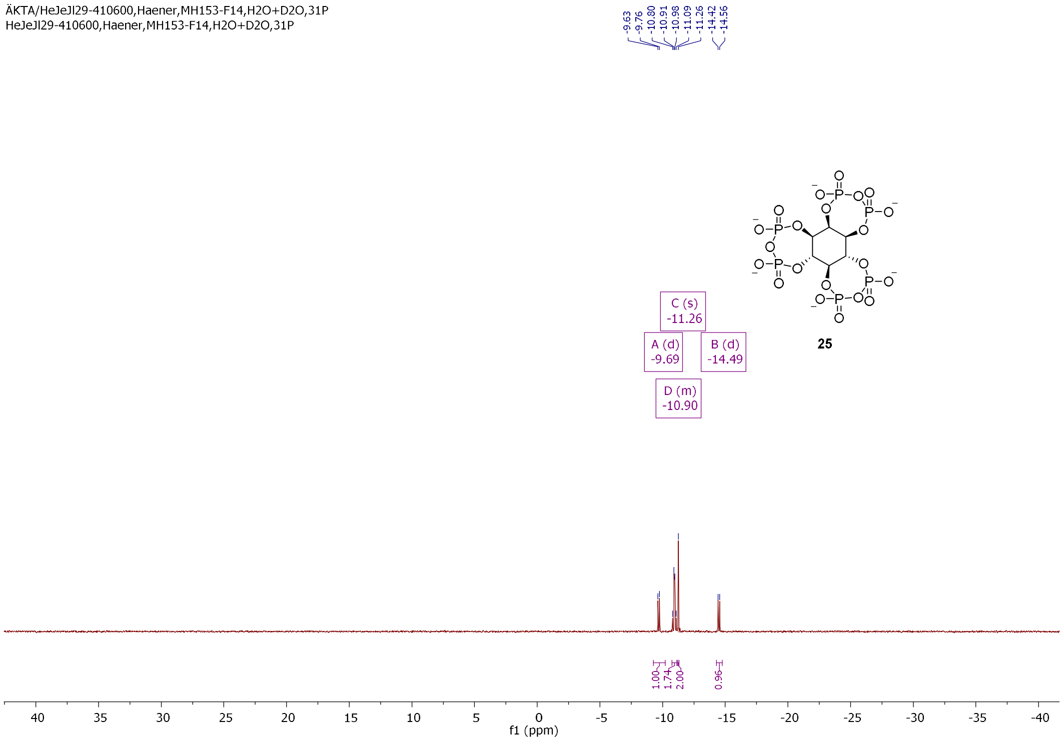

Supplementary Figure 34 $^{31}P\{^{1}H\}$-NMR of **25**.

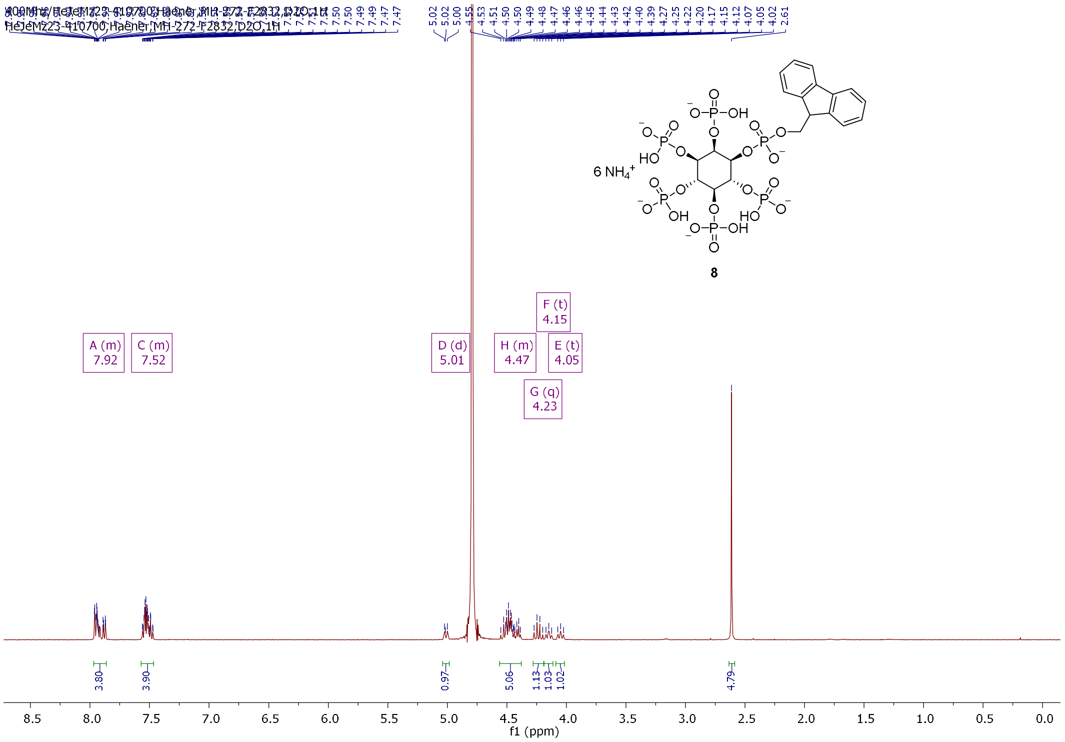

Supplementary Figure 35 ^{1}H-NMR of **8**.

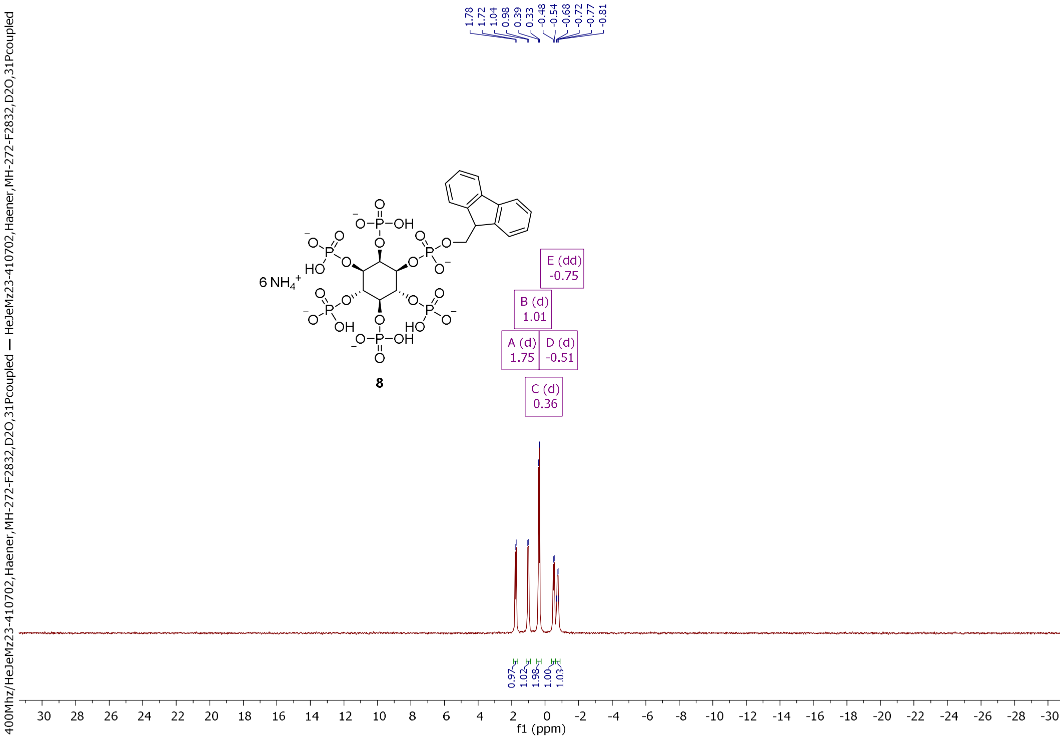

Supplementary Figure 36 ^{31}P-NMR of **8**.

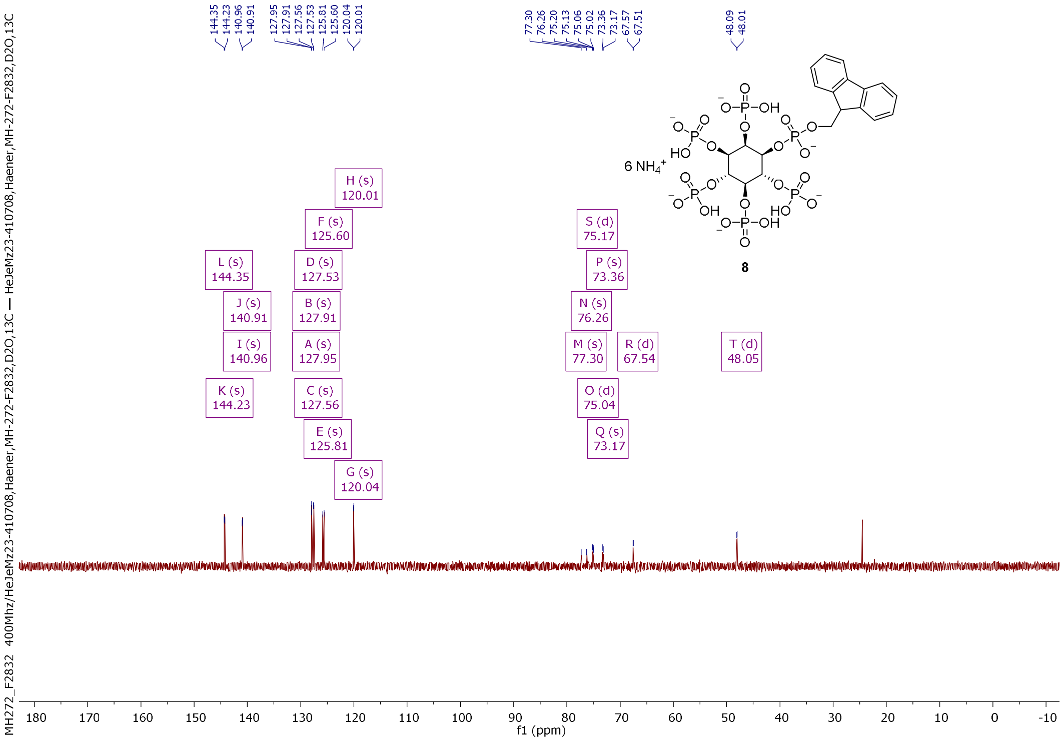

Supplementary Figure 37 ^{13}C-NMR of **8**.

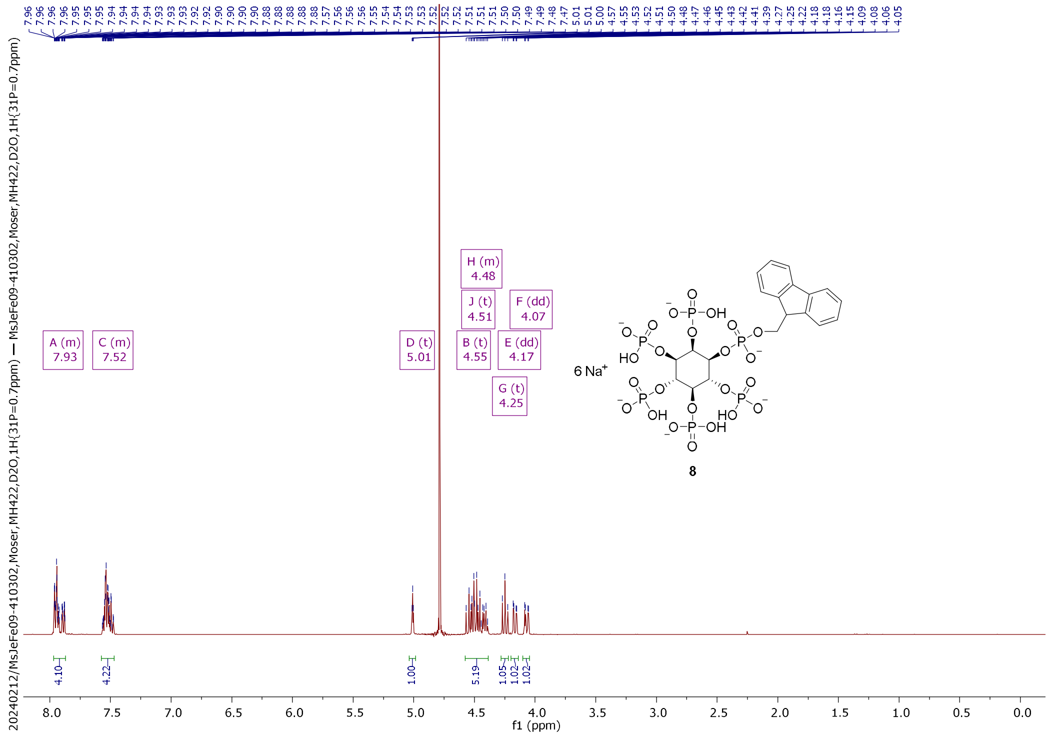

Supplementary Figure 38 ^{1}H-NMR of **8**.

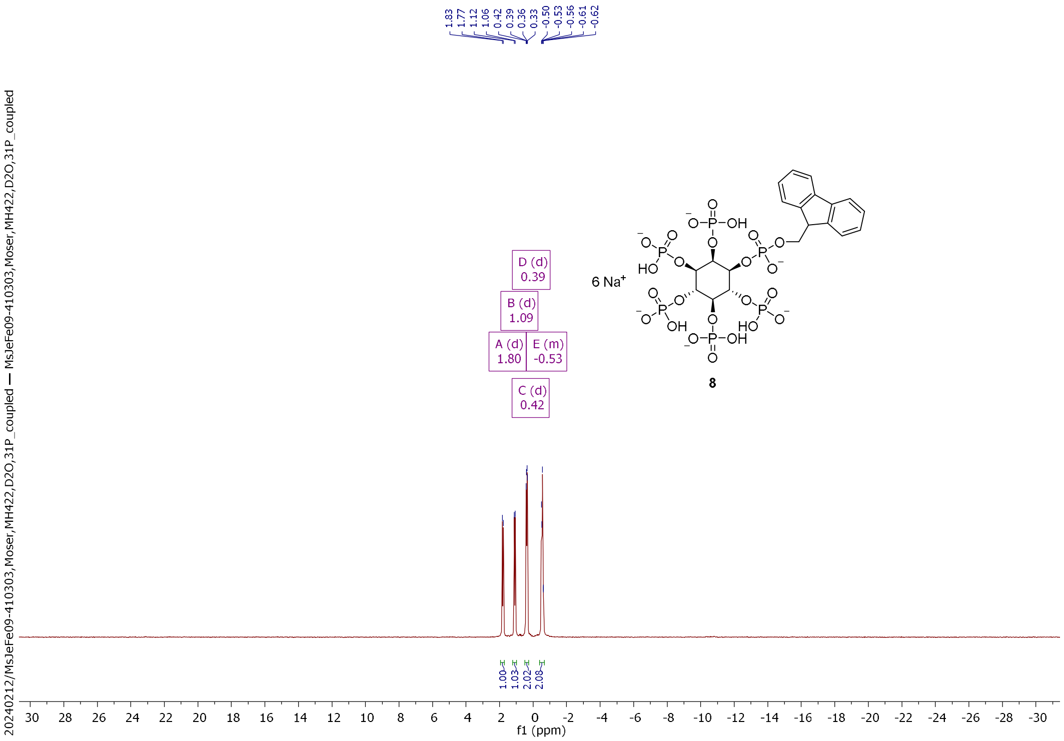

Supplementary Figure 39 ^{31}P-NMR of **8**.

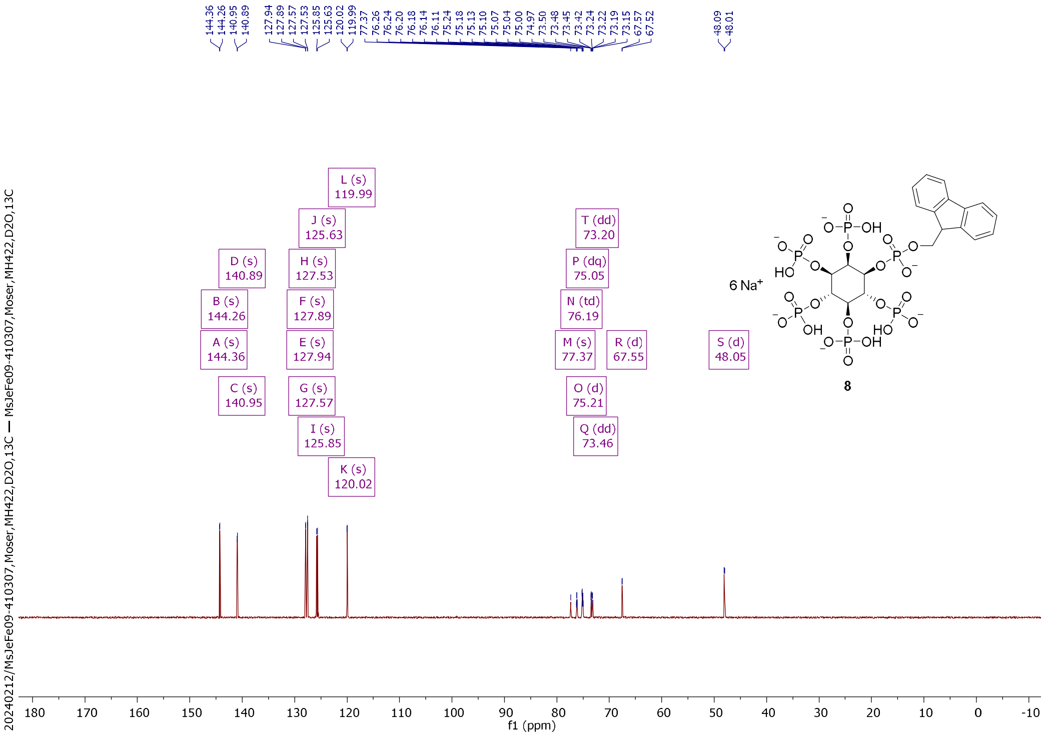

Supplementary Figure 40 ^{13}C-NMR of **8**.

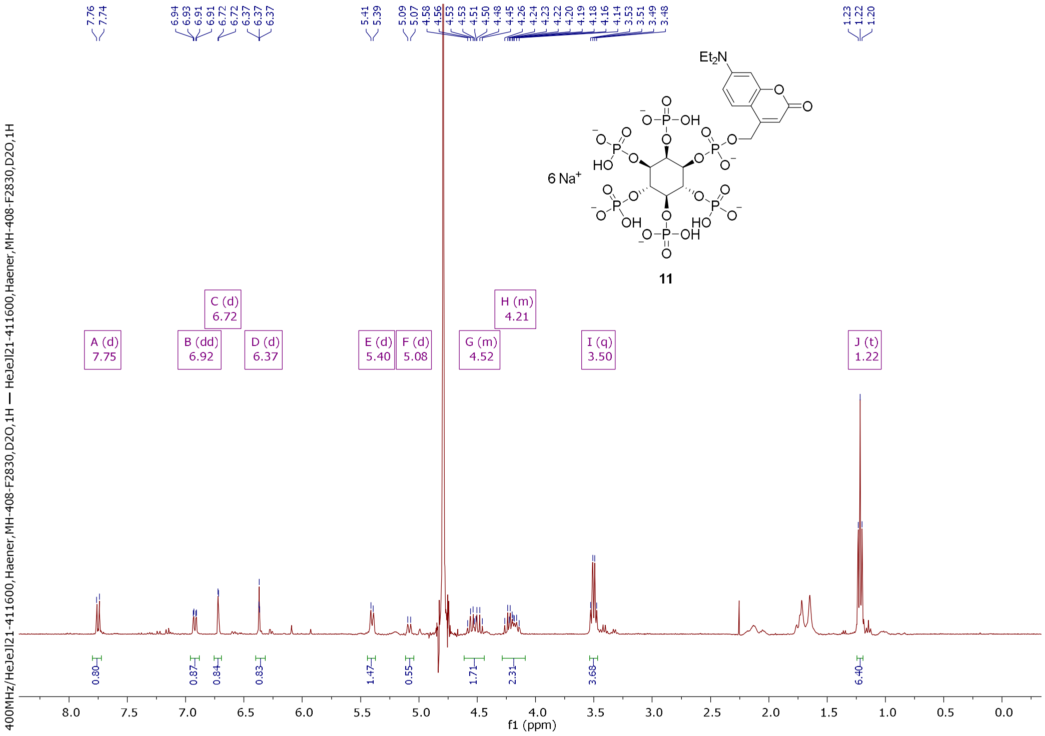

Supplementary Figure 41 ^{1}H-NMR of **11**.

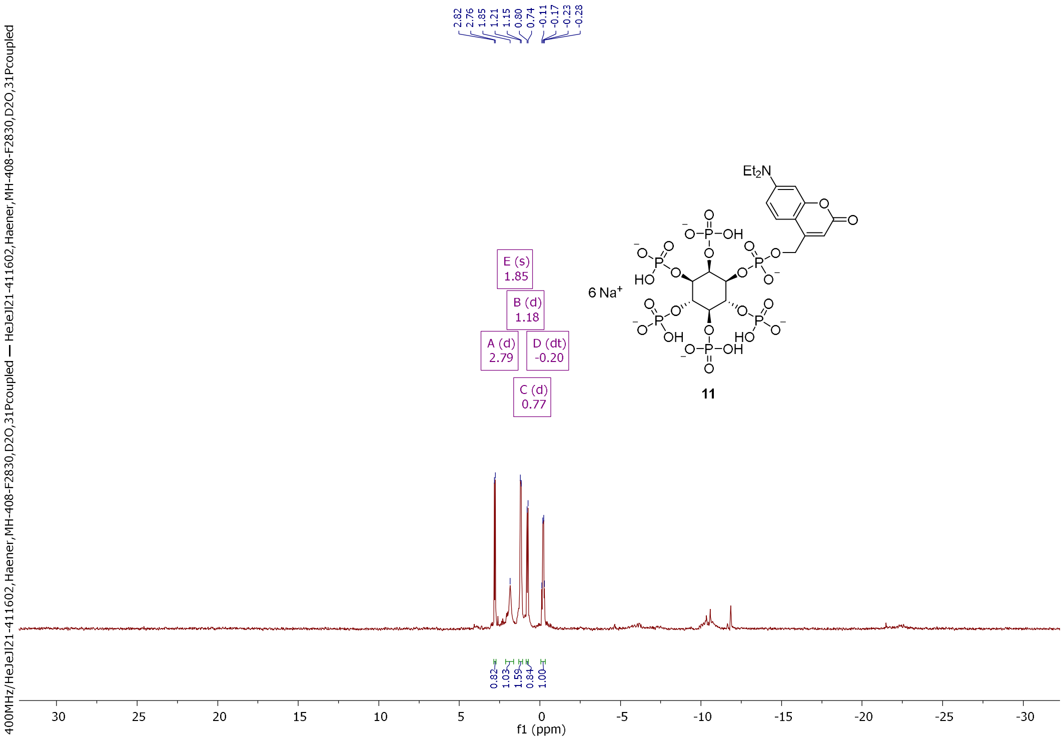

Supplementary Figure 42 ^{31}P-NMR of **11**.

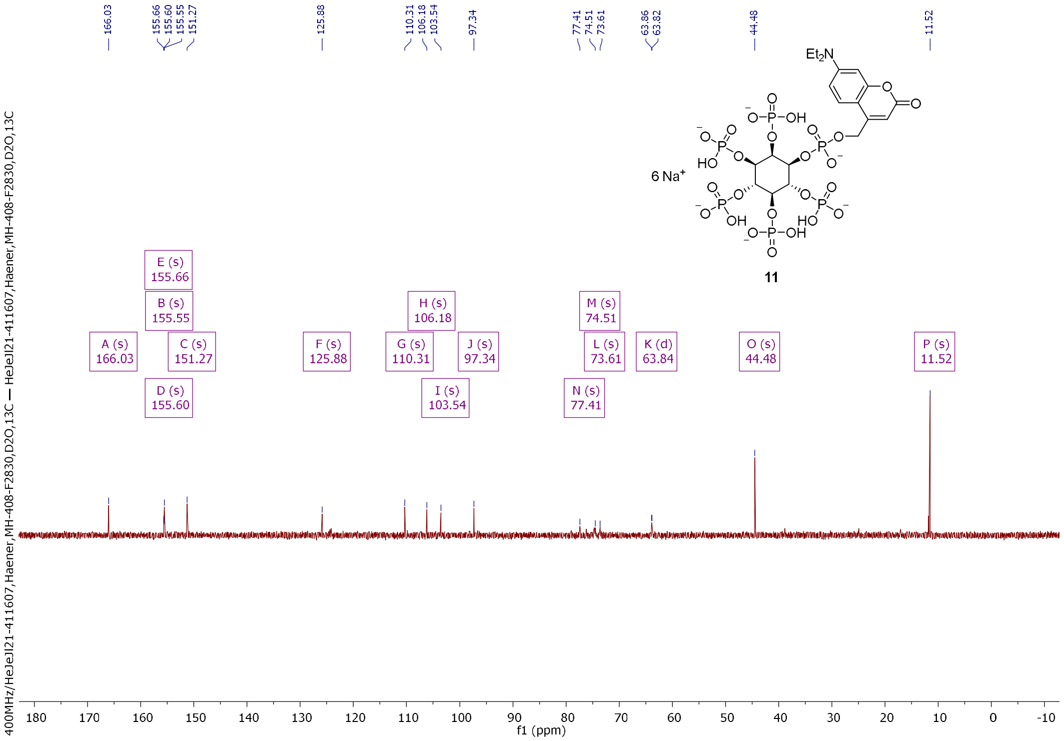

Supplementary Figure 43 ^{13}C-NMR of **11**.

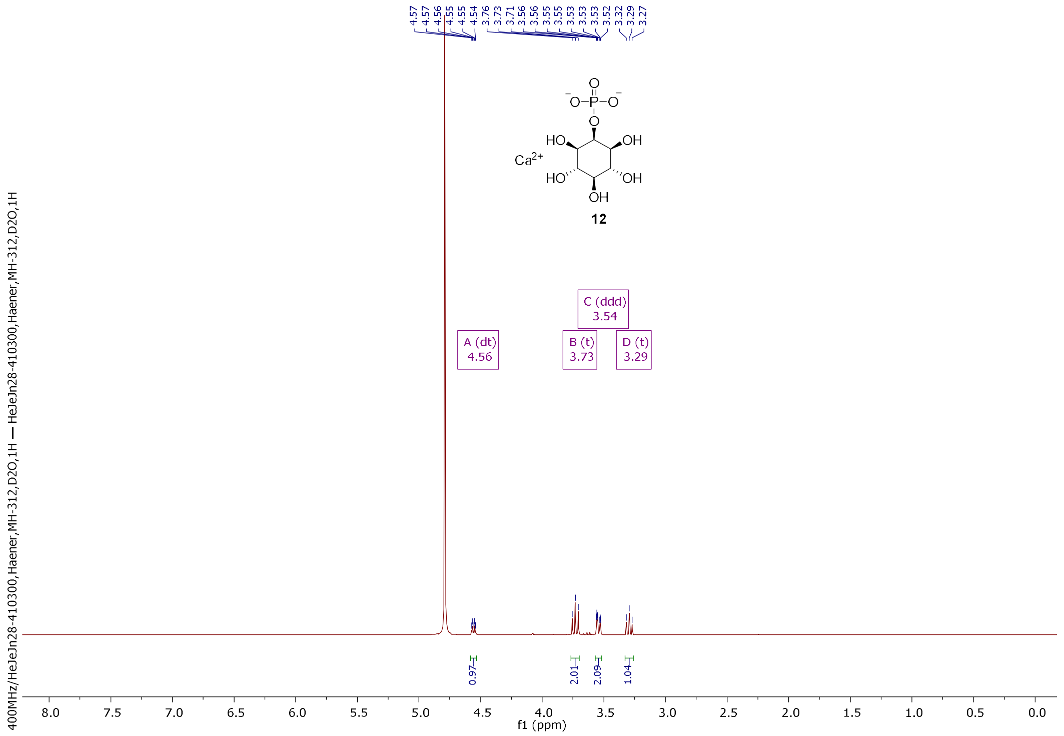

Supplementary Figure 44 ^{1}H-NMR of **12**.

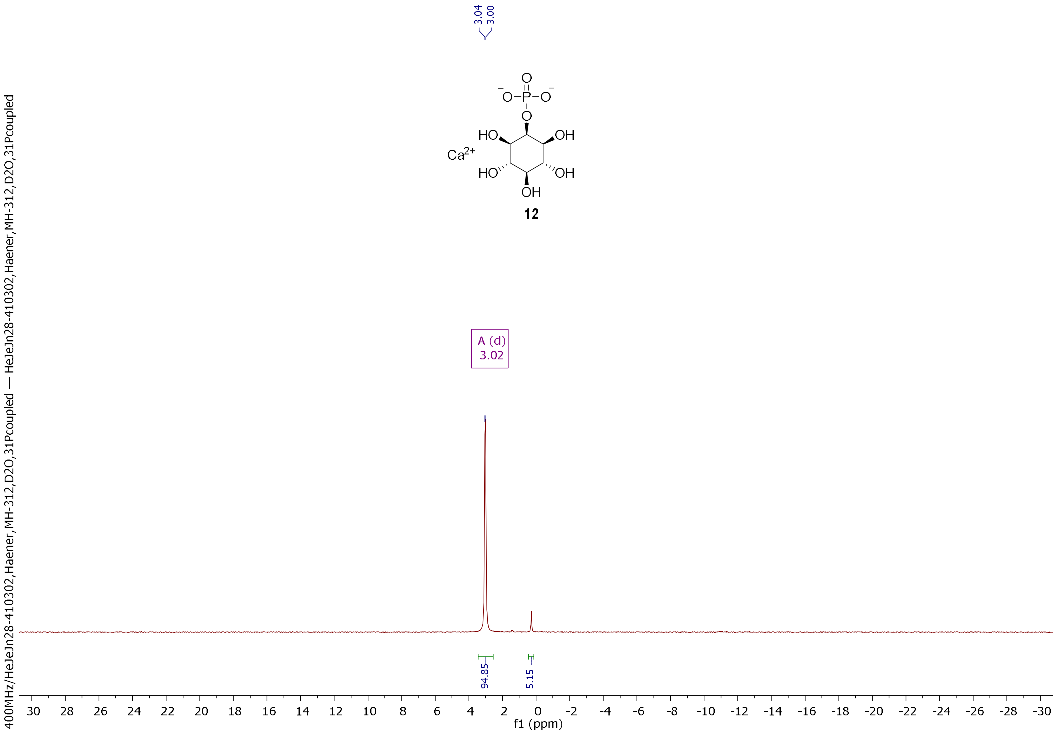

Supplementary Figure 45 ^{31}P-NMR of **12**.

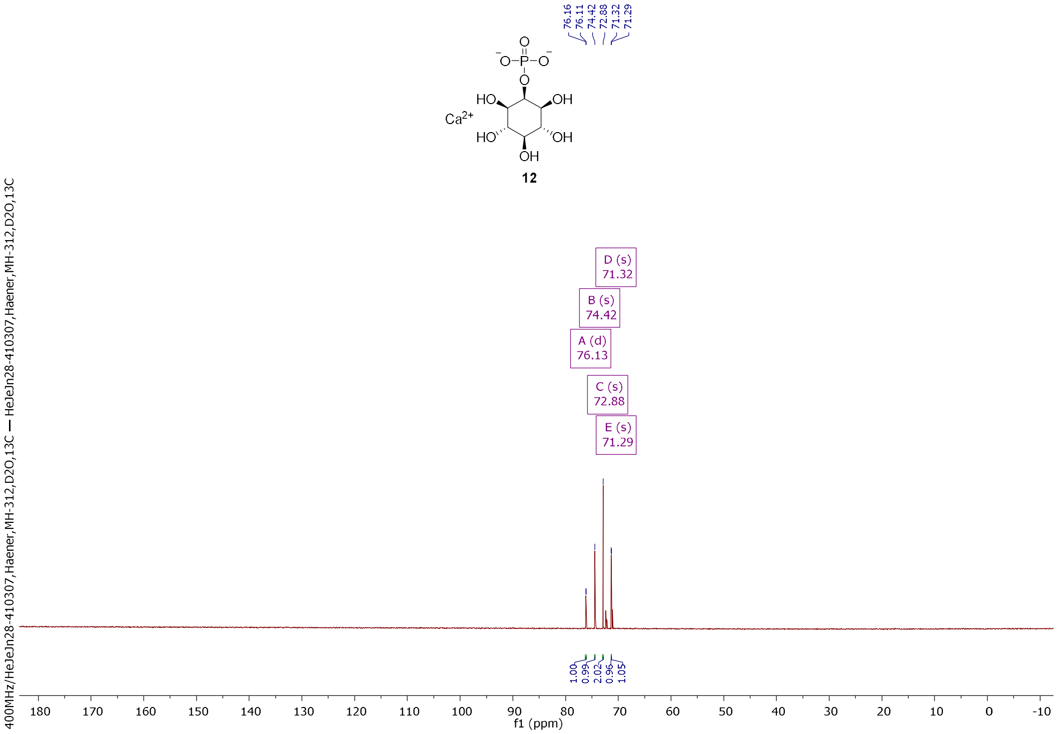

Supplementary Figure 46 ^{13}C-NMR of **12**.

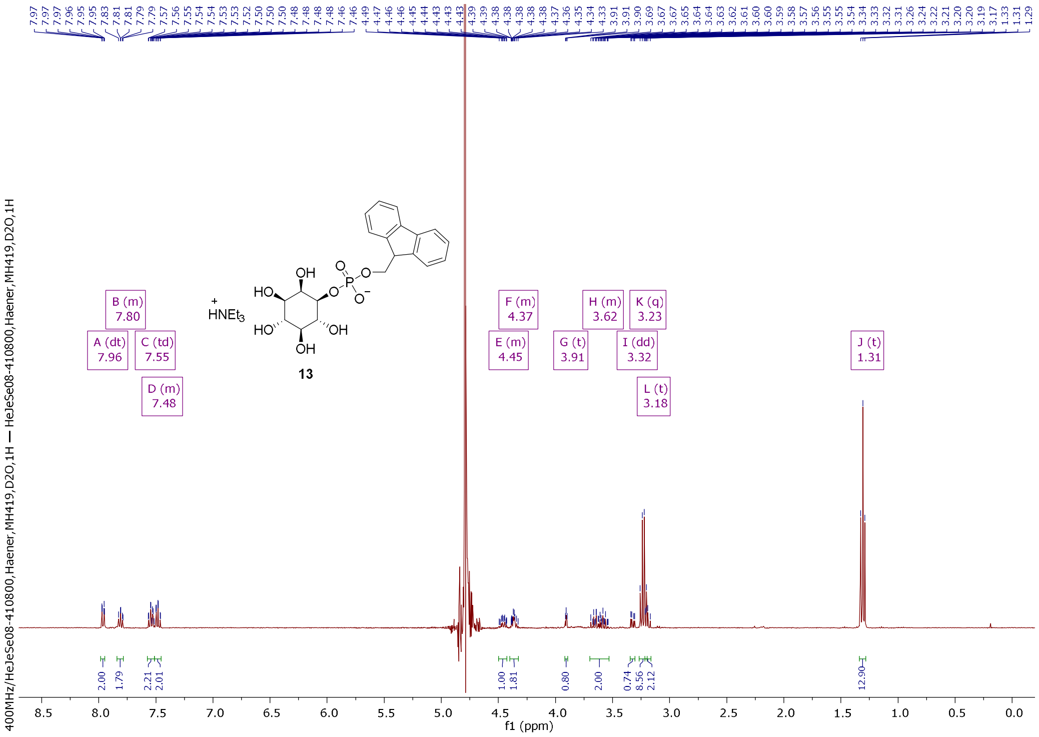

Supplementary Figure 47 ^{1}H-NMR of **13**.

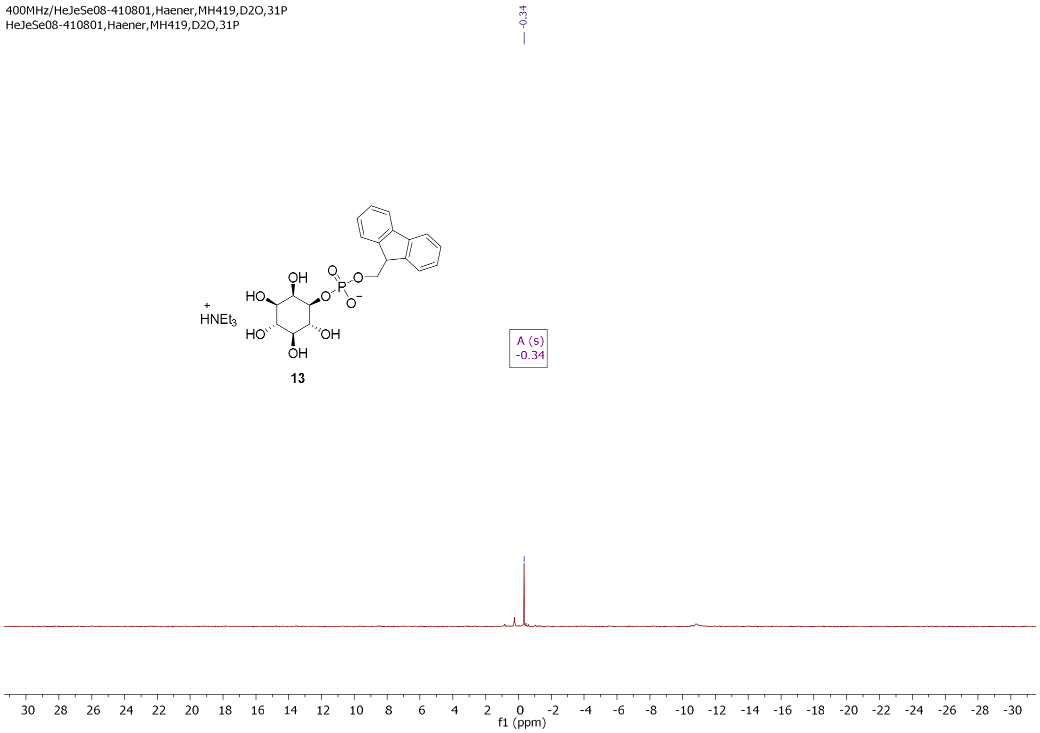

Supplementary Figure 48 $^{31}P\{^{1}H\}$-NMR of **13**.

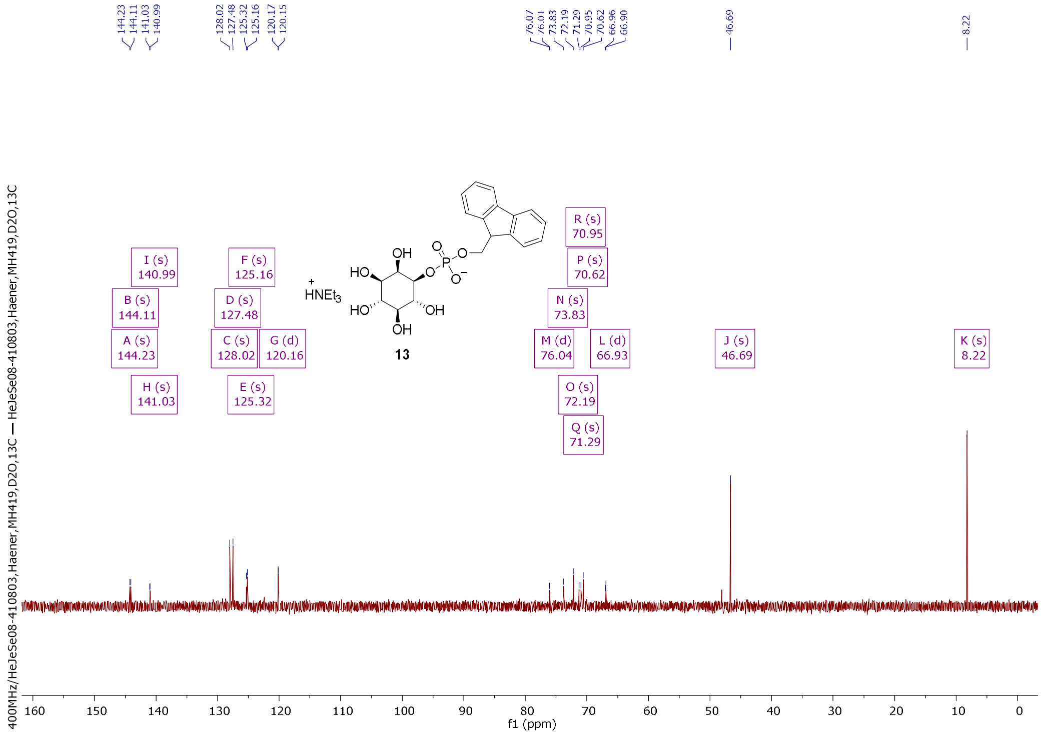

Supplementary Figure 49 ^{13}C-NMR of **13**.

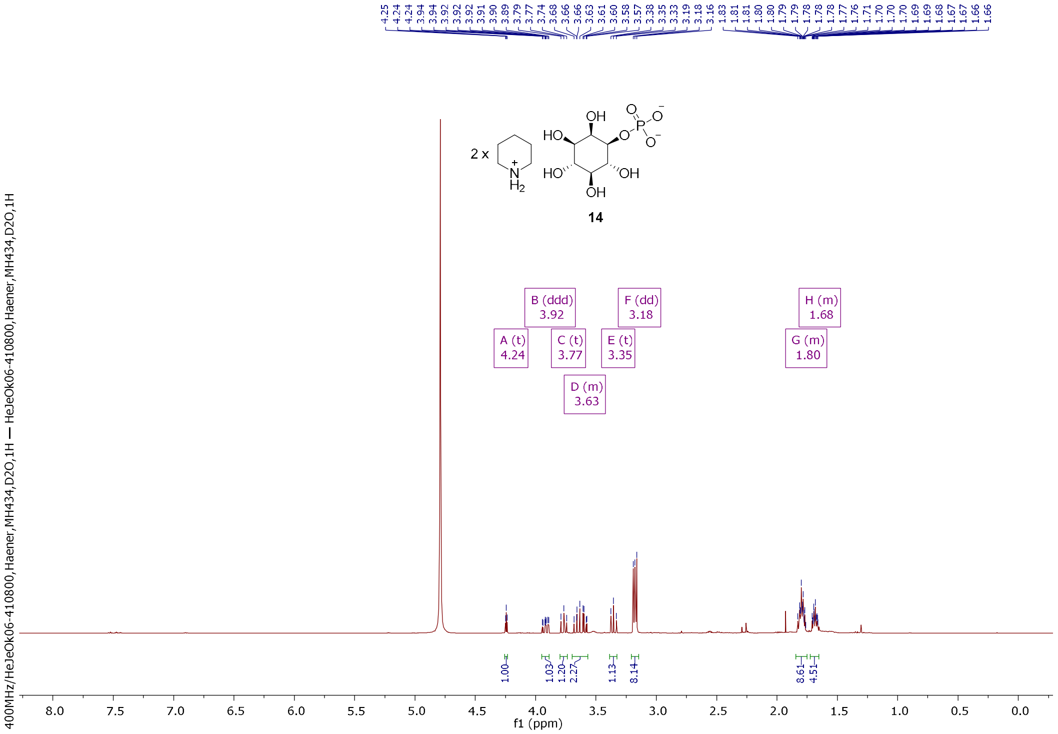

Supplementary Figure 50 ^{1}H-NMR of **14**.

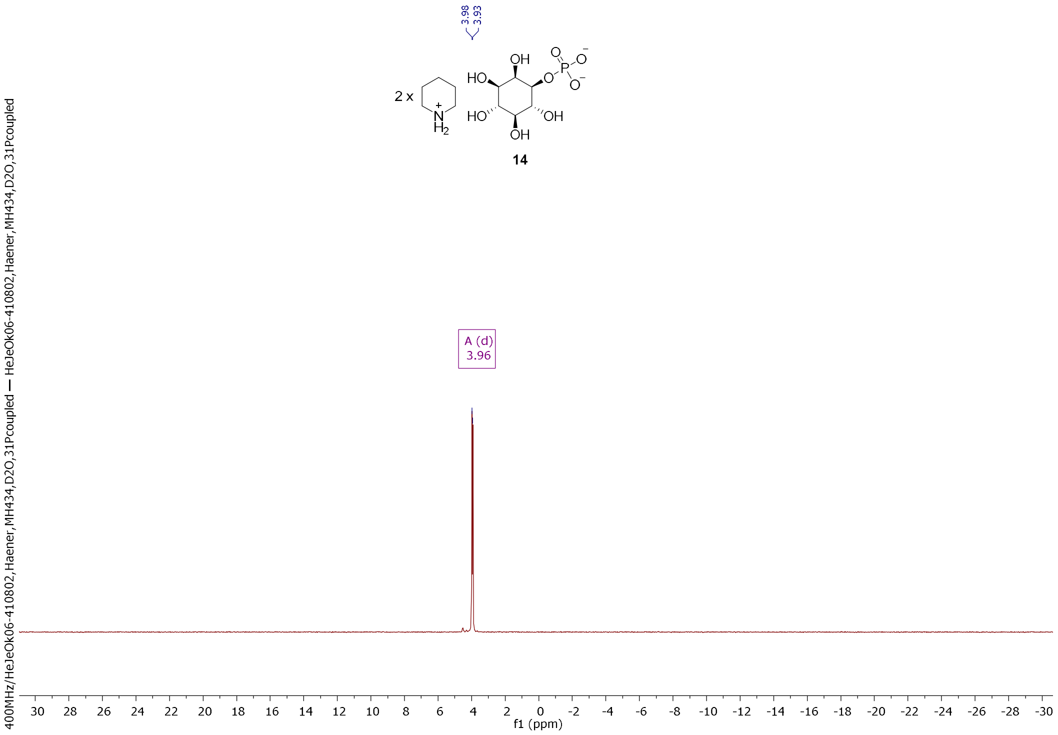

Supplementary Figure 51 ^{31}P-NMR of **14**.

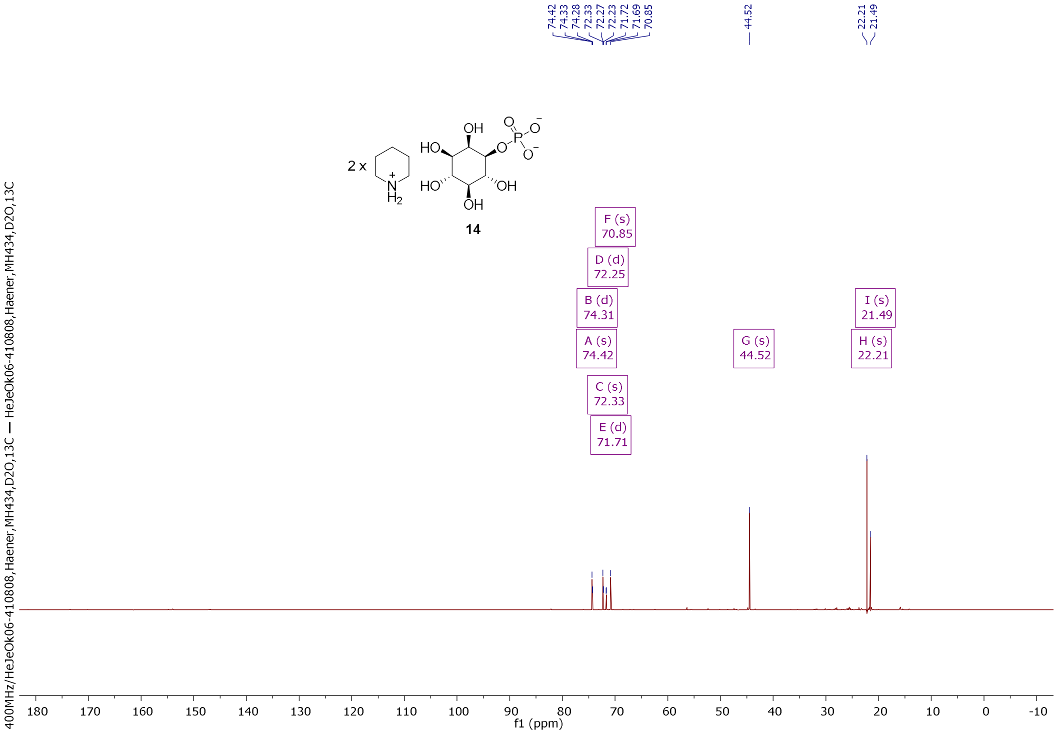

Supplementary Figure 52 ^{13}C-NMR of **14**.

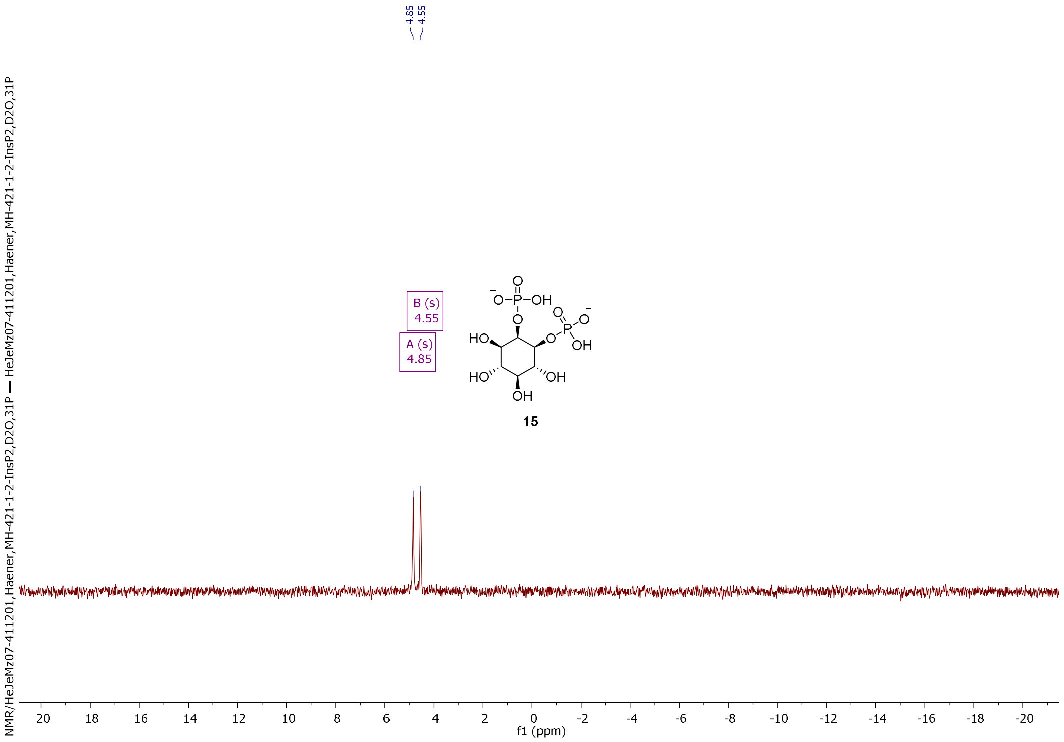

Supplementary Figure 53 $^{31}P\{^1H\}$-NMR of **15**.

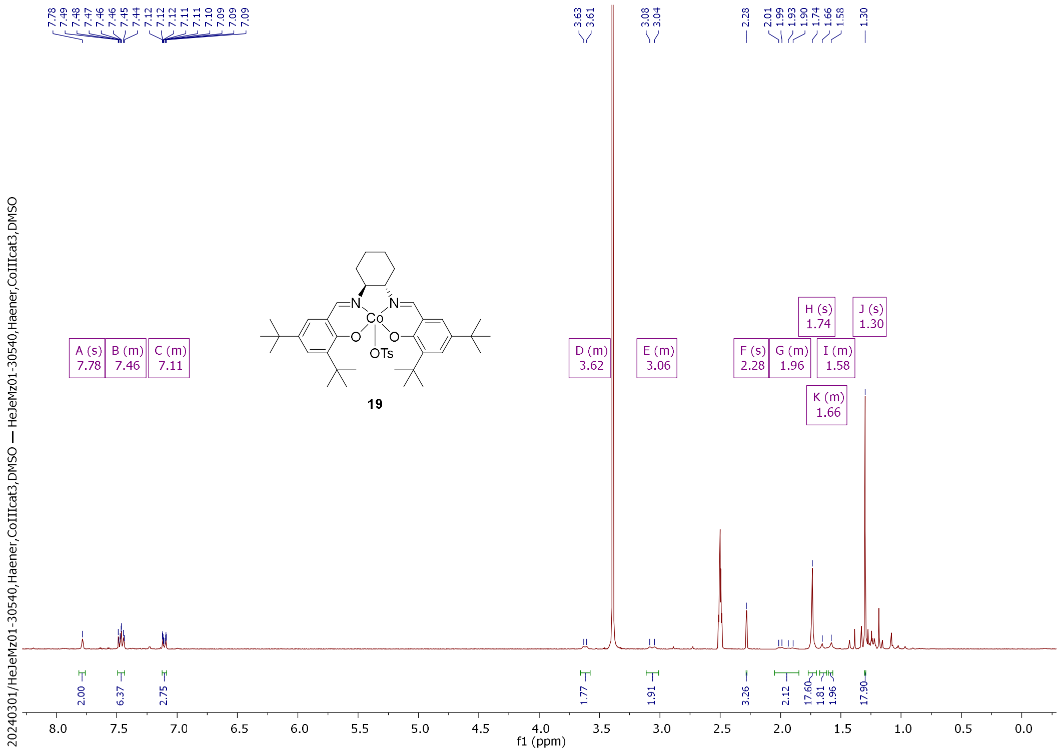

Supplementary Figure 54 ^1H-NMR of **19**.

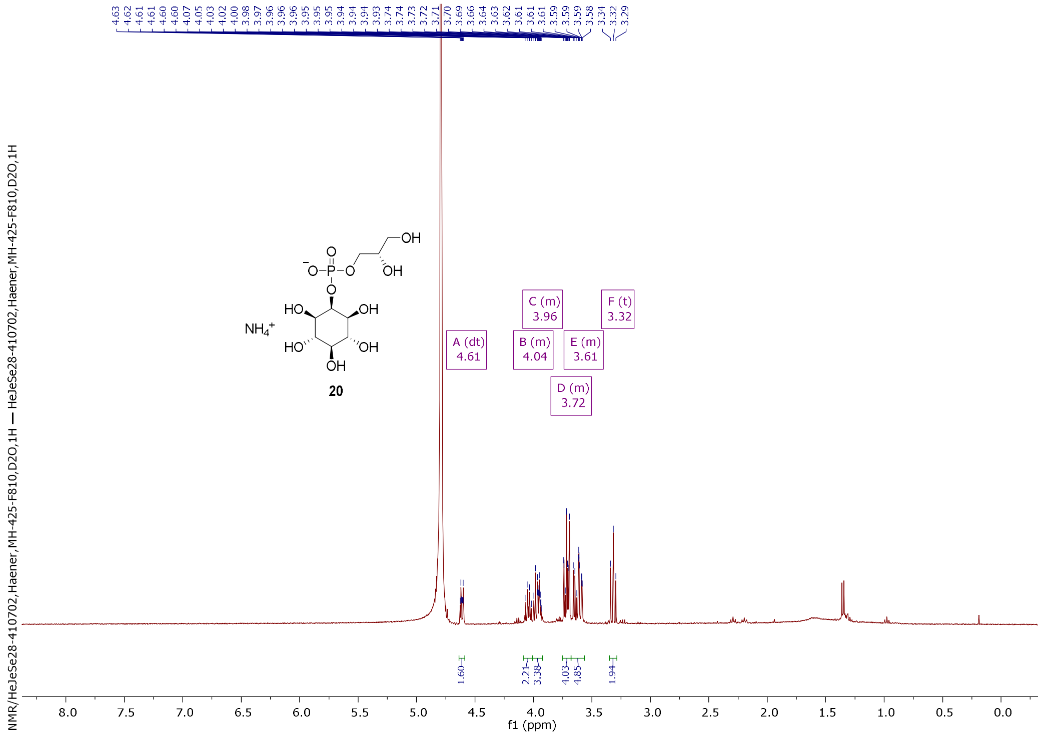

Supplementary Figure 55 ^{1}H-NMR of **20**.

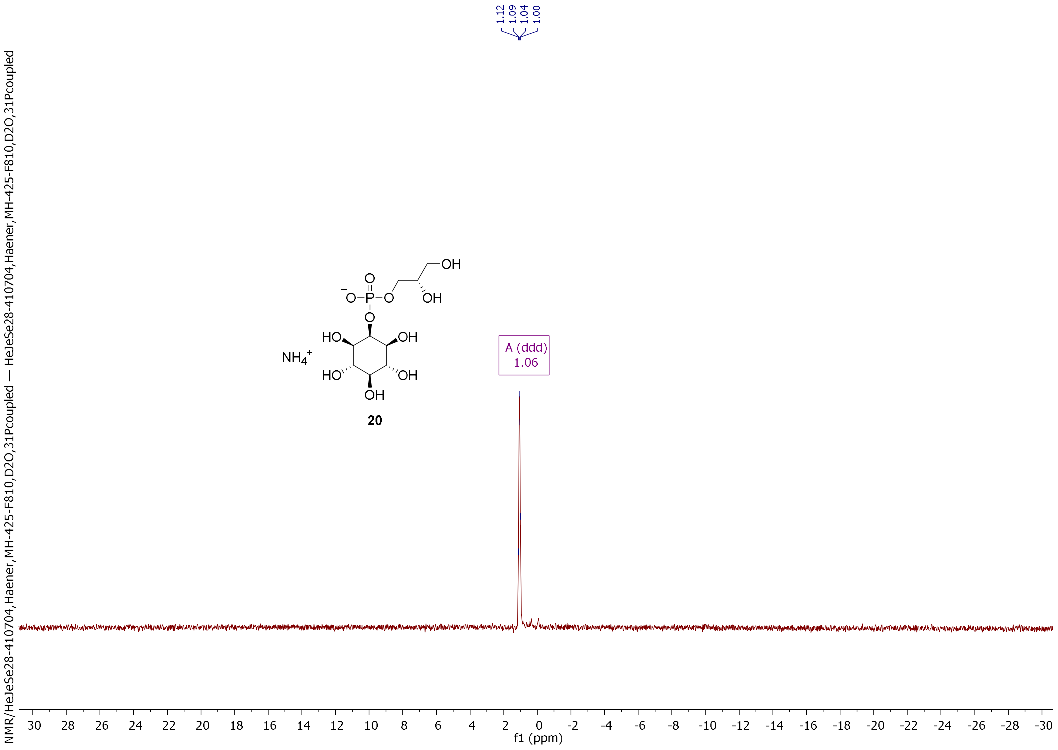

Supplementary Figure 56 ^{31}P-NMR of **20**.

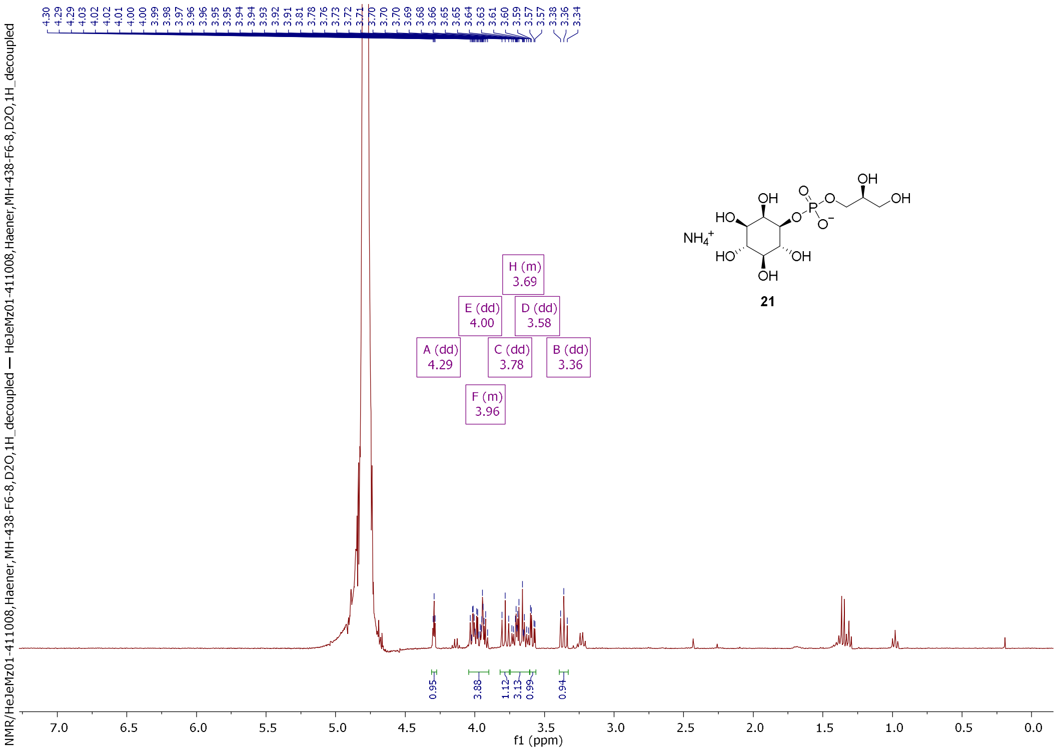

Supplementary Figure 57 ^{1}H-NMR of **21**.

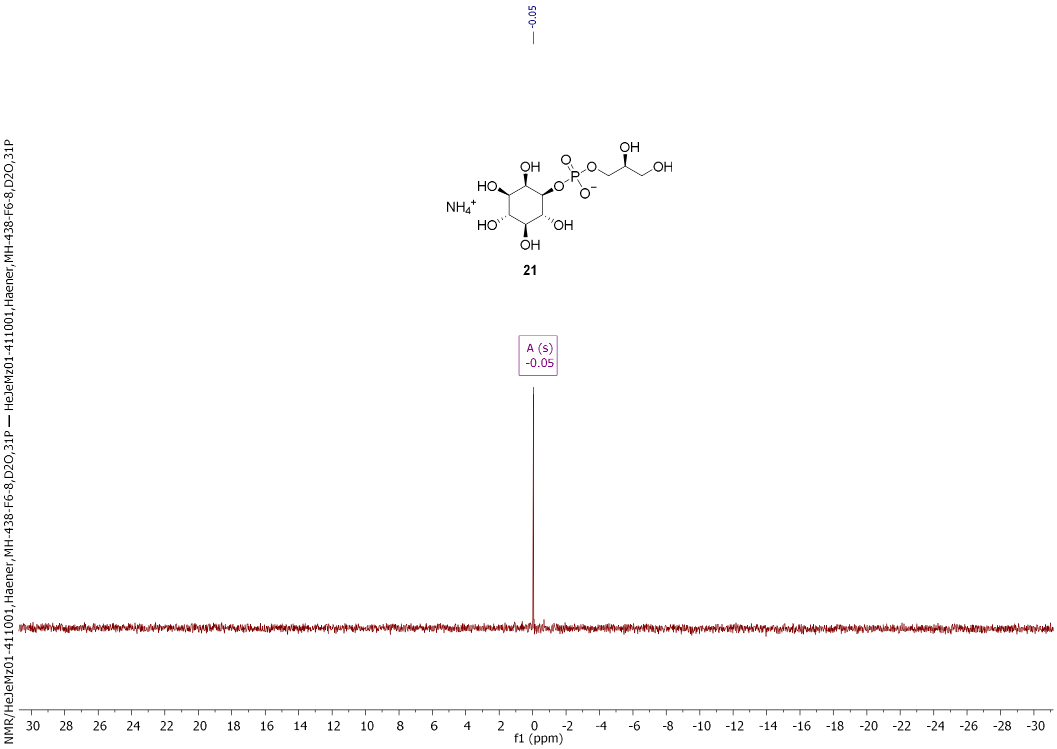

Supplementary Figure 58 ^{31}P{^{1}H}-NMR of **21**.

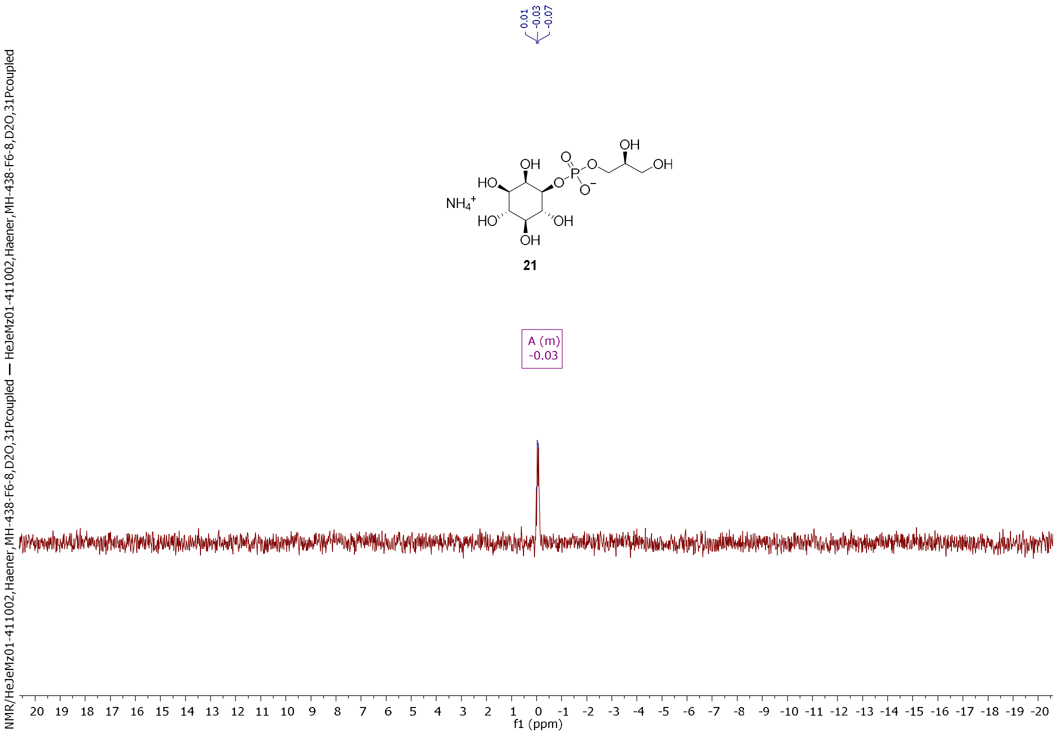

Supplementary Figure 59 ^{31}P-NMR of **21**.

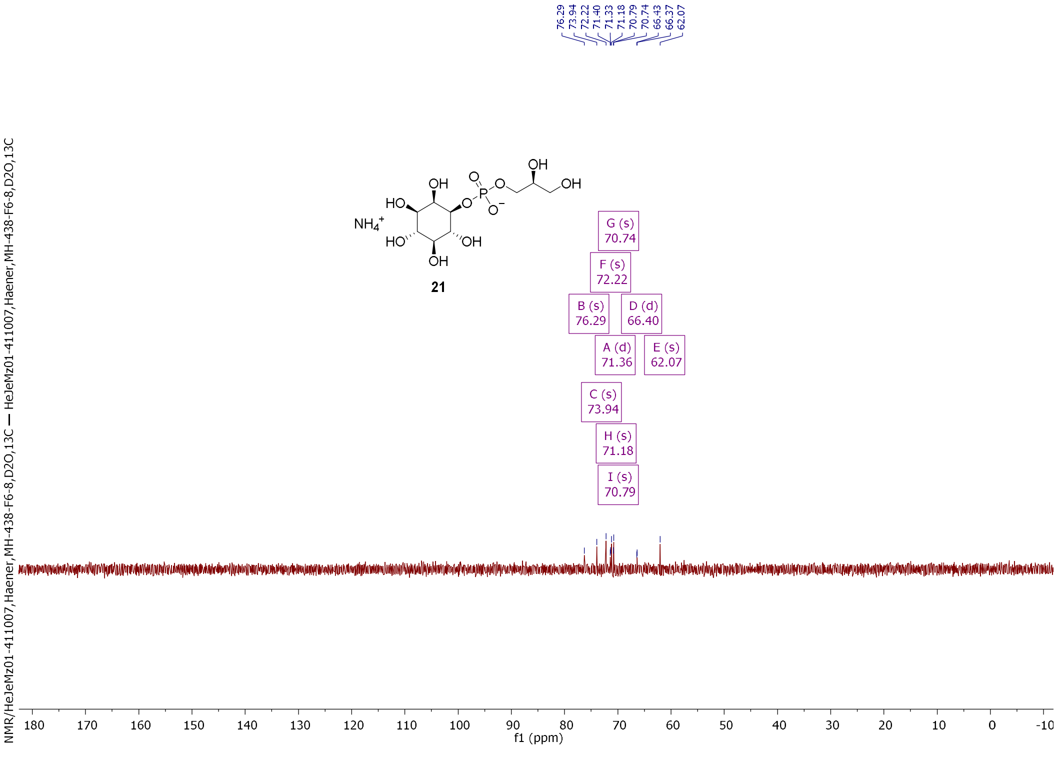

Supplementary Figure 60 ^{13}C-NMR of **21**.
